# Multi‐Omics Profiling Identifies Novel Therapeutic Targets and Pathobiological Mechanisms in Oral Cancer

**DOI:** 10.1111/jcmm.71272

**Published:** 2026-08-04

**Authors:** Meishan Huang, Hongwei Wang, Xiaoqiang Mo, Xiong Huang, Tao Liang, Xiuwen Mo, Minsi Li, Rong Fu, Xidai Long, Xuanping Huang

**Affiliations:** ^1^ College & Hospital of Stomatology Guangxi Medical University Nanning China; ^2^ Guangxi Key Laboratory of Oral and Maxillofacial Rehabilitation and Reconstruction Nanning China; ^3^ Department of Stomatology, Beihai Hospital of Chinese Medicine Beihai Hospital Affiliated to Guangxi University of Chinese Medicine Beihai Guangxi China; ^4^ Youjiang Medical University for Nationalities Baise Guangxi China; ^5^ Key Laboratory of Tumor Molecular Pathology of Baise Baise Guangxi China; ^6^ Medical College of Guangxi University Nanning Guangxi China

**Keywords:** drug safety, drug target, immune mediation, oral cancer, proteome‐wide Mendelian randomization

## Abstract

Oral cancer (OC) remains a therapeutic challenge due to limited validated targets. Cis‐pQTLs from the deCODE cohort (*n* = 35,559) were harmonized with OC‐GWAS (3547 cases and 691,466 controls) meta‐data through a two‐sample Mendelian randomization (MR) framework. Robust Validation included replication in the UKB‐PPP dataset, colocalization analysis, SMR (Summary‐based MR), HEIDI (Heterogeneity in Dependent Instruments) tests, and eQTL evidence. Additional analyses encompassed protein–protein interaction (PPI) networks, Kyoto Encyclopedia of Genes and Genomes annotation (KEGG)/Gene Ontology (GO) pathway enrichment, mediation, Druggability and side effects analysis. Oral cancer (OC) remains a therapeutic challenge due to limited validated targets. Cis‐pQTLs from the deCODE cohort (*n* = 35,559) were harmonized with OC‐GWAS (3547 cases and 691,466 controls) meta‐data through a two‐sample Mendelian randomization (MR) framework. Robust Validation included replication in the UKB‐PPP dataset, colocalization analysis, SMR (Summary‐based MR), HEIDI (Heterogeneity in Dependent Instruments) tests, and eQTL evidence. Additional analyses encompassed protein–protein interaction (PPI) networks, Kyoto Encyclopedia of Genes and Genomes annotation (KEGG)/Gene Ontology (GO) pathway enrichment, mediation, Druggability and side effects analysis. To experimentally corroborate the MR findings, the quantitative real‐time PCR (qRT‐PCR) was performed to examine the mRNA expression levels of selected genes in oral squamous cell carcinoma (OSCC) cell lines (SCC‐9 and SCC‐25) and normal human oral epithelial cells (HOEC). Multi‐omics MR identified TNFSF8 (*p* = 2.49 × 10^−8^, OR = 1.24) as a Tier 1 target. XXYLT1 (*p* = 1.41 × 10^−4^, OR = 1.34) and HSD17B14 (*p* = 0.04, OR = 2.00) achieved Tier 2. eQTLs‐pQTLs Relationship revealed XXYLT1 expression explained 24.65% of risk through elevated plasma protein levels, TNFSF8 eQTLs mediated 72.80% via protein upregulation. Mediation analyses on CD4 on HLA DR+ CD4+ T cells (7.18%) contributed to the risk of OC by upregulating plasma HSD17B14, while daily cigarette consumption (20.63%) and CD8+ T cell percentage leukocytes (12.06%) contributed to the risk of OC by upregulating plasma TNFSF8. Drug safety assessments highlighted systemic TNFSF8 inhibition may elevate skin cancer risk. Multi‐omics MR prioritizes TNFSF8 as a therapeutic target for oral cancer. Multi‐omics MR identified TNFSF8 (*p* = 2.49 × 10^−8^, OR = 1.24) as a Tier 1 target. XXYLT1 (*p* = 1.41 × 10^−4^, OR = 1.34) and HSD17B14 (*p* = 0.04, OR = 2.00) achieved Tier 2. eQTLs‐pQTLs Relationship revealed XXYLT1 expression explained 24.65% of risk through elevated plasma protein levels, TNFSF8 eQTLs mediated 72.80% via protein upregulation. Mediation analyses on CD4 on HLA DR+ CD4+ T cells (7.18%) contributed to the risk of OC by upregulating plasma HSD17B14, while daily cigarette consumption (20.63%) and CD8+ T cell percentage leukocytes (12.06%) contributed to the risk of OC by upregulating plasma TNFSF8. Drug safety assessments highlighted systemic TNFSF8 inhibition may elevate skin cancer risk. qRT‐PCR confirmed significant upregulation of XXYLT1 and HSD17B14 in both SCC‐9 and SCC‐25 cell lines (*p* < 0.001), which is consistent with MR predictions. TNFSF8 was not reliably detected in these cancer cells, aligning with its proposed immune‐mediated mechanism. Multi‐omics MR prioritizes TNFSF8 as a therapeutic target for oral cancer. qPCR validation confirms the dysregulation of XXYLT1 and HSD17B14, while findings regarding TNFSF8 underscore its immune origin. These findings warrant further functional studies.

**Abbreviations**
CD30L: CD30 ligand
eQTL: expression quantitative trait loci
FDR: false discovery rate
GAME‐ON: Genetic Associations and Mechanisms in Oncology Network
GO: Gene Ontology
GWASs: genome‐wide association studies
HEIDI: Heterogeneity in Dependent Instruments
HOEC: human oral epithelial cells
HSD17B14: 17β‐hydroxysteroid dehydrogenase type 14
KEGG: Kyoto Encyclopedia of Genes and Genomes annotation
lncRNA: long non‐coding RNA
MR: Mendelian randomization
OC: oral cancer
OSCC: oral squamous cell carcinoma
PPI: protein–protein interaction
pQTL: protein quantitative trait loci
qRT‐PCR: quantitative real‐time PCR
SCC‐25: human tongue squamous cell carcinoma cell line
SCC‐9: human tongue squamous cell carcinoma cell line
SMR: summary‐data‐based MR
TNF: tumour necrosis factor
TNFSF8: TNF superfamily member 8
UKB PPP: UK Biobank Plasma Proteome Project
UKB: UK Biobank
XXYLT1: xyloside xylosyltransferase 1

## 1 Introduction

Oral cancer (OC) is a prevalent malignancy among head and neck tumours, ranking as the sixth most common malignant tumour worldwide [1]. An epidemiological survey conducted in 2024 reported approximately 36,620 new cases and 7930 deaths attributed to OC [2]. Current treatment protocols for OC include a comprehensive regimen of surgery, radiotherapy, chemotherapy, immunotherapy, and targeted therapies. Despite significant advancements in these therapeutic approaches, these modalities possess unneglectable issues and adverse side effects [3]. adjuvant radiotherapy and chemotherapy is frequently associated with transient or permanent damage to healthy tissues, which profoundly compromise patient health and quality of life [4]. Consequently, there is an imperative need to explore and validate novel targeted therapies and develop new therapeutic targets that are effective against OC [5].

Proteins are multifunctional bioactive compounds involved in regulating a wide range of cellular and physiological functions. Innovations in next‐generation proteomics techniques have facilitated the identification of ectopic protein expression and the investigation of potential therapeutic targets for biomarker‐driven cancer treatments [6]. For instance, using a quantitative proteomics approach, Wu et al. [7] identified the kinase EPHA2 as a promising new therapeutic target for head and neck squamous cell carcinoma. Moreover, recent proteomic studies have revealed that downregulation of the hnRNP family proteins is a highly efficient target for anticancer activity in acute promyelocytic leukaemia [8], highlighting the increasing importance of proteomics in OC research. However, many of these studies are limited by small sample sizes and a narrow range of protein species examined. The causal relevance of these associations found in non‐randomized observational studies is challenging to determine and is susceptible to confounding factors or reverse causality [5].

Mendelian randomization (MR), which leverages genetic variants as proxies for protein levels, provides a robust framework for causal inference in epidemiological studies by mitigating biases from reverse causality and residual confounding [9, 10]. With the progress in genome‐wide association studies (GWASs) of the human plasma proteome, a sophisticated integrated framework combining genomic and proteomic data has been developed [5], significantly enhancing biomarker and drug targets discovery. In this context, we conducted a proteome‐wide MR analysis utilizing genome‐wide proteomics to establish the causal relationships between plasma proteins and OC. Furthermore, our study aimed to elucidate potential causal links between variable factors, proteins, and disease, explore the clinical applications of these proteins, and assess their potential as therapeutic targets.

## 2 Method

### 2.1 Study Design

**FIGURE 1:**
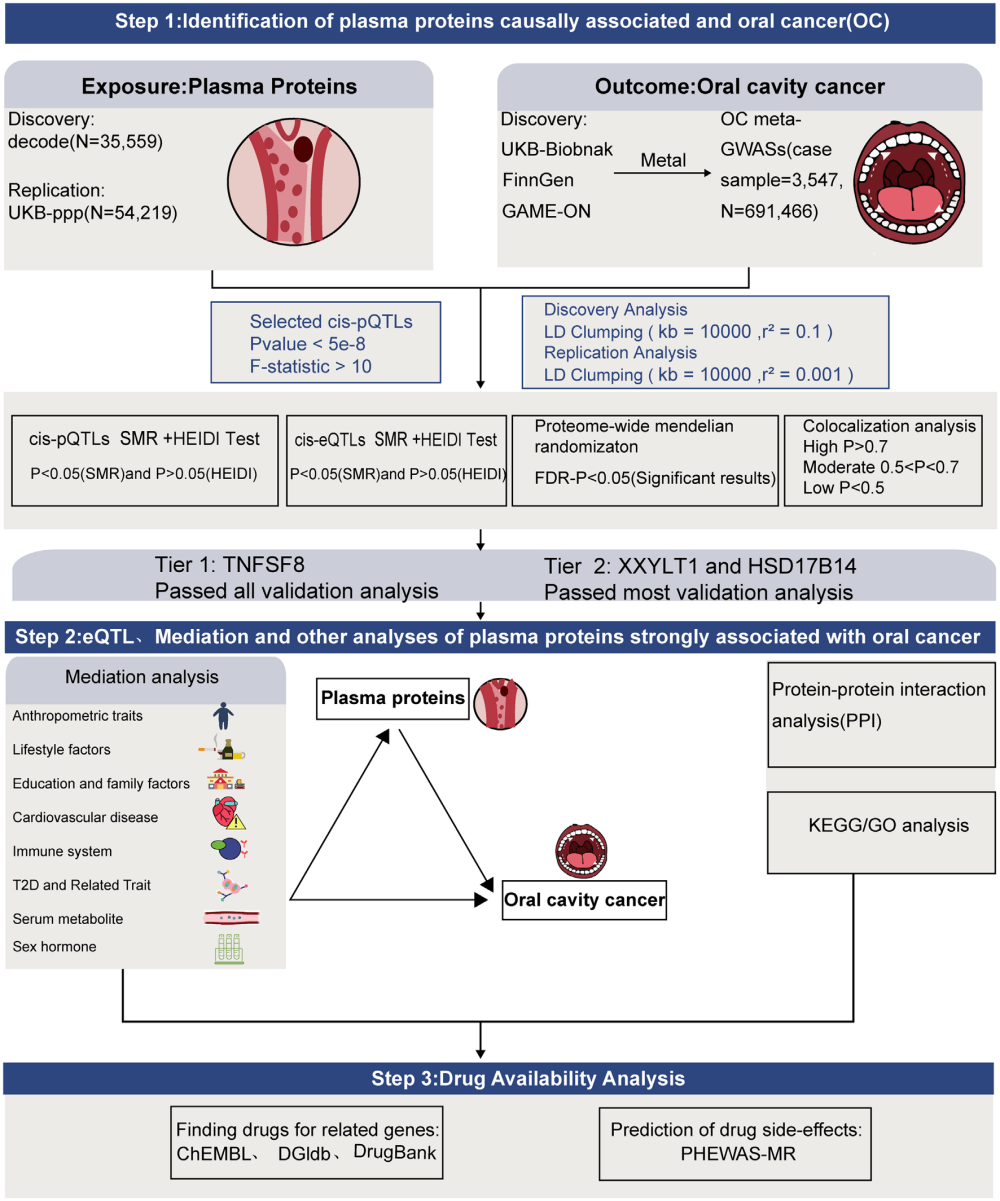
Flowchart of the study design. Step 1:Genome‐wide proteomic data from the deCODE cohort (*N* = 35,559) were integrated with oral cancer (OC) GWAS meta‐analyses (FinnGen, UK Biobank, GAME‐ON; 3547 cases, 691,466 controls). Genetic instruments (cis‐pQTLs) were selected under stringent criteria (genome‐wide significance, LD clumping, *F*‐statistic > 10) and validated through replication in the UK Biobank Plasma Proteome Project (UKB‐PPP). Multi‐tier validation (SMR, HEIDI tests, Bayesian colocalization) prioritized proteins into Tier 1 (TNFSF8: Full validation) and Tier 2 (XXYLT1, HSD17B14: Partial validation). Step 2: The identified Tier 1 and Tier 2 proteins underwent systematic biological characterization through: (1) mediator analysis to identify intermediate pathways, (2) protein–protein interaction (PPI) network mapping, and (3) functional annotation using KEGG/GO enrichment analyses. Step 3: Drug databases (ChEMBL, DGIdb, DrugBank) were screened for TNFSF8‐targeted therapies, while phenome‐wide MR (PHEWAS‐MR) predicted potential adverse effects. This figure outlines a systematic workflow from causal protein discovery to clinical translation, emphasizing rigorous validation (Tier1‐3), immune‐mediated mechanisms, and actionable therapeutic insights for oral cancer. MR, Mendelian randomization; cis‐pQTL, cis‐protein quantitative trait locus; LD, linkage disequilibrium.

The comprehensive study design is illustrated in Figure 1. A multi‐omics framework, integrating large‐scale datasets and multi‐tier validation strategies, was employed to pinpoint causal molecular drivers of OC and delineate their mechanistic contributions to disease pathogenesis.

### 2.2 Data Sources for OC Outcomes pQTL and eQTL

The resultant data were obtained from the UK Biobank (UKB), FinnGen10, and the Genetic Associations and Mechanisms in Oncology Network (GAME‐ON) [11]. All datasets included individuals of European ancestry. The UKB dataset encompasses 372,373 European men and women, consisting of 357 OC cases and 372,016 controls, updated in 2021. The FINN10 dataset comprises 315,025 samples, with 832 OC cases and 314,193 controls. Additionally, two additional datasets from GAME‐ON included 1223 cases with 2928 controls and 1135 cases with 2329 controls, respectively. Ultimately, we consolidated the OC‐GWAS data from these sources using GWAS meta‐analysis for further comprehensive analysis (sample case = 3547, *N* = 691,466).

pQTL and eQTLs data sources: A total of 4907 plasma proteins from the deCODE Consortium were utilized as the experimental exposure set. This dataset is derived from a pQTL study involving 35,559 Icelanders [12]. For the purpose of this study, Cis‐protein quantitative trait loci (Cis‐pQTL) were defined as variants located within a specified distance (e.g., within 1 Mb) of the transcription start site of the gene encoding the target protein, while all other associations were considered to represent potential trans effects. Given that Cis‐pQTL demonstrated greater efficacy compared to trans‐pQTL, Cis‐expression quantitative trait loci (Cis‐eQTL) were selected for further analysis. Additionally, to replicate the discovery set results, the UK Biobank Plasma Proteome Project (UKB‐PPP) (*N* = 54,219) acts as a duplicate set, enhancing the reliability of the results. To further investigate the relationship between eQTL and pQTL, corresponding Cis‐eQTL data were acquired from 31,684 individual blood samples from the eQTLGen consortium [13] for subsequent MR, Summary‐based MR (SMR), and Heterogeneity in Dependent Instruments (HEIDI) analyses.

ALL characteristics of the GWAS summary data can be obtained Table S1.

### 2.3 Mendelian Randomization and Sensitivity Analyses

Our study rigorously adheres to the STROBE‐MR guidelines [14]. The causal relationship between plasma proteins and OC was assessed using a two‐sample MR framework, following three core assumptions: (i) the relevance assumption: genetic instruments (Cis‐pQTLs) must strongly associate with protein levels (*p* < 5 × 10^−8^); (ii) the independence assumption: instruments must be independent of confounders (LD clumping: *r* ^2^ < 0.1, 10,000 kb window) and (iii) the exclusion restriction assumption: instruments influence outcomes solely via the exposure (no horizontal pleiotropy) [15]. SNPs were selected as instruments if they passed genome‐wide significance (*p* < 5 × 10^−8^) and had an *F*‐statistic > 10 to mitigate weak instrument bias. For replication, stricter LD thresholds (*r* ^2^ < 0.001) were applied (Figure 1). Details of the final IVs are provided in Table S2.

Primary MR estimates were derived using inverse‐variance weighted (IVW) regression when multiple SNPs were available, supplemented by Wald ratio for single‐SNP associations.

Sensitivity analyses included: (i) SMR/HEIDI tests: To validate causality and exclude pleiotropy [16, 17] (HEIDI‐*p* > 0.05). (ii) To assess the presence of horizontal pleiotropy, a pleiotropy test was conducted. If the pleiotropy test yielded a *p*‐value of less than 0.05, indicating potential pleiotropic bias, the MR‐PRESSO or radial‐MR method was subsequently applied; MR‐PROSSO and radial‐MR: To detect and remove outlier SNPs with pleiotropic effects, compared to MR‐PROSSO, radial‐MR was more advantageous [18]. In the analytical procedure, we first employed the MR‐PRESSO method to identify and exclude outlier variants. If evidence of pleiotropy persisted following this correction, radial‐MR was then applied iteratively until the pleiotropy test no longer showed statistical significance (i.e., *p* > 0.05). (iii) Reverse MR: To rule out reverse causation. Statistical significance was defined as false discovery rate *p*‐value < 0.05 (FDR‐*p* < 0.05), with nominal significance at *p* < 0.05 [19]. For Multi‐tier validation, stricter *p*‐value thresholds (*p* < 0.05) were applied.

### 2.4 Co‐Localization Analysis

To further validate the causal relationship between the identified positive plasma proteins and the outcome, a co‐localization analysis was performed. This analysis utilized a species hypothesis framework to explore potential associations, outlined as follows: (i) H0: no association with either trait; (ii) H1: association with trait 1, but not with trait 2; (iii) H2: association with trait 2, not with trait 1; (iv) H3: association with trait 1 and trait 2, two independent SNPs; (v) H4: association with trait 1 and trait 2, one shared SNP [20]. For the pQTL‐GWAS analysis, the co‐localization region window was defined as ±1 MB. Five different posterior probabilities were assigned to each hypothesis to evaluate the evidence of co‐localization. The results for hypothesis H4 were categorized based on their posterior probabilities as follows: greater than 0.7 indicated high co‐localization evidence, between 0.5 and 0.7 suggested medium co‐localization evidence, and less than 0.5 denoted low evidence [21].

### 2.5 Integration of Multi‐Omics Evidence Grading

Validation of candidate targets incorporated SMR, replication set analysis, HEIDI tests, Bayesian colocalization and eQTL evidence. Targets were hierarchically classified into three tiers based on validation stringency: The results were as follows: all these grades were first required to pass MR analysis (*p* < 0.05): (1) Tier 1 motifs passed all the validation analyses mentioned above (including SMR analysis, replica set analysis, HEIDI test, eQTL Evidence, co‐localization analyses), and (PH4 > 0.5); (2) Tier 2 required validation in ≥ 4 criteria; (3) Tier 3 motifs are motifs that do not reach the remaining proteins in Tier 1 and Tier 2.

### 2.6 Variable Factors‐Plasma Proteins‐Outcome: Two‐Step Analysis

A two‐step analytical approach was implemented to further explore the mediating role of identified positive proteins between variable factors and outcomes. The variable factors considered in this study were divided into eight categories, including physical condition, immune system response, lifestyle choices, and endocrine conditions, among others, as detailed in Table S1. Instrumental variables were selected based on stringent criteria: a significance threshold of *p <* 5 × 10^−8^, LD of 0.001, and a proximity condition of 10,000 kilobases (kb). In the first step of the analysis, the effect size of the association between variable factors and proteins was calculated as *β*1, while in the second step, the effect size of the association between proteins and outcomes was computed as *β*2. The mediating effect was then quantified as *β*1*β2 [22]. The mediation efficiency ratio is calculated as β1*β2/β where *β* represents the effect size of the association between the variable factors and the outcome. Furthermore, the two‐step MR approach is bound by the three core MR assumptions, and the detection and management of pleiotropy follow the same principles as in two‐sample MR.

### 2.7 Protein–Protein Interaction (PPI) Networks and Enrichment Analysis

Interactions between candidate proteins were explored using the STRING database (https://string‐db.org/). The network edge selection was based on ‘CONFIDENCE’ with a minimum interaction score threshold of 0.7, and only up to 50 interactors were included in the first shell. Additionally, OC expression data from the TCGA database was subjected to Pearson correlation analysis to identify the top 100 genes closely related to the identified positive proteins. Subsequent Kyoto Encyclopedia of Genes and Genomes annotation (KEGG)/Gene Ontology (GO) analysis was conducted to elucidate the biological functions of these proteins and the pathways in which they are involved.

### 2.8 Drug Availability Prediction and Its Side Effect Analysis

To assess the drug interaction profiles of target proteins, searches were conducted in the ChEMBL database [23], DGIdb [24] and Drug Bank [25] to identify potential interactions between these proteins and drugs. Based on this information, disease summary statistics from the UKB cohort, excluding outcomes with fewer than 1000 cases in dichotomous outcomes and total counts below 1000 in continuous outcomes, were utilized for phenome‐wide MR (PHEWAS‐MR) analysis. This approach was employed to evaluate the safety of these proteins as potential therapeutic targets and was aimed at exploring potential drug‐related side effects. FDR‐*p* < 0.05 was considered significant.

All analyses were conducted using the ‘TwoSampleMR’ R package, ‘MR‐PROSSO’ R package and ‘coloc’ R package. with pleiotropy adjustments performed using the ‘RadialMR’ package.

### 2.9 Cell Experiments

The human tongue squamous cell carcinoma cell lines (SCC‐9 and SCC‐25) and the normal human oral epithelial cell line (HOEC) were obtained from previously stored by our research group which previously has stored the cell lines. HOEC cells were cultured in DMEM (KeyGEN BioTECH, China) supplemented with 10% fetal bovine serum (FBS; KeyGEN BioTECH) and 1% penicillin–streptomycin. SCC‐9 and SCC‐25 cells were cultured in DMEM/F12 (KeyGEN BioTECH, China) supplemented with 10% fetal bovine serum (FBS; KeyGEN BioTECH) and 1% penicillin–streptomycin. All cells were maintained at 37°C in a humidified atmosphere containing 5% CO_2_.

Total RNA was extracted from cells using an RNA extraction kit (SEVEN BioTECH, China) according to the manufacturer's protocol. The concentration and purity of RNA were determined using the NanoDrop spectrophotometer (Thermo Fisher Scientific, USA). Complementary DNA (cDNA) was synthesized from 1 μg of total RNA using the All‐in‐one First Strand cDNA Synthesis Kit II (SEVEN BioTECH, China) per the manufacturer's instructions.

RT‐qPCR quantified the mRNA expression levels of TNFSF8, XXYLT1, and HSD17B14. A 20 μL reaction mixture was prepared using the 2 × SYBR Green qPCR MasterMix II (SEVEN BioTECH, China) on a Quant Studio instrument (Thermo Fisher Scientific, USA). The specific primer sequences used were as follows:

TNFSF8 (target) F: CAAGCACTCAACGGAATGGC.

TNFSF8 (target) R: CGTCCTCTGAACGACCAACA.

XXYLT1 (target) F: GCCAGGCAGAACTGTCATCT.

XXYLT1 (target) R: CTGCAGGATCTCTTTGGGCA.

HSD17B14 (target) F: TGGTTTCTGAGACCATCCGC.

HSD17B14 (target) R: GTCAAGGTGTACGTCCCCAG.

GAPDH (internal control) F: ACACCCACTCCTCCACCTTTG.

GAPDH (internal control) R: TCCACCACCCTGTTGCTGTAG.

Thermal cycling conditions consisted of 95°C for 30 s; then, 40 cycles of 95°C for 15 s and 60°C for 20 s. A melting curve analysis was performed to verify the specificity of the amplification. The relative gene expression levels were calculated using the $2^{-∆∆C_{T}}$ method and normalized to the internal control (GAPDH). All experiments were performed in triplicate, representing three independent biological replicates.

## 3 Results

### 3.1 Proteome‐Wide MR Analysis

**FIGURE 2:**
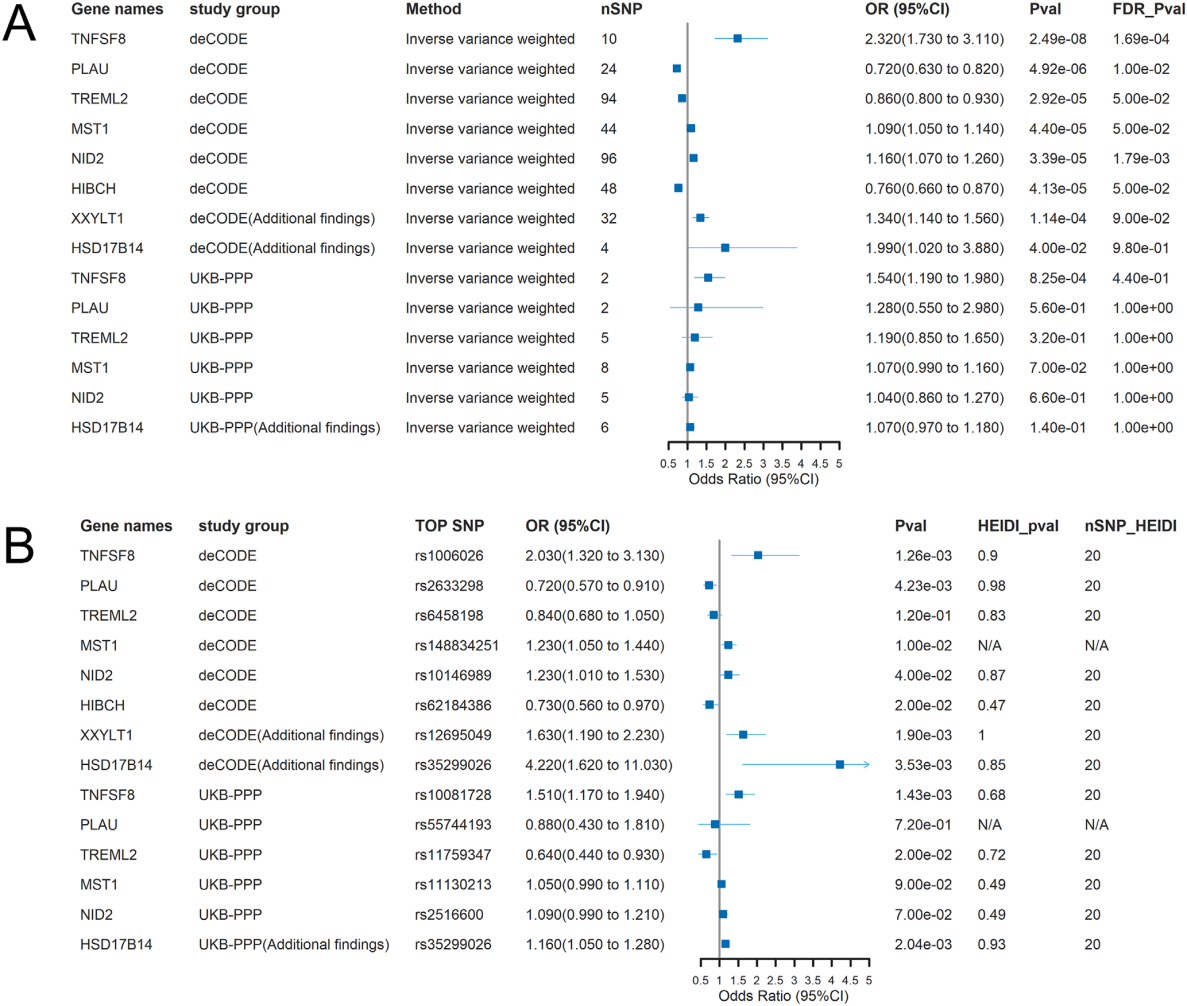
(A) Results of MR of 8 plasma proteins. OR, odds ratio; nsnp, number of Single‐nucleotide polymorphism; 95% CI, 95% confidence interval; FDR, false discovery rate; nSNP, number of SNPs. (B) Results of SMR of 8 plasma proteins. OR, odds ratio; HEIDI, HEIDI test; nSNP_HEIDI, Number of SNPs used to calculate HEIDI TEST; SNP, single‐nucleotide polymorphism.

6 plasma proteins exhibited a nominally significant causal relationship with OC (FDR‐*p* < 0.05); Specifically, TNFSF8, MST1, and NID2 were promoters of OC, and conversely, PLAU, TREML2, and HIBCH were protectors of OC. TNFSF8 were replicated in the validation set. In this analysis, XXYLT1 and HSD17B14 reached nominal significance (*p* < 0.05) and were brought to our attention because they appeared to replicate relatively well in subsequent validations (Figure 2A and Table 1). When sensitivity analyses were performed, NID2 and PLAU showed significant pleiotropic effects After removal of the aberrant SNPs, there was no significant pleiotropic effect for NID2, whereas there was still a pleiotropic effect for PLAU (Table S3). In addition, the results of PLAU should be treated with caution considering that PLAU shows OR in the opposite direction in the discovery and replication sets (Figure 2A and Table S3). Reverse MR analysis indicated that the findings are unlikely to be influenced by reverse causality (Table S4).

**TABLE 1:** Multiple validation results of oral cancer‐related proteins.

Gene names	Protein names	Outcome	Replicationa	SMR (HEIDI) test	SMR (HEIDI) test	Colocalization	Colocalization	eQTL Evidence^f^	Egger_intercept	Egger_intercept	Cochran's Q	Cochran's Q
			UKB‐PPP^a^	deCODE^b^	UKB‐PPP^c^	deCODE^d^	UKB‐PPP^e^		Test (deCODE)^g^	Test (UKB‐PPP)^h^	Test (deCODE)^i^	Test (UKB‐PPP)^j^
TNFSF8	CD30 Ligand	Oral Cancer	√	√*	√*	√	√	√*	√	NA	√	√
PLAU	uPA	Oral Cancer	×	√*	×^	×	×	√*	×	NA	√	√
TREML2	TREML2	Oral Cancer	×	×*	√*	×	×	√*	√	√	√	√
MST1	MSP	Oral Cancer	×	√^	×*	×	×	×	√	√	√	√
NID2	NID2	Oral Cancer	×	√*	×*	×	×	×	√	√	√	√
HIBCH	HIBCH	Oral Cancer	×	√*	NA	×	×	×*	√	NA	√	NA
XXYLT1	XXLT1	Oral Cancer	NA	√*	NA	√	√	√*	√	√	√	NA
HSD17B14	DHB14	Oral Cancer	×	√*	√*	√	√	×	√	√	√	√

Abbreviations: √, pass; ×, fail; HEIDI, Heterogeneity in Dependent Instruments; MR, Mendelian randomization; NA, not available; pQTL, protein quantitative trait loci; SMR, Summary‐based MR.

^a^Replication in the UKB Plasma Proteome Project (UKB‐PPP; *N* = 54,219) applied stricter LD thresholds (*r* ^2^ < 0.001, kb = 10,000). Protein data from UKB‐PPP was utilized for replication analysis with the same outcome data to further consolidate the findings. Pass: in the replication set, the *p*‐value < 0.05, and the effect values were essentially the same as those in the discovery set. ^b^Summary‐based MR is an extended approach from MR and can be used as another validation method to consolidate the findings of MR. HEIDI, on the other hand, is used to verify the presence of heterogeneity in the results. Pass: SMR: *p*‐value < 0.05. *HEIDI Test *p*‐value > 0.05;^HEIDI Test *p*‐value = NA. ^c^The data for this SMR and HEIDI test were obtained from the UKB‐PPP and were used to validate the deCODE results and reduce false positives. Pass SMR: *p*‐value < 0.05. *HEIDI Test *p*‐value > 0.05; ^HEIDI Test *p*‐value = NA. ^d^Co‐localization analyses were used to interpret whether exposure and outcome were driven by the same genetic variant. Considering the high false‐negative rate of Bayesian co‐localization analysis, we set 0.5 < *p* < 0.7 as moderate evidence and *p* > 0.7 as high evidence. pass: PP.H4 > 0.5, means that there is evidence that the exposure and outcome have the same genetic variation. ^e^To minimize stochastic bias, a uniform colocalization validation framework was consistently applied to all UKB‐PPP‐derived associations. Pass: PP.H4 > 0.5 means that there is evidence that the exposure and outcome have the same genetic variation. ^f^The eQTLs were utilized at the gene level to determine if they were associated with OC. Pass: SMR *p*‐value < 0.05 and HEIDI Test *p*‐value > 0.05; *MR‐*p‐*value < 0.05. ^gh^Egger_intercept is used to Evaluate the potential presence of pleiotropy in MR analysis from deCODE or UKB‐PPP. pass: *p* > 0.05. meaning that MR results are not affected by multiple effects. ^ij^Cochran's Q test reflecting the presence of heterogeneity among instrumental variables. pass: *p* > 0.05. meaning that no heterogeneity arises between exposure and outcome causality. ^ghij^Quality control of MR analysis in deCODE or UKB‐PPP.

### 3.2 SMR and HEIDI Tests Validated 8 Proteins

Based on the MR analysis, we identified TNFSF8, XXYLT1, LHSD17B14, PLAU, NID2, MST1 and HIBCH plasma proteins that were causally associated with OC (*p* < 0.05); further validation was conducted using a validation set, which resulted in the successful replication of TNFSF8, TREML2 and HSD17B14 plasma proteins. Replicates were not obtained due to missing relevant data in XXYLT1. HEIDI tests indicated non‐significant horizontal pleiotropic effects of 7 proteins (HEIDI‐*p* > 0.05), as depicted in Figure 2B and Table S5. suggesting their potential prioritization as therapeutic targets.

### 3.3 Co‐Localization Results Support a Causal Relationship Between Plasma Proteins and OC

In the experimental set, three of the nine plasma proteins significantly associated with OC displayed strong evidence of gene co‐localization. Specifically, TNFSF8 indicated closest high‐level evidence (H4 > 0.6), while HSD17B14, XXYLT1, each displayed medium‐level evidence (0.5 < H4 < 0.6). NID2, TREML2, MST1, HIBCH and PLAU exhibited low‐level evidence (H4 < 0.5) (Table S6).

**FIGURE 3:**
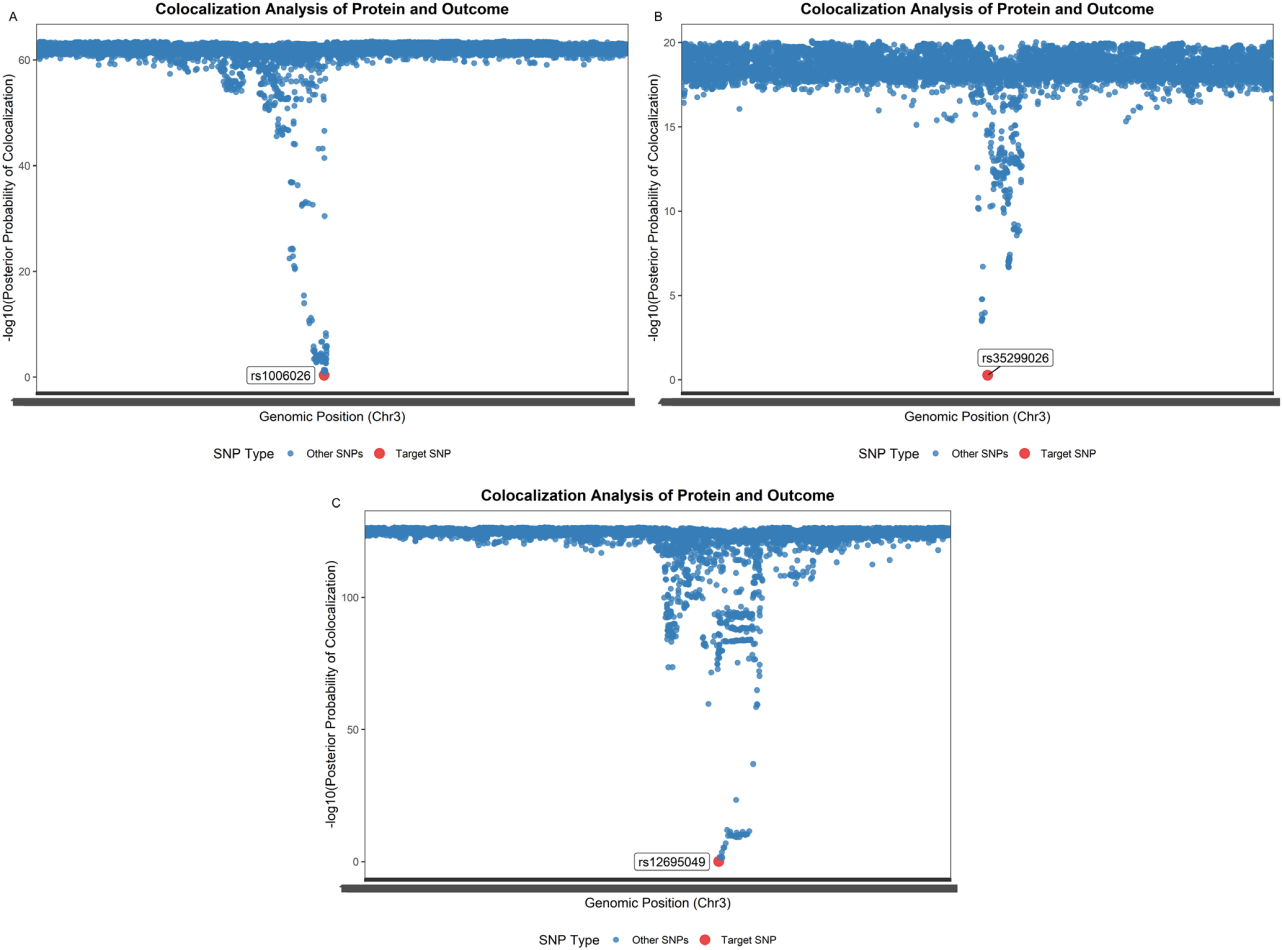
Visualization of 3 plasma proteins co‐localization results. A: Regional association plot of CD30 ligand protein (TNFSF8) with OC risk. Dominant SNPs are shown as red dot. SNPs within ±1 MB of the protein quantitative trait locus are included. (B) Regional association plot of DHB14 protein (HSD17B14) with OC risk. Dominant SNPs are shown as red dot. SNPs within ±1 MB of the protein quantitative trait locus were included. (C) Regional association plot of XXLT1 protein (XXYLT1) with oral cancer risk. Dominant SNPs are shown as red dot. SNPs within ±1 MB of the protein quantitative trait locus were included.

In the validation set, Results from the discovery set were largely replicated, with PPH4 > 0.5 for HSD17B14 and TNFSF8. however, data for XXYLT1 were missing. Finally, the Co‐localization of the above plasma proteins was visualized and is depicted in Figure 3 and Table S6.

### 3.4 Evidence From eQTL

To gain a more comprehensive and direct understanding of how gene expression regulation affects disease outcomes. The results indicated that TNFSF8, XXYLT1, TREML2 and PLAU were significantly causally associated with OC, with HEIDI tests confirming that this association was not due to LD (HEIDI‐*p* > 0.05). Sensitivity analyses identified horizontal pleiotropy for MST1 and HIBCH, alongside significant heterogeneity for PLAU. MR results with outlier SNP exclusion confirmed robust causal associations for XXYLT1, TNFSF8, PLAU, and TREML2 (Table S7).

### 3.5 Stratification of Candidate Therapeutic Proteins

Based on a multi‐tier validation framework integrating MR, SMR, replication, and colocalization evidence, TNFSF8 was classified as a Tier 1 therapeutic target, demonstrating consistent causality across all validation modalities. XXYLT1 and HSD17B14 achieved Tier 2 status, showing robust but incomplete validation (≥ 4 criteria). The rest of the proteins passed only MR or SMR validation and therefore belong to tier 3 (Table 1).

### 3.6 Results of the PPI Network and KEGG/GO Enrichment Analysis

PPI analysis was performed on TNFSF8, XXYLT1, and HSD17B14. Although no direct interactions were observed among these three proteins, their respective interacting partners indicated that TNFSF8 is associated with the immune system, XXYLT1 is involved in glycosylation, and HSD17B14 belongs to the 17β‐hydroxysteroid dehydrogenase family; these findings are generally consistent with subsequent analyses (Figure S1). According to the KEGG/GO analysis, the results indicated that these proteins collectively participate in pathways primarily related to the immune system (Figures S2–S7).

### 3.7 Relationship Between eQTL and pQTL

**FIGURE 4:**
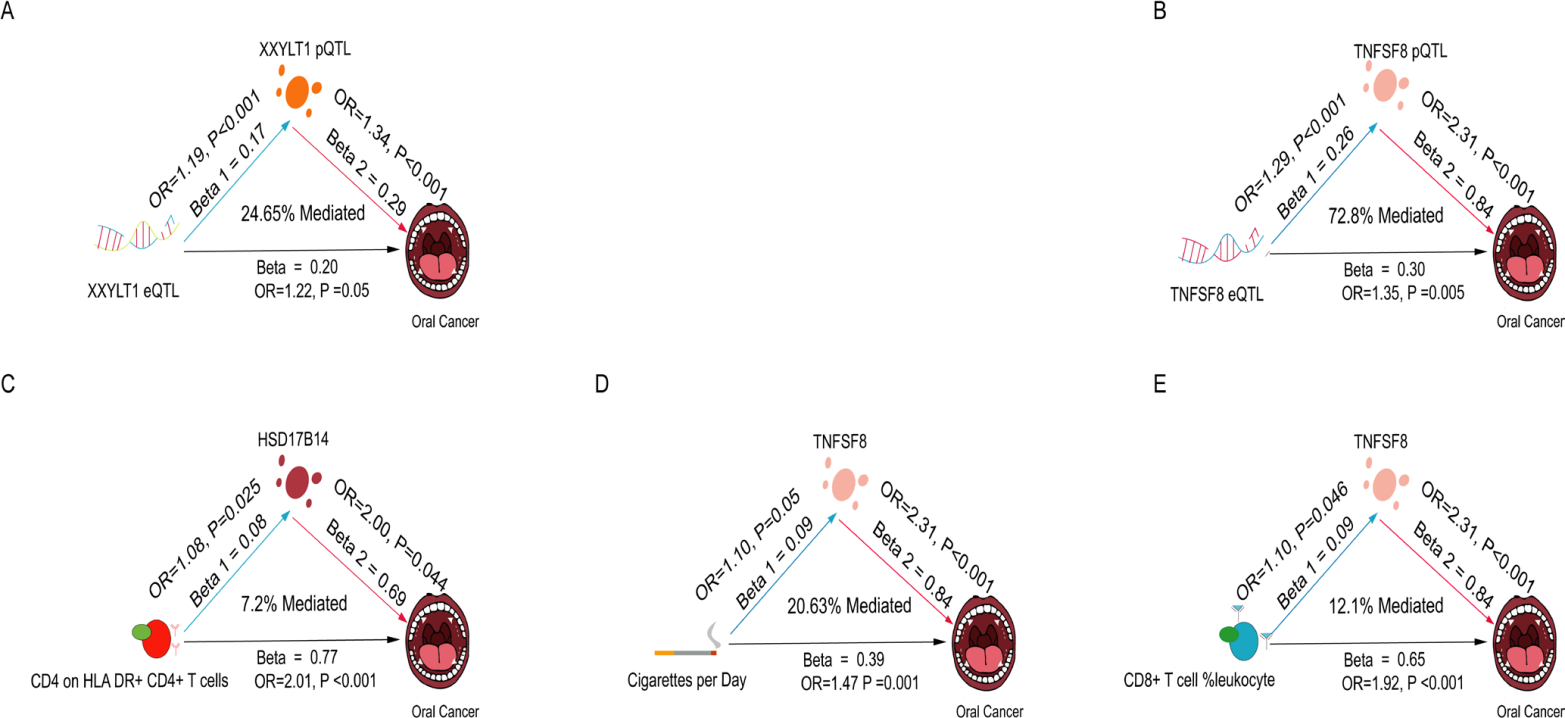
Visualization of results of Mediation analysis. (A) XXYLT1 eQTL accounted for 24.65% of OC risk via elevated XXYLT1 Protein Levels. (B) TNFSF8 eQTL mediated 72.80% of OC risk through TNFSF8 Protein Levels upregulation OC. (C) CD4+ T cells accounted for 7.2% of OC risk via elevated HSD17B14 expression. (D) Cigarettes per Day mediated 20.63% of OC risk through TNFSF8 upregulation. (E) CD8+ T cells mediated 12.06% of OC risk through TNFSF8 upregulation. OC, oral cancer; OR, odds ratio; Beta, efficiency value; Mediated, mediated effect ratios.

To investigate the causal mechanisms linking transcriptional regulation to oral cancer pathogenesis, we implemented a two‐step MR framework to evaluate plasma proteins as potential mediators between eQTLs and disease risk. Results revealed that XXYLT1 expression explained 24.65% (*p* = 4.63 × 10^−4^) of OC risk through elevated plasma XXYLT1 protein levels. TNFSF8 eQTLs mediated 72.80% (*p* = 8.53 × 10^−13^) of OC risk via TNFSF8 protein upregulation (Tables S8 and S9, Figure 4A,B). Plasma eQTLs for TNFSF8 and XXYLT1 exhibited regulatory effects on their protein abundances, directly. In contrast, HSD17B14 showed no significant eQTL‐pQTL association, suggesting its pathogenic role in OC may involve non‐genomic mechanisms such as post‐translational modifications.

### 3.8 Relationship Between Variable Risk Factors‐Protein‐Outcomes

In the MR analyses of 59 risk factors, 12 were found to be causally related to OC. Notably, 8 of the 12 were identified as promoting OC risk. including prior alcohol consumption (10‐year history), daily cigarette use, current tobacco smoking, CD4 on HLA DR+ CD4+ T cells, CD8 on CD28+ CD45RA‐CD8+ T cells, CD45RA+ CD8+ T cell absolute count, CD28 on CD45RA‐CD4 not regulatory T cells, and CD8+ T cell percentage leukocytes. Smoking and alcohol intake are particularly recognized as significant risk factors for OC (*p* < 0.05). Conversely, 4 of the 12 were suggested to have a protective role against OC development, including daytime napping, histidine, effector memory CD4+ T cell percentage, and testosterone levels (Table S8). Notably, the association between daytime napping, histidine levels, and OC pathogenesis has not been previously documented in epidemiological studies. For immune cells not causally associated with OC, refer to (Table S10) Sensitivity analyses indicated non‐significant heterogeneity or pleiotropic effects (Table S11).

When MR analyses were conducted between risk factors and plasma proteins, it was demonstrated pleiotropic associations between TNFSF8 expression and daily tobacco use. After adjusting for horizontal pleiotropy, MR confirmed causal effects of daily cigarette consumption on TNFSF8 upregulation. Mediation analyses demonstrated that CD4 on HLA DR+ CD4+ T cells contributed to the risk of OC by upregulating plasma HSD17B14 levels (*p* = 0.03, 95% CI: 1.01–1.16), while daily cigarette consumption (*p* = 0.05, 95% CI: 1.00–1.21) and CD8+ T cell percentage leukocytes (*p* = 0.05, 95% CI: 1.00–1.20) contributed to the risk of OC by upregulating plasma TNFSF8 levels. respectively, with mediation efficiencies of 7.18%, 20.63% and 12.06% (Table S12 and Figure 4C–E). our findings underscore the critical role of cigarette consumption the immune system in OC pathogenesis, nominating TNFSF8 as a tractable therapeutic target given its central mediation role.

### 3.9 Drug Availability Prediction and Side Effects

**FIGURE 5:**
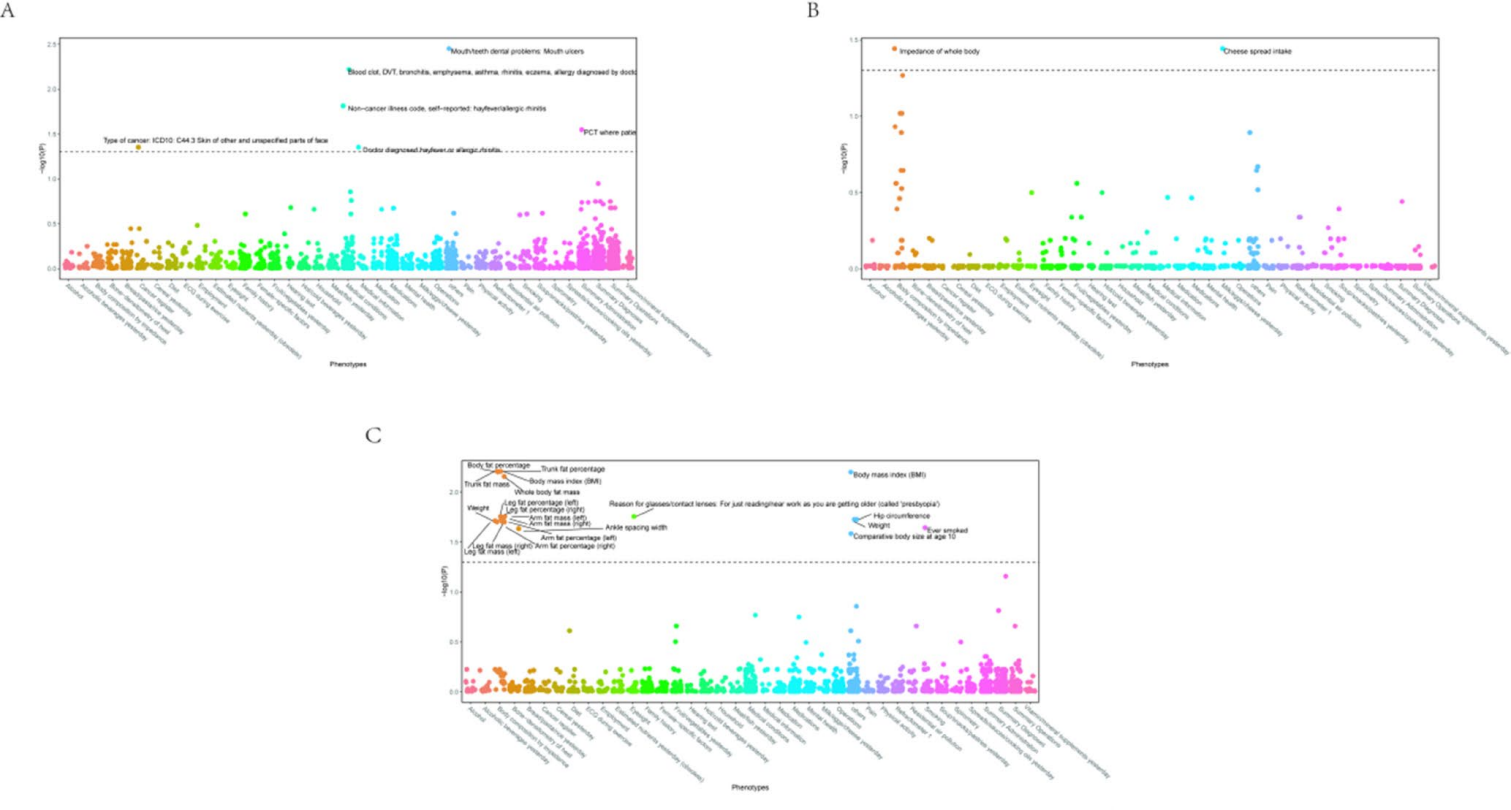
Results of phenome‐wide MR of 3 plasma proteins. (A) Manhattan plot of phenome‐wide MR results for (TNFSF8) CD30 ligand. The horizontal coordinates represent the categories for each phenotype. The dotted black line in the figure indicates the threshold for an FDR‐*p* value < 0.05. When a phenotype's measure exceeds this line, it is considered significant, indicating the presence of the corresponding side effect. (B) Manhattan plot of phenome‐wide MR results for (HSD17B14) DHB14. The horizontal coordinates represent the categories for each phenotype. The dotted black line in the figure indicates the threshold for an FDR‐*p* value < 0.05. When a phenotype's measure exceeds this line, it is considered significant, indicating the presence of the corresponding side effect. (C) Manhattan plot of phenome‐wide MR results for (XXYLT1) XXLT1. The horizontal coordinates represent the categories for each phenotype. The dotted black line in the figure indicates the threshold for an FDR‐*p* value < 0.05. When a phenotype's measure exceeds this line, it is considered significant, indicating the presence of the corresponding side effect.

Drug predictions for candidate proteins were conducted using three databases. Three candidate targets were not found to be relevant drugs. HSD17B14 were exhibited non‐significant side effects. However, TNFSF8 was associated with potential side effects such as skin or facial cancer, oral ulcers, and allergic rhinitis. In contrast, XXYLT1 was linked to weight changes, potentially leading to obesity (FDR‐*p* < 0.05) (Figure 5 and Table S13 for more details).

### 3.10 Experimental Validation of Hub Gene Expression in Oral Squamous Cell Carcinoma Cell Lines

To experimentally validate the differential expression of the hub genes identified by our bioinformatics analysis, we conducted qRT‐PCR on oral squamous cell carcinoma cell lines. Based on our in silico findings, we selected TNFSF8, XXYLT1, and HSD17B14 for validation, as they were among the top hub genes with the highest degree of connectivity and exhibited significant dysregulation in OSCC tissues.

**FIGURE 6:**
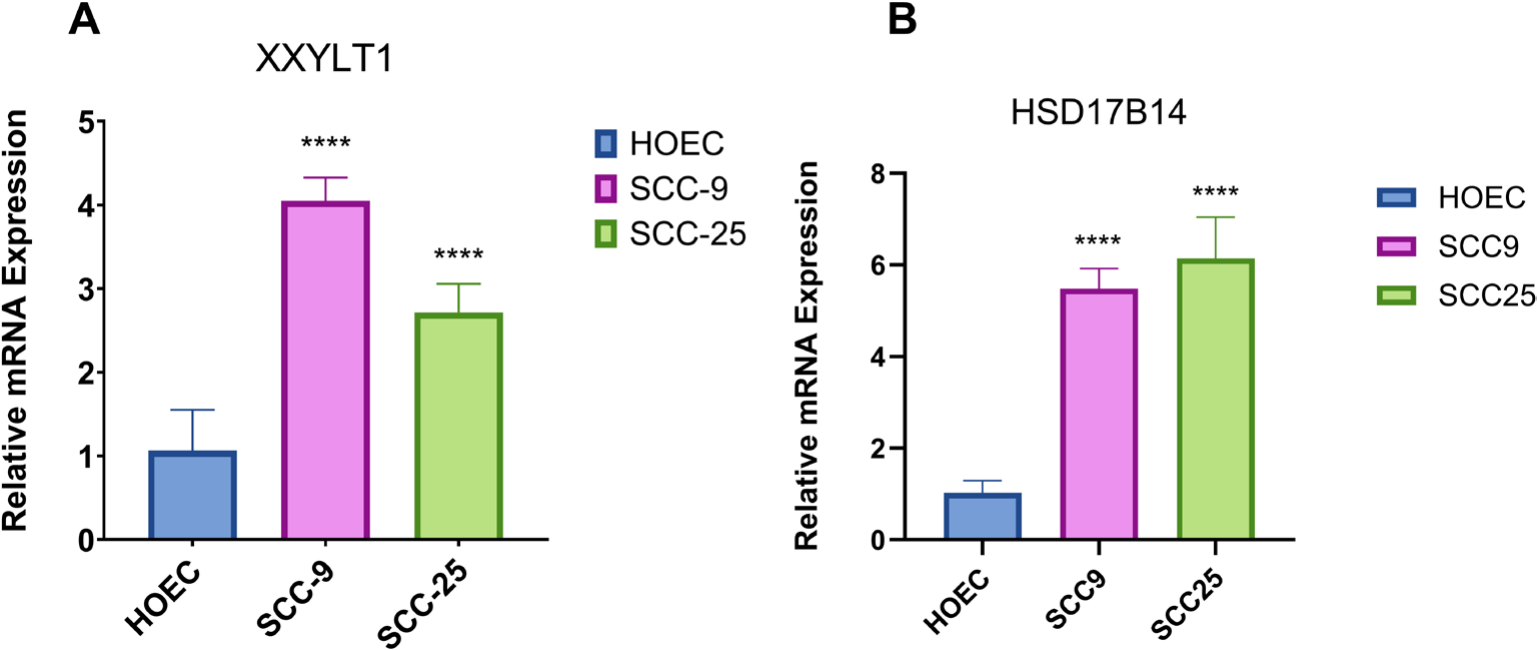
qRT‐PCR validation of hub gene expression in oral squamous cell carcinoma cell lines. A: XXYLT1 expression was validated in OSCC cell lines using qRT‐PCR. XXYLT1 mRNA levels were significantly higher in SCC‐9 and SCC‐25 cells than those in normal HOEC cells (****p < 0.001). AXYLT1 expression levels were normalized against GAPDH. B: HSD17B14 expression was validated in OSCC cell lines using qRT‐PCR. HSD17B14 mRNA levels were significantly higher in SCC‐9 and SCC‐25 cells than those in normal HOEC cells (****p < 0.001). HSD17B14 expression levels were normalized against GAPDH.

Consistent with our bioinformatics predictions, the mRNA expression levels of XXYLT1 and HSD17B14 were significantly higher in both SCC‐9 and SCC‐25 OSCC cell lines compared to normal HOECs (*p* < 0.001, Figure 6A,B). However, TNFSF8 could not be reliably detected via specific amplification in the tested cell lines, even after multiple optimization attempts with different primer sets and PCR conditions.

## 4 Discussion

In this comprehensive multi‐omics analysis, by integrating plasma pQTL, blood eQTL data, and OC GWAS data, we identified 3 targets that were clearly associated with OC: XXYLT1, TNFSF8, and HSD17B14. The most compelling of these was TNFSF8, as it passed all multiple validations and was categorized as a Tier 1 target. In contrast, although XXYLT1 and HSD17B14 failed some analyses, they passed the majority of validations and had moderate co‐localization results, showing moderate evidentiality, and were therefore classified as Tier 2. Notably, in this study, we identified several factors such as smoking and alcohol intake as risk factors for OC and found that changes in specific immune cells and daily cigarette use can influence the risk of OC by affecting the aforementioned proteins. Finally, the druggability assessment identified these three proteins as potential therapeutic targets; however, drugs related to TNFSF8 and XXYLT1 may pose some side effects. Given the findings, these three plasma proteins should be considered as potential therapeutic targets for OC.

Recently, Tian et al. published an MR‐based study identifying potential therapeutic targets for oral cancer; however, their prioritized genes (e.g., CCDC167, MID2) differ from those identified in our study (TNFSF8, XXYLT1, HSD17B14) [26]. This discrepancy can be attributed to several key differences in data sources and analytical strategies. First, while Tian et al. primarily utilized expression QTLs (eQTLs) from blood sources, our study integrated protein QTLs (pQTLs) from the deCODE cohort with eQTL evidence, prioritizing targets at the protein level, which may better reflect functional relevance. Second, our multi‐omics framework incorporated colocalization (posterior probability > 0.7) and HEIDI tests to exclude horizontal pleiotropy, whereas Tian et al. employed a different set of validation criteria. Third, the two studies used overlapping but not identical GWAS summary statistics, with differences in sample composition and imputation methods potentially contributing to divergent findings.

CD30 ligand (CD30L, TNFSF8) belongs to the tumour necrosis factor (TNF) superfamily. It is expressed by a range of normal cells, including mast cells, activated T lymphocytes, and granulocytes [27]. In contrast, under pathological conditions, CD30L expression has been linked to malignant lymphoid disorders such as Hodgkin's lymphoma and leukaemia [27, 28]. While there are limited reports on the relationship between CD30L and OC, some studies suggest a close association of CD30L with other types of tumours, including ovarian and basal cell carcinomas [29, 30]. Given the similarity between basal cell carcinomas originating from epidermal cells and the squamous epithelial cells involved in OC, it is plausible to hypothesize that CD30L may play a comparable role in the carcinogenesis and progression of OC. Our further analysis revealed that CD8+ T cell percentage leukocytes promote OC by enhancing TNFSF8 (CD30L) expression. This aligns with previous findings that CD30L is highly expressed in activated T lymphocytes [27], corroborating the results of this study. Moreover, Zhang et *al*. reported that increased CD8+ T cell density is associated with proliferative verrucous leukoplakia, a precancerous lesion of OSCC that has the potential to develop into oral squamous cell carcinoma (OSCC) [31]. Another study demonstrated that CD8+ T cells are positively correlated with lymphatic metastasis in OSCC [32], suggesting that CD8+ T cells may facilitate the onset and progression of OC. Notably, no studies have directly investigated TNFSF8 in OC, highlighting the novelty of our findings.

Therapeutically, TNFSF8 represents a tractable target due to existing biologics such as Brentuximab Vedotin, an FDA‐approved antibody‐drug conjugate targeting CD30 in lymphoma [33]; However, our PHEWAS‐MR analysis highlights systemic TNFSF8 inhibition as a potential risk factor for skin cancer (FDR‐*p* < 0.05), underscoring the need for precision‐driven strategies. Compared to established immune checkpoints like PD‐1/PD‐L1, the TNFSF8‐CD30 axis offers a novel therapeutic avenue in oral cancer pathogenesis. While PD‐1 inhibitors exhibit limited efficacy in OC (objective response rates < 20%) [34], TNFSF8 modulation enhances anti‐tumour immunity through dual mechanisms: (1) lowering CD8+ T‐cell activation thresholds via CD30 signalling [27] and (2) augmenting co‐stimulatory functions of antigen‐presenting cells to prime T‐cell responses [27]. Preclinical evidence from lymphoma models demonstrates that combining CD30‐targeted agents (e.g., Brentuximab Vedotin) with PD‐1 inhibitors synergistically improves tumour clearance [33]. Translating this approach to OC, localized delivery of anti‐TNFSF8 antibodies (e.g., intratumoral administration) could minimize systemic toxicity while addressing PD‐1 resistance—a critical challenge in tumours with coexisting immunosuppressive signals (e.g., CTLA‐4, LAG‐3) [35]. This strategy not only circumvents the skin cancer risks identified in our analysis but also leverages TNFSF8 role in immune activation to overcome microenvironmental immunosuppression. By integrating targeted TNFSF8 blockade with PD‐1 inhibition, our findings advocate for a combinatorial regimen that amplifies anti‐tumour immunity while prioritizing patient safety through precision immunotherapy design.

Our findings establish that sustained tobacco exposure significantly upregulates TNFSF8 expression, thereby accelerating OC pathogenesis. Despite its mechanistic significance, the interplay between smoking and TNFSF8 in OC remains poorly characterized in existing literature. In parallel, emerging multi‐omics evidence reveals that tobacco smoking induces genome‐wide DNA methylation, a process implicated in cardiovascular and neoplastic disease progression [36]. This epigenetic paradigm shift necessitates focused investigation into smoking‐associated TNFSF8 methylation dynamics to delineate its etiological contributions to OC. Critically, our data advocate for immediate translation of evidence‐based preventive measures—particularly tobacco cessation programs—to attenuate TNFSF8‐driven malignancy and reduce OC incidence at the population level.

Currently, the existing literature on xyloside xylosyltransferase 1 (XXYLT1) and its implications for cancer is limited. XXYLT1, a member of the xylosyltransferases family, impacts the O‐glucosylated EGF repeats of Notch [37]. Notch signalling is known to be involved in various cancers, including leukaemia, where pathological mutations can promote oncogenesis [38]. Studies have suggested that activation of the Notch pathway in leukaemia increases the expression of XXYLT1 mRNA [39], highlighting a potential oncogenic role for XXYLT1 through its interaction with this pathway. Additionally, research has suggested that the long non‐coding RNA (lncRNA) XXYLT1‐AS2 may exacerbate tumour progression by mediating autophagy inhibition [40], indirectly suggesting a link between XXYLT1 and cancer development.

17β‐hydroxysteroid dehydrogenase type 14 (HSD17B14) is an enzyme involved in steroid metabolism, converting estradiol to estrone and androstenediol to dehydroepiandrosterone [41]. In our study of risk factor analysis, a causal relationship was identified between androgen levels and OC, where androgens have previously been shown to inhibit the development of head and neck cancers [42]. This evidence supports the hypothesis that HSD17B14 plays a significant role in OC development. Additionally, we discovered that CD4 on HLA DR+ CD4+ T cells plays a mediating role in the carcinogenesis of OC induced by HSD17B14. However, this aspect of our findings has not yet been corroborated by other studies. Given this gap in the existing literature, further experimental validation is necessary to confirm these results.

To further validate the reliability of our bioinformatics analysis, we conducted quantitative real‐time PCR. The results showed that XXYLT1 and HSD17B14 were significantly upregulated in OSCC cell lines compared to normal cells, validating their predicted role as oncogenic hubs in OSCC. However, although bioinformatics analysis indicated significant upregulation of TNFSF8 in OSCC tissues, its expression was not consistently detected in the cell lines. As a signalling molecule, TNFSF8 may function through ligand‐receptor interactions that are effective at low concentrations. Therefore, its low or undetectable expression in unstimulated monoculture systems in vitro does not preclude its biological relevance as a hub gene. This highlights the functional heterogeneity among hub genes. While XXYLT1 and HSD17B14 are amenable to study in standard culture systems, TNFSF8 appears to function as a low‐abundance, inducible signalling molecule whose expression is likely context‐dependent and restricted to specific cell types or activation states within the tumour microenvironment. This finding does not diminish its potential importance; rather, it suggests that TNFSF8 may contribute to OSCC pathogenesis not via intrinsic expression within cancer cells, but through dynamic interactions with the immune microenvironment.

Our study presents several distinct advantages, outlined as follows: First, Our investigation employed a multi‐omics framework to systematically analyse plasma proteins with causal associations with OC, leveraging the largest available OC GWAS dataset integrated with eQTL and pQTL data. Second, Identification of genetically determined risk factors (e.g., Smoking and alcohol intake.) for OC through MR, revealing mechanistic contributions of Smoking and immune cell dynamics (e.g., CD8+ T cell proportion among leukocytes) to OC pathogenesis via plasma protein modulation. Furthermore, there is a significant research gap concerning the roles of TNFSF8, XXYLT1, and HSD17B14 in OC. This study makes a valuable contribution towards bridging this gap. Third, we conducted drug prediction and side effect prediction analyses, providing an important reference for future research efforts. Importantly, the convergence of our MR predictions with qPCR validation in OSCC cell lines for XXYLT1 and HSD17B14 provides orthogonal evidence supporting the reliability of our findings.

Despite the valuable contributions of this study, several limitations should be noted. First, all data for this MR analysis were derived from individuals of European ancestry, thus not reflecting the genetic diversity of global populations. This limitation suggests the need for future research to incorporate more diverse ancestry groups. Second, our study did not address the variations in OC pathology stages, which could have provided further insights into disease mechanisms. Future efforts should aim to include this aspect in the analysis. Third, notably, none of the prioritized candidate targets in this study met the conventional colocalization significance threshold (posterior probability > 0.7). While TNFSF8, classified as a Tier 1 candidate, demonstrated the strongest association with a maximum posterior probability of 0.66, we retained its significance designation based on the recognized limitations of current colocalization methodologies, which exhibit an estimated 60% false‐negative rate [43]. This conservative interpretation aligns with recommendations for accounting for methodological uncertainty in causal inference studies. Fourth, although we conducted qPCR validation in OSCC cell lines to support our bioinformatics predictions, the experimental component of this study is limited in scope. This validation was restricted to mRNA‐level analysis across a limited panel of cell lines and did not include protein‐level confirmation or functional assays. Therefore, the specific roles of XXYLT1, HSD17B14, and TNFSF8 in oral carcinogenesis require further investigation. Furthermore, validating these findings in larger clinical cohorts via methods such as immunohistochemistry is essential to confirm protein‐level expression patterns, clinical significance, and correlations with patient prognosis, which would ultimately enhance the translational relevance of our results.

## 5 Conclusions

Multi‐omics MR prioritizes TNFSF8 as a therapeutic target for oral cancer. qPCR validation confirms the dysregulation of XXYLT1 and HSD17B14, while findings regarding TNFSF8 underscore its immune origin. These findings warrant further functional studies.

## Author Contributions

**Meishan Huang:** conceptualization (equal), formal analysis (equal), methodology (equal), validation (equal), writing – original draft (equal), writing – review and editing (equal). **Hongwei Wang:** conceptualization (equal), formal analysis (equal), methodology (equal), writing – original draft (equal). **Xiaoqiang Mo:** conceptualization (equal), formal analysis (equal), methodology (equal), writing – original draft (equal). **Xiong Huang:** conceptualization (equal), formal analysis (equal), methodology (equal), writing – original draft (equal). **Tao Liang:** software (equal), validation (equal), visualization (equal), writing – original draft (equal). **Xiuwen Mo:** software (equal), validation (equal), visualization (equal), writing – original draft (equal). **Minsi Li:** formal analysis (equal). **Rong Fu:** software (equal), validation (equal), visualization (equal), writing – original draft (equal). **Xidai Long:** methodology (equal), project administration (equal), supervision (equal), writing – review and editing (equal). **Xuanping Huang:** conceptualization (equal), methodology (equal), project administration (equal), resources (equal), supervision (equal), writing – review and editing (equal).

## Funding

The authors have nothing to report.

## Ethics Statement

All data in this article was obtained from public databases. All cell lines used in this study (SCC‐9, SCC‐25, and HOEC) were obtained from commercial sources and as such, there are no relevant statements of ethical approval, consent to participate, etc.

## Consent

The authors have nothing to report.

## Conflicts of Interest

The authors declare no conflicts of interest.

## Supporting information


**Figure S1:** PPI network diagram of 3 plasma proteins.
**Figure S2:** Plot of GO enrichment analysis of TNFSF8.
**Figure S3:** Plot of KEGG enrichment analysis of TNFSF8.
**Figure S4:** Plot of GO enrichment analysis of HSD17B14.
**Figure S5:** Plot of KEGG enrichment analysis of HSD17B14.
**Figure S6:** Plot of GO enrichment analysis of XXYLT1.
**Figure S7:** Plot of KEGG enrichment analysis of XXYLT1.
**Table S1:** Characteristics of the GWAS summary data.
**Table S2:** Genetic instruments for plasma proteins.
**Table S3:** Results of MR analysis of plasma proteins and OC.
**Table S4:** Results of MR analysis of OC and plasma proteins.
**Table S5:** SMR and HEIDI results of 8 plasma proteins and OC.
**Table S6:** Colocalization results of 8 significant associated proteins with OC.
**Table S7:** Results of SMR, Heidi tests and MR for eQTLs and OC.
**Table S8:** The MR results between variable risk factors and oral cancer.
**Table S9:** Results of mediation analysis from gene expression to OC risk via protein levels.
**Table S10:** Results of Mendelian randomization analysis of other immune cells and oral cancer (not positive).
**Table S11:** Results of heterogeneity and pleiotropy analysis of variable risk factors and oral cancer.
**Table S12:** Results of mediation analysis from protein levels to OC via immune cells.
**Table S13:** PEHWAS‐MR results for 3 plasma proteins (positive results).

## Data Availability

The data in this paper are taken from public databases and can be obtained from the corresponding references in the text, or from the GWAS IDs and download sites in Supporting Information.

## References

[1] T. Sasahira and T. Kirita , “Hallmarks of Cancer‐Related Newly Prognostic Factors of Oral Squamous Cell Carcinoma,” International Journal of Molecular Sciences 19, no. 8 (2018): 2413. 30115834 10.3390/ijms19082413 PMC6121568

[2] R. L. Siegel , A. N. Giaquinto , and A. Jemal , “Cancer Statistics, 2024,” CA: A Cancer Journal for Clinicians 74, no. 1 (2024): 12–49. 38230766 10.3322/caac.21820

[3] H. V. Worthington , V. M. Bulsara , A. M. Glenny , J. E. Clarkson , D. I. Conway , and M. Macluskey , “Interventions for the Treatment of Oral Cavity and Oropharyngeal Cancers: Surgical Treatment,” Cochrane Database of Systematic Reviews 8, no. 8 (2023): Cd006205. 37650478 10.1002/14651858.CD006205.pub5 PMC10476948

[4] M. Zhang , J. Liang , Y. Yang , H. Liang , H. Jia , and D. Li , “Current Trends of Targeted Drug Delivery for Oral Cancer Therapy,” Frontiers in Bioengineering and Biotechnology 8 (2020): 618931. 33425881 10.3389/fbioe.2020.618931 PMC7793972

[5] Q. Wang , Q. Shi , Z. Wang , J. Lu , and J. Hou , “Integrating Plasma Proteomes With Genome‐Wide Association Data for Causal Protein Identification in Multiple Myeloma,” BMC Medicine 21, no. 1 (2023): 377. 37775746 10.1186/s12916-023-03086-0 PMC10542236

[6] X. W. Cai , W. W. Yu , W. Yu , et al., “Tissue‐Based Quantitative Proteomics to Screen and Identify the Potential Biomarkers for Early Recurrence/Metastasis of Esophageal Squamous Cell Carcinoma,” Cancer Medicine 7, no. 6 (2018): 2504–2517. 29683265 10.1002/cam4.1463 PMC6010861

[7] Z. Wu , J. B. Doondeea , A. M. Gholami , et al., “Quantitative Chemical Proteomics Reveals New Potential Drug Targets in Head and Neck Cancer,” Molecular & Cellular Proteomics 10, no. 12 (2011): M111.011635. 10.1074/mcp.M111.011635 PMC3237086 21955398

[8] T. Y. Kim , K. S. Koh , J. M. Ju , et al., “Proteomics Analysis of Antitumor Activity of Agrimonia Pilosa Ledeb. In Human Oral Squamous Cell Carcinoma Cells,” Current Issues in Molecular Biology 44, no. 8 (2022): 3324–3334. 35892715 10.3390/cimb44080229 PMC9332088

[9] J. Bowden and M. V. Holmes , “Meta‐Analysis and Mendelian Randomization: A Review,” Research Synthesis Methods 10, no. 4 (2019): 486–496. 30861319 10.1002/jrsm.1346 PMC6973275

[10] P. Sekula , M. F. Del Greco , C. Pattaro , and A. Köttgen , “Mendelian Randomization as an Approach to Assess Causality Using Observational Data,” Journal of the American Society of Nephrology 27, no. 11 (2016): 3253–3265. 27486138 10.1681/ASN.2016010098 PMC5084898

[11] C. Lesseur , B. Diergaarde , A. F. Olshan , et al., “Genome‐Wide Association Analyses Identify New Susceptibility Loci for Oral Cavity and Pharyngeal Cancer,” Nature Genetics 48, no. 12 (2016): 1544–1550. 27749845 10.1038/ng.3685 PMC5131845

[12] E. Ferkingstad , P. Sulem , B. A. Atlason , et al., “Large‐Scale Integration of the Plasma Proteome With Genetics and Disease,” Nature Genetics 53, no. 12 (2021): 1712–1721. 34857953 10.1038/s41588-021-00978-w

[13] U. Võsa , A. Claringbould , H. J. Westra , et al., “Large‐Scale Cis‐ and Trans‐eQTL Analyses Identify Thousands of Genetic Loci and Polygenic Scores That Regulate Blood Gene Expression,” Nature Genetics 53, no. 9 (2021): 1300–1310. 34475573 10.1038/s41588-021-00913-z PMC8432599

[14] V. W. Skrivankova , R. C. Richmond , B. A. R. Woolf , et al., “Strengthening the Reporting of Observational Studies in Epidemiology Using Mendelian Randomization: The STROBE‐MR Statement,” JAMA 326, no. 16 (2021): 1614–1621. 34698778 10.1001/jama.2021.18236

[15] E. Sanderson , M. M. Glymour , M. V. Holmes , et al., “Mendelian randomization,” Nature Reviews Methods Primers 2 (2022): 6. 10.1038/s43586-021-00092-5 PMC7614635 37325194

[16] Y. Wu , J. Zeng , F. Zhang , et al., “Integrative Analysis of Omics Summary Data Reveals Putative Mechanisms Underlying Complex Traits,” Nature Communications 9, no. 1 (2018): 918. 10.1038/s41467-018-03371-0 PMC5834629 29500431

[17] T. Islam , M. R. Rahman , A. Khan , and M. A. Moni , “Integration of Mendelian Randomisation and Systems Biology Models to Identify Novel Blood‐Based Biomarkers for Stroke,” Journal of Biomedical Informatics 141 (2023): 104345. 36958462 10.1016/j.jbi.2023.104345

[18] J. Bowden , M. F. Del Greco , C. Minelli , et al., “Improving the Accuracy of Two‐Sample Summary‐Data Mendelian Randomization: Moving Beyond the NOME Assumption,” International Journal of Epidemiology 48, no. 3 (2019): 728–742. 30561657 10.1093/ije/dyy258 PMC6659376

[19] C. Ellervik , S. Mora , A. Kuś , et al., “Effects of Thyroid Function on Hemostasis, Coagulation, and Fibrinolysis: A Mendelian Randomization Study,” Thyroid 31, no. 9 (2021): 1305–1315. 34210154 10.1089/thy.2021.0055 PMC8558080

[20] C. Giambartolomei , D. Vukcevic , E. E. Schadt , et al., “Bayesian Test for Colocalisation Between Pairs of Genetic Association Studies Using Summary Statistics,” PLoS Genetics 10, no. 5 (2014): e1004383. 24830394 10.1371/journal.pgen.1004383 PMC4022491

[21] J. Chen , X. Ruan , Y. Sun , et al., “Multi‐Omic Insight Into the Molecular Networks of Mitochondrial Dysfunction in the Pathogenesis of Inflammatory Bowel Disease,” eBioMedicine 99 (2024): 104934. 38103512 10.1016/j.ebiom.2023.104934 PMC10765009

[22] A. R. Carter , E. Sanderson , G. Hammerton , et al., “Mendelian Randomisation for Mediation Analysis: Current Methods and Challenges for Implementation,” European Journal of Epidemiology 36, no. 5 (2021): 465–478. 33961203 10.1007/s10654-021-00757-1 PMC8159796

[23] D. Mendez , A. Gaulton , A. P. Bento , et al., “ChEMBL: Towards Direct Deposition of Bioassay Data,” Nucleic Acids Research 47, no. D1 (2019): D930–D940. 30398643 10.1093/nar/gky1075 PMC6323927

[24] S. L. Freshour , S. Kiwala , K. C. Cotto , et al., “Integration of the Drug‐Gene Interaction Database (DGIdb 4.0) With Open Crowdsource Efforts,” Nucleic Acids Research 49, no. D1 (2021): D1144–D1151. 33237278 10.1093/nar/gkaa1084 PMC7778926

[25] D. S. Wishart , Y. D. Feunang , A. C. Guo , et al., “DrugBank 5.0: A Major Update to the DrugBank Database for 2018,” Nucleic Acids Research 46, no. D1 (2018): D1074–D1082. 29126136 10.1093/nar/gkx1037 PMC5753335

[26] T. Tian , S. Li , H. Shaonan , et al., “Causal Inference of the Effect of Plasma Proteins on the Incidence of Oral Cancer: Two‐Sample Mendelian Randomization,” BMC Oral Health 24, no. 1 (2024): 1049. 39245738 10.1186/s12903-024-04837-y PMC11382374

[27] R. Horie and T. Watanabe , “CD30: Expression and Function in Health and Disease,” Seminars in Immunology 10, no. 6 (1998): 457–470. 9826579 10.1006/smim.1998.0156

[28] H. J. Gruss , D. Ulrich , S. Braddy , R. J. Armitage , and S. K. Dower , “Recombinant CD30 Ligand and CD40 Ligand Share Common Biological Activities on Hodgkin and Reed‐Sternberg Cells,” European Journal of Immunology 25, no. 7 (1995): 2083–2089. 7621881 10.1002/eji.1830250742

[29] A. Mielczarek‐Palacz , J. Sikora , Z. Kondera‐Anasz , and G. Hauza , “Imbalance in Serum Soluble CD30/CD30L and CD40/CD40L Systems Are Associated With Ovarian Tumors,” Human Immunology 74, no. 1 (2013): 70–74. 23073297 10.1016/j.humimm.2012.10.004

[30] N. C. Diaconu , R. Kaminska , A. Naukkarinen , R. J. Harvima , G. Nilsson , and I. T. Harvima , “Increase in CD30 Ligand/CD153 and TNF‐Alpha Expressing Mast Cells in Basal Cell Carcinoma,” Cancer Immunology, Immunotherapy 56, no. 9 (2007): 1407–1415. 17268792 10.1007/s00262-007-0290-7 PMC11030587

[31] D. Fernandes , C. O. Barbeiro , M. P. Palaçon , et al., “High Density of CD8 T Cell and Immune Imbalance of T Lymphocytes Subsets Are Associated With Proliferative Verrucous Leukoplakia,” Immunology 168, no. 1 (2023): 96–109. 36056642 10.1111/imm.13565

[32] Y. Zhang , J. Wang , J. Yu , and H. Zhu , “FKBP4 Correlates With CD8(+) T Cells and Lymphatic Metastases in Oral Squamous Cell Carcinoma,” Oral Diseases 30, no. 2 (2024): 422–432. 36067001 10.1111/odi.14371

[33] A. Younes , N. L. Bartlett , J. P. Leonard , et al., “Brentuximab Vedotin (SGN‐35) for Relapsed CD30‐Positive Lymphomas,” New England Journal of Medicine 363, no. 19 (2010): 1812–1821. 21047225 10.1056/NEJMoa1002965

[34] R. L. Ferris , G. Blumenschein, Jr. , J. Fayette , et al., “Nivolumab for Recurrent Squamous‐Cell Carcinoma of the Head and Neck,” New England Journal of Medicine 375, no. 19 (2016): 1856–1867. 27718784 10.1056/NEJMoa1602252 PMC5564292

[35] P. Sharma and J. P. Allison , “The Future of Immune Checkpoint Therapy,” Science 348, no. 6230 (2015): 56–61. 25838373 10.1126/science.aaa8172

[36] W. Li , P. Dong , Y. Li , et al., “Examining the Potential Causal Relationships Among Smoking Behaviours, Blood DNA Methylation Profiles, and the Development of Coronary Heart Disease and Myocardial Infarction,” Clinical Epigenetics 16, no. 1 (2024): 173. 39614281 10.1186/s13148-024-01791-y PMC11606085

[37] M. K. Sethi , F. F. Buettner , A. Ashikov , and H. Bakker , “In Vitro Assays of Orphan Glycosyltransferases and Their Application to Identify Notch Xylosyltransferases,” Methods in Molecular Biology 1022 (2013): 307–320. 23765671 10.1007/978-1-62703-465-4_23

[38] C. Lobry , P. Oh , M. R. Mansour , A. T. Look , and I. Aifantis , “Notch Signaling: Switching an Oncogene to a Tumor Suppressor,” Blood 123, no. 16 (2014): 2451–2459. 24608975 10.1182/blood-2013-08-355818 PMC3990910

[39] S. Wang , M. Itoh , E. Shiratori , M. Ohtaka , and S. Tohda , “NOTCH Activation Promotes Glycosyltransferase Expression in Human Myeloid Leukemia Cells,” Hematology Reports 10, no. 3 (2018): 7576. 30344988 10.4081/hr.2018.7576 PMC6176398

[40] X. Li , Y. Wu , P. Wang , et al., “LncRNA XXYLT1‐AS2 Promotes Tumor Progression via Autophagy Inhibition Through Ubiquitinated Degradation of TFEB in Hepatocellular Carcinoma,” Clinical & Translational Oncology 26, no. 3 (2024): 698–708. 37540409 10.1007/s12094-023-03294-3

[41] E. Hilborn , O. Stål , and A. Jansson , “Estrogen and Androgen‐Converting Enzymes 17β‐Hydroxysteroid Dehydrogenase and Their Involvement in Cancer: With a Special Focus on 17β‐Hydroxysteroid Dehydrogenase Type 1, 2, and Breast Cancer,” Oncotarget 8, no. 18 (2017): 30552–30562. 28430630 10.18632/oncotarget.15547 PMC5444764

[42] D. Manestar , G. Malvic , M. Velepic , et al., “Perioperative Substitution Testosterone Therapy in Patients With Advanced Head and Neck Squamous Cell Carcinoma,” Critical Reviews in Oncology/Hematology 188 (2023): 104062. 37385306 10.1016/j.critrevonc.2023.104062

[43] L. Chen , J. E. Peters , B. Prins , et al., “Systematic Mendelian Randomization Using the Human Plasma Proteome to Discover Potential Therapeutic Targets for Stroke,” Nature Communications 13, no. 1 (2022): 6143. 10.1038/s41467-022-33675-1 PMC9576777 36253349

