# Supplementary material for: Multi‐Omics Profiling Identifies Novel Therapeutic Targets and Pathobiological Mechanisms in Oral Cancer
Source: J Cell Mol Med. 2026 Aug 4;30(15):e71272. doi: 10.1111/jcmm.71272 (PMC13434842; doi:10.1111/jcmm.71272)
Supplement: Supplementary file 1 [file JCMM-30-e71272-s001.docx]

***Manuscript title***

**Multi-omics Profiling Identifies Novel Therapeutic Targets and Pathobiological Mechanisms in Oral Cancer**

***Authors***

Meishan Huang(First-author), Ph.D 1,2;#;hms192285@163.com

Hongwei Wang (Co-first author), MD.Sc 3,5,6;#; wonghongwei@126.com

Xiaoqiang Mo(Co-author), Ph.D 4,5;29109799@qq.com

Xiong Huang (Co-author), MD.Sc 5,6; 2426759260@qq.com

Tao Liang (Co-author), MD.Sc 5,6; [2290079378@qq.com](mailto:2290079378@qq.com)

Xiuwen Mo (Co-author), MD.Sc 5,6; [2474879861@qq.com](mailto:2474879861@qq.com)

Minsi Li(Co-author), Ph.D 1,2; 1246760240@qq.com

Rong Fu (Co-author), MD.Sc 5,6; 2450206685@qq.com

Xidai Long(Co-corresponding author), Ph.D 4,6;*; [sjtulongxd@263.net](mailto:sjtulongxd@263.net)

Xuanping Huang(First-Corresponding author), Ph.D 1,2;*; hxp120@126.com

*Corresponding authors.

#These authors contributed equally to this study.

1 College & Hospital of Stomatology, Guangxi Medical University, Nanning 530021, China, yfzxb@gxmu.edu.cn.

2 Guangxi Key Laboratory of Oral and Maxillofacial Rehabilitation and Reconstruction, Nanning 530021, China, yfzxb@gxmu.edu.cn.

3 Department of Stomatology, Beihai Hospital of Chinese Medicine, Beihai Hospital Affiliated To Guangxi university of Chinese Medicine. Beihai, Guangxi 536000, China, bhszy@126.com.

4 Medical College of Guangxi University, Nanning, Guangxi 530000, China, yxybgs@gxu.edu.cn.

5 Youjiang Medical University for Nationalities, No. 98 Countryside Road, Baise, Guangxi 533000, China, ymun@ymun.edu.cn.

6 Key Laboratory of Tumor Molecular Pathology of Baise, Baise, Guangxi 533000, China, ymun@ymun.edu.cn.

***Name:*** Xuanping Huang;Xidai Long

***Email:*** hxp120@126.com; sjtulongxd@263.net.

***Address:*** College & Hospital of Stomatology, Guangxi Medical University, Nanning 530021, China;

Medical College of Guangxi University, Nanning, Guangxi 530000, China.

***institutional email:*** yfzxb@gxmu.edu.cn , yxybgs@gxu.edu.cn.

Appendix Figure 1. PPI network diagram of 3 plasma proteins

The connecting lines in the PPI network diagram indicate linkages between the proteins. Notably, TNFSF8 is primarily associated with inflammatory factors such as TNF, however, there is no obvious interconnection between these targets.

Appendix Figure 2. Plot of GO enrichment analysis of TNFSF8

In gene ontology analysis, 'BP' represents Biological Process, 'CC' refers to Cellular Component, and 'MF' denotes Molecular Function. For visual representation, the first six components of each category were selected for plotting. The P-values used in the analysis were FDR-corrected, with darker colors on the plot indicating smaller P-values, thus highlighting more statistically significant associations.

Appendix Figure 3. Plot of KEGG enrichment analysis of TNFSF8

In Kyoto Encyclopedia of Genes and Genomes analysis, 'BP' represents Biological Process, 'CC' refers to Cellular Component, and 'MF' denotes Molecular Function. For visual representation, the first six components of each category were selected for plotting. The P-values used in the analysis were FDR-corrected, with darker colors on the plot indicating smaller P-values, thus highlighting more statistically significant associations.

Appendix Figure 4. Plot of GO enrichment analysis of HSD17B14

In gene ontology analysis, 'BP' represents Biological Process, 'CC' refers to Cellular Component, and 'MF' denotes Molecular Function. For visual representation, the first six components of each category were selected for plotting. The P-values used in the analysis were FDR-corrected, with darker colors on the plot indicating smaller P-values, thus highlighting more statistically significant associations.

Appendix Figure 5. Plot of KEGG enrichment analysis of HSD17B14

In Kyoto Encyclopedia of Genes and Genomes analysis, 'BP' represents Biological Process, 'CC' refers to Cellular Component, and 'MF' denotes Molecular Function. For visual representation, the first six components of each category were selected for plotting. The P-values used in the analysis were FDR-corrected, with darker colors on the plot indicating smaller P-values, thus highlighting more statistically significant associations.

Appendix Figure 6. Plot of GO enrichment analysis of XXYLT1

In gene ontology analysis, 'BP' represents Biological Process, 'CC' refers to Cellular Component, and 'MF' denotes Molecular Function. For visual representation, the first six components of each category were selected for plotting. The P-values used in the analysis were FDR-corrected, with darker colors on the plot indicating smaller P-values, thus highlighting more statistically significant associations.

Appendix Figure 7. Plot of KEGG enrichment analysis of XXYLT1

In Kyoto Encyclopedia of Genes and Genomes analysis, 'BP' represents Biological Process, 'CC' refers to Cellular Component, and 'MF' denotes Molecular Function. For visual representation, the first six components of each category were selected for plotting. The P-values used in the analysis were FDR-corrected, with darker colors on the plot indicating smaller P-values.Although the p-values depicted in the figure do not fall below the 0.05 threshold after FDR correction, the results presented still highlight the top six items that can be enriched in each category (BP, CC, MF). These items are included because they represent the most significantly enriched terms within each factor, providing valuable insights despite not meeting the conventional threshold for statistical significance.

| **Appendix Table 1. Characteristics of the GWAS summary data** | | | | |  |
| --- | --- | --- | --- | --- | --- |
| Characteristic | Sample size | Consortium | descents | PMID | Download Site or GWAS ID |
| Oral cancer | 372,373 | UK Biobank | European | NA | <https://gwas.mrcieu.ac.uk/datasets/ieu-b-4961/> |
|  | 477,183 | FinnGen R12 | European | 36653562 | <https://www.finngen.fi/en/access_results> |
|  | 7615 | GAME-ON | European | 27749845 | <https://pubmed.ncbi.nlm.nih.gov/27749845/> |
| Plasma Proteins |  |  |  |  |  |
| pQTL | 35,559 | deCODE Genetics | European | 34857953 | <http://www.decode.com/summarydata/> |
| pQTL | 54,219 | The UK Biobank Pharma Proteomics Project | European | 37794186 | <https://metabolomips.org/ukbbpgwas/> |
| Blood-Derived Expression |  |  |  |  |  |
| eQTL | 31,684 | eQTLGen Consortium | European | 34475573 | <https://eqtlgen.org/> |
| Anthropometric traits |  |  |  |  |  |
| BMI | 339,224 | NA | European | 25673413 | NA |
| HIP | 694,649 | NA | European | 30239722 | NA |
| HIP(adjBMI) | 694,649 | NA | European | 30239722 | NA |
| WC(adjBMI) | 694,649 | NA | European | 30239722 | NA |
| WHR | 694,649 | NA | European | 30239722 | NA |
| WHR(adjBMI) | 694,649 | NA | European | 30239722 | NA |
| Basal metabolic rate | 454,874 | MRC-IEU | European | NA | ukb-b-16446 |
| Arm fat mass (left) | 454,684 | MRC-IEU | European | NA | ukb-b-8338 |
| Body fat percentage | 454,633 | MRC-IEU | European | NA | ukb-b-8909 |
| Waist circumference | 462,166 | MRC-IEU | European | NA | ukb-b-9405 |
| Whole body fat mass | 454,137 | MRC-IEU | European | NA | ukb-b-19393 |
| Trunk fat mass | 454,588 | MRC-IEU | European | NA | [ukb-b-20044](https://gwas.mrcieu.ac.uk/datasets/ukb-b-20044/) |
| Leg fat mass (left) | 454,823 | MRC-IEU | European | NA | [ukb-b-7212](https://gwas.mrcieu.ac.uk/datasets/ukb-b-7212/) |
| diastolic blood pressure | 757,601 | International Consortium of Blood Pressure | European | 30224653 | [ieu-b-39](https://gwas.mrcieu.ac.uk/datasets/ieu-b-39/) |
| Overall health rating | 460,844 | MRC-IEU | European | NA | [ukb-b-6306](https://gwas.mrcieu.ac.uk/datasets/ukb-b-6306/) |
| Lifestyle factors |  |  |  |  |  |
| Coffee consumption | 370,000 | NA | European | 31046077 | NA |
| Bitter alcoholic beverages | 370,000 | NA | European | 31046077 | NA |
| Tea consumption | 370,000 | NA | European | 31046077 | NA |
| Age at first sexual intercourse | 397,338 | NA | European | 34211149 | ebi-a-GCST90000047 |
| Lifetime number of sexual partners | 378,882 | MRC-IEU | European | NA | ebi-a-GCST90000047 |
| Alcohol intake versus 10 years previously | 313,248 | Neale Lab | European | NA | ukb-a-32 |
| Smoking Cessation | 547,219 | GWAS and Sequencing Consortium of Alcohol and Nicotine use | European | 30643251 | NA |
| Age of Initiation of Regular Smoking | 341,427 | GWAS and Sequencing Consortium of Alcohol and Nicotine use | European | 30643251 | NA |
| Smoking Initiation | 1,232,091 | GWAS and Sequencing Consortium of Alcohol and Nicotine use | European | 30643251 | NA |
| Cigarettes per Day | 337,334 | GWAS and Sequencing Consortium of Alcohol and Nicotine use | European | 30643251 | ieu-b-25 |
| Smoking status: Never | 359,706 | Neale lab | European | 30643251 | ukb-d-20116_0 |
| Current tobacco smoking | 337,030 | Neale Lab | European | NA | ukb-a-16 |
| Drinks per Week | 941,280 | GWAS and Sequencing Consortium of Alcohol and Nicotine use | European | 30643251 | NA |
| Daytime nap | 452,633 | NA | European | 33568662 | ebi-a-GCST011494 |
| insomnia | 462,341 | MRC-IEU | European | NA | ukb-b-3957 |
| Time spent doing light physical activity | 64,949 | MRC-IEU | European | NA | ukb-b-8865 |
| Time spent driving | 310,555 | MRC-IEU | European | NA | ukb-b-3793 |
| Time spent using computer | 360,895 | MRC-IEU | European | NA | ukb-b-4522 |
| Time spent watching television (TV) | 437,887 | MRC-IEU | European | NA | ukb-b-5192 |
| Education and family factors |  |  |  |  |  |
| Years of schooling | 99,970 | Within family GWAS consortium | European | NA | ieu-a-1239 |
| Subjective well being | 298,420 | SSGAC | European | 27,089,181 | ieu-a-1009 |
| Average total household income before tax | 397,751 | MRC-IEU | European | NA | ukb-b-7408 |
| College or University degree | 458,079 | MRC-IEU | European | NA | ukb-b-16489 |
| Cardiovascular disease |  |  |  |  |  |
| hypertension | 463,010 | MRC-IEU | European | NA | ukb-b-12493 |
| Coronary heart disease | 86,995 | NA | European | 21,378,990 | ieu-a-9 |
| Coronary artery disease | 296,525 | NA | European | 29,212,778 | ebi-a-GCST005195 |
| Atrial fibrillation | 1,030,836 | NA | European | 30,061,737 | ebi-a-GCST006414 |
| Heart failure | 977,323 | NA | European | 31,919,418 | ebi-a-GCST009541 |
| Stroke | 446,696 | NA | European | 29,531,354 | ebi-a-GCST90038613 |
| Immune system |  |  |  |  |  |
| 731 immune cells | 3,668 | NA | European | 32929287 | <https://pubmed.ncbi.nlm.nih.gov/32929287/> |
| T2D and Related Trait |  |  |  |  |  |
| Fasting glucose | 200,622 | NA | European | 34059833 | ebi-a-GCST90002232 |
| Fasting insulin | 151,013 | NA | European | 34059833 | ebi-a-GCST90002238 |
| HbA1c levels | 389,889 | NA | European | 34017140 | ebi-a-GCST90014006 |
| Type 2 diabetes | 1,114,458 | NA | European | 32541925 | NA |
| Serum metabolite |  |  |  |  |  |
| Alanine | 115,074 | NA | European | NA | met-d-Ala |
| Glutamine | 114,750 | NA | European | NA | met-d-Gln |
| Glycine | 114,972 | NA | European | NA | met-d-Gly |
| Histidine | 114,895 | NA | European | NA | met-d-His |
| Isoleucine | 115,075 | NA | European | NA | met-d-Ile |
| Leucine | 115,074 | NA | European | NA | met-d-Leu |
| 25-Hydroxyvitamin D levels | 496,946 | NA | European | 32242144 | ebi-a-GCST90000618 |
| Tyrosine | 114,911 | NA | European | NA | met-d-Tyr |
| Sex hormone |  |  |  |  |  |
| Testosterone levels | 353,805 | NA | European | 34017140 | ebi-a-GCST90014013 |
| Oestradiol levels | NA | Neale lab | European | NA | ukb-d-30800_raw |

**Appendix Table 2. Genetic Instruments for Plasma Proteins**

| deCODE |  |  |  |  |  |  |  |  |  |  |  |
| --- | --- | --- | --- | --- | --- | --- | --- | --- | --- | --- | --- |
| Gene names | SNP | Chr | Pos | Effect_allele | Other_allele | Beta | Se | Eaf | Samplesize | Pval | F-statistic |
| TNFSF8 | rs1006026 | chr9 | 114928990 | G | A | -0.14 | 0.01 | 0.46 | 35362 | 4.84E-63 | 280.82 |
| TNFSF8 | rs10125315 | chr9 | 114717229 | T | C | -0.05 | 0.01 | 0.25 | 35362 | 1.41E-08 | 32.18 |
| TNFSF8 | rs10817639 | chr9 | 114560932 | GTCTCAAAAG | GTCTCAAAAA | 0.08 | 0.01 | 0.09 | 35361 | 6.11E-09 | 33.80 |
| TNFSF8 | rs10982293 | chr9 | 114568046 | T | A | 0.07 | 0.01 | 0.13 | 35362 | 3.43E-09 | 34.92 |
| TNFSF8 | rs10982385 | chr9 | 114730737 | G | T | 0.07 | 0.01 | 0.40 | 35362 | 8.31E-16 | 64.79 |
| TNFSF8 | rs117303912 | chr9 | 114843981 | A | G | 0.13 | 0.02 | 0.04 | 35362 | 6.55E-09 | 33.66 |
| TNFSF8 | rs2418322 | chr9 | 114868457 | A | G | -0.05 | 0.01 | 0.40 | 35362 | 9.32E-09 | 32.98 |
| TNFSF8 | rs55859521 | chr9 | 114806680 | T | C | -0.13 | 0.02 | 0.03 | 35362 | 3.64E-08 | 30.33 |
| TNFSF8 | rs56404767 | chr9 | 114744681 | G | A | 0.12 | 0.02 | 0.04 | 35362 | 1.14E-08 | 32.59 |
| TNFSF8 | rs72756571 | chr9 | 114905608 | T | G | -0.12 | 0.01 | 0.10 | 35362 | 1.54E-20 | 86.31 |
| TNFSF8 | rs7853396 | chr9 | 114693299 | A | G | -0.06 | 0.01 | 0.43 | 35363 | 3.06E-13 | 53.16 |
| XXYLT1 | rs114044674 | chr3 | 195224752 | T | C | 0.29 | 0.02 | 0.05 | 35226 | 7.68E-57 | 252.40 |
| XXYLT1 | rs115363377 | chr3 | 195289265 | T | C | -0.13 | 0.01 | 0.08 | 35225 | 2.67E-17 | 71.57 |
| XXYLT1 | rs115549108 | chr3 | 195077799 | T | C | -0.16 | 0.02 | 0.04 | 35227 | 1.49E-14 | 59.11 |
| XXYLT1 | rs115717084 | chr3 | 195136324 | TCCGGCCCA | CCCGGCCCA | 0.19 | 0.02 | 0.04 | 35228 | 2.92E-19 | 80.49 |
| XXYLT1 | rs11707545 | chr3 | 194850607 | G | A | -0.11 | 0.02 | 0.05 | 35245 | 1.64E-08 | 31.88 |
| XXYLT1 | rs11915854 | chr3 | 195081925 | T | C | -0.10 | 0.02 | 0.07 | 35227 | 6.29E-10 | 38.23 |
| XXYLT1 | rs11917675 | chr3 | 195036853 | TACCAGATGGCGGGGGCAGGA | TACCAGATGGCGGGGGCAGGG | 0.06 | 0.01 | 0.21 | 35224 | 4.42E-09 | 34.43 |
| XXYLT1 | rs12695049 | chr3 | 195268211 | T | C | 0.20 | 0.01 | 0.47 | 35225 | 6.93E-127 | 574.20 |
| XXYLT1 | rs13079493 | chr3 | 195439305 | T | A | 0.16 | 0.01 | 0.12 | 35216 | 2.75E-39 | 171.95 |
| XXYLT1 | rs13084875 | chr3 | 195869765 | T | C | -0.06 | 0.01 | 0.26 | 35205 | 4.23E-09 | 34.51 |
| XXYLT1 | rs141363947 | chr3 | 195580642 | A | G | -0.14 | 0.02 | 0.04 | 35211 | 6.87E-11 | 42.55 |
| XXYLT1 | rs142467716 | chr3 | 195048234 | T | C | 0.22 | 0.03 | 0.01 | 35229 | 2.58E-11 | 44.47 |
| XXYLT1 | rs147857739 | chr3 | 195534195 | T | C | -0.12 | 0.02 | 0.04 | 35209 | 8.17E-09 | 33.23 |
| XXYLT1 | rs201377713 | chr3 | 195499459 | G | A | 0.20 | 0.02 | 0.04 | 35209 | 2.92E-21 | 89.60 |
| XXYLT1 | rs2410845 | chr3 | 195133293 | C | A | 0.13 | 0.01 | 0.13 | 35228 | 6.36E-27 | 115.41 |
| XXYLT1 | rs2720959 | chr3 | 195049339 | G | A | -0.05 | 0.01 | 0.45 | 35225 | 3.82E-11 | 43.71 |
| XXYLT1 | rs34182638 | chr3 | 195476405 | G | A | 0.10 | 0.02 | 0.08 | 35209 | 1.35E-10 | 41.24 |
| XXYLT1 | rs534994266 | chr3 | 195619664 | A | C | 0.13 | 0.01 | 0.09 | 35211 | 3.05E-19 | 80.40 |
| XXYLT1 | rs55947051 | chr3 | 195062304 | C | T | 0.16 | 0.01 | 0.19 | 35227 | 8.21E-55 | 243.10 |
| XXYLT1 | rs56170710 | chr3 | 195178331 | A | C | 0.15 | 0.02 | 0.03 | 35228 | 2.14E-10 | 40.33 |
| XXYLT1 | rs62285633 | chr3 | 195473228 | G | A | -0.09 | 0.01 | 0.21 | 35212 | 1.57E-17 | 72.62 |
| XXYLT1 | rs62290320 | chr3 | 195109253 | T | C | -0.07 | 0.01 | 0.18 | 35227 | 2.53E-10 | 40.01 |
| XXYLT1 | rs6437454 | chr3 | 195071402 | G | T | 0.14 | 0.01 | 0.19 | 35224 | 7.31E-42 | 183.76 |
| XXYLT1 | rs72611105 | chr3 | 195288902 | C | T | -0.15 | 0.02 | 0.05 | 35227 | 4.54E-16 | 65.98 |
| XXYLT1 | rs73085388 | chr3 | 195827843 | T | G | 0.10 | 0.02 | 0.06 | 35205 | 1.90E-09 | 36.08 |
| XXYLT1 | rs73206696 | chr3 | 195345249 | G | A | -0.14 | 0.02 | 0.05 | 35227 | 2.81E-13 | 53.34 |
| XXYLT1 | rs75802045 | chr3 | 195276586 | A | C | -0.18 | 0.02 | 0.03 | 35228 | 1.77E-15 | 63.31 |
| XXYLT1 | rs7613798 | chr3 | 195057556 | G | C | 0.06 | 0.01 | 0.40 | 35226 | 6.17E-12 | 47.27 |
| XXYLT1 | rs7631192 | chr3 | 195098291 | T | C | 0.08 | 0.01 | 0.21 | 35227 | 2.86E-15 | 62.36 |
| XXYLT1 | rs76326750 | chr3 | 195136249 | T | C | -0.19 | 0.03 | 0.03 | 35228 | 6.69E-14 | 56.15 |
| XXYLT1 | rs76870863 | chr3 | 195258327 | A | G | -0.15 | 0.02 | 0.08 | 35227 | 3.99E-23 | 98.09 |
| XXYLT1 | rs78532171 | chr3 | 195474497 | T | C | 0.13 | 0.01 | 0.19 | 35212 | 9.86E-35 | 151.11 |
| XXYLT1 | rs9817363 | chr3 | 195054861 | T | G | -0.07 | 0.01 | 0.30 | 35225 | 1.27E-16 | 68.49 |
| XXYLT1 | rs9870813 | chr3 | 195203401 | T | C | -0.07 | 0.01 | 0.42 | 35227 | 1.19E-18 | 77.71 |
| XXYLT1 | rs9886989 | chr3 | 195567882 | T | G | -0.07 | 0.01 | 0.49 | 35209 | 3.89E-17 | 70.83 |
| HSD17B14 | rs147540796 | chr19 | 48794238 | T | C | 0.13 | 0.02 | 0.06 | 35375 | 2.08E-14 | 58.45 |
| HSD17B14 | rs35299026 | chr19 | 48815123 | A | G | -0.16 | 0.02 | 0.05 | 35377 | 3.01E-19 | 80.42 |
| HSD17B14 | rs3760787 | chr19 | 48832898 | T | C | -0.05 | 0.01 | 0.25 | 35377 | 3.28E-09 | 35.01 |
| HSD17B14 | rs524 | chr19 | 48875910 | T | C | -0.07 | 0.01 | 0.28 | 35379 | 1.67E-14 | 58.88 |
| PLAU | rs112950248 | chr10 | 73765649 | G | A | -0.37 | 0.02 | 0.06 | 35377 | 2.31E-99 | 447.65 |
| PLAU | rs113248511 | chr10 | 73972580 | G | T | 0.16 | 0.02 | 0.03 | 35367 | 6.67E-12 | 47.12 |
| PLAU | rs113667161 | chr10 | 73950267 | C | G | -0.36 | 0.02 | 0.04 | 35366 | 6.43E-65 | 289.46 |
| PLAU | rs11598687 | chr10 | 74849813 | C | G | -0.21 | 0.04 | 0.01 | 35363 | 4.48E-09 | 34.40 |
| PLAU | rs116843906 | chr10 | 74010760 | C | T | -0.12 | 0.02 | 0.04 | 35370 | 3.22E-08 | 30.57 |
| PLAU | rs117866313 | chr10 | 73028922 | T | G | 0.12 | 0.02 | 0.06 | 35371 | 4.91E-11 | 43.21 |
| PLAU | rs11817132 | chr10 | 74151197 | C | G | 0.11 | 0.01 | 0.16 | 35369 | 3.92E-23 | 98.12 |
| PLAU | rs146287686 | chr10 | 73937934 | A | G | -0.30 | 0.03 | 0.02 | 35366 | 2.44E-21 | 89.95 |
| PLAU | rs150014740 | chr10 | 74022784 | A | G | -0.08 | 0.01 | 0.11 | 35370 | 9.67E-09 | 32.91 |
| PLAU | rs150026080 | chr10 | 73596323 | T | C | -0.43 | 0.02 | 0.04 | 35370 | 6.85E-96 | 431.68 |
| PLAU | rs2140409 | chr10 | 72973554 | A | T | 0.08 | 0.01 | 0.34 | 35371 | 1.91E-21 | 90.44 |
| PLAU | rs2227581 | chr10 | 73912117 | A | G | -0.11 | 0.01 | 0.10 | 35371 | 1.68E-14 | 58.87 |
| PLAU | rs2633298 | chr10 | 73906581 | G | C | -0.28 | 0.01 | 0.28 | 35372 | 1.00E-200 | 950.29 |
| PLAU | rs2664283 | chr10 | 73866615 | C | T | -0.19 | 0.02 | 0.08 | 35376 | 1.28E-33 | 146.02 |
| PLAU | rs41280378 | chr10 | 72932888 | G | T | 0.16 | 0.02 | 0.05 | 35372 | 1.69E-15 | 63.39 |
| PLAU | rs4745718 | chr10 | 73643030 | C | T | 0.09 | 0.01 | 0.10 | 35370 | 4.54E-11 | 43.36 |
| PLAU | rs4746137 | chr10 | 73606810 | C | T | 0.07 | 0.01 | 0.25 | 35369 | 3.14E-13 | 53.12 |
| PLAU | rs4746210 | chr10 | 74634215 | ATTTTTTTTG | TTTTTTTTTG | -0.14 | 0.02 | 0.05 | 35368 | 1.14E-12 | 50.59 |
| PLAU | rs4746215 | chr10 | 74657041 | TAG | TAA | 0.08 | 0.01 | 0.14 | 35368 | 3.80E-10 | 39.21 |
| PLAU | rs61864473 | chr10 | 73802214 | T | C | 0.31 | 0.03 | 0.02 | 35376 | 3.82E-22 | 93.62 |
| PLAU | rs61864538 | chr10 | 73942116 | G | T | 0.08 | 0.01 | 0.16 | 35366 | 1.96E-12 | 49.52 |
| PLAU | rs61865811 | chr10 | 73140523 | C | A | -0.17 | 0.01 | 0.17 | 35372 | 5.16E-55 | 244.05 |
| PLAU | rs7069013 | chr10 | 74061038 | G | T | -0.26 | 0.02 | 0.03 | 35369 | 6.35E-29 | 124.56 |
| PLAU | rs7094073 | chr10 | 73944743 | T | C | 0.13 | 0.01 | 0.19 | 35366 | 6.12E-33 | 142.92 |
| PLAU | rs71471657 | chr10 | 74019919 | CAGGCGTGGTGGCGGGCGC | CGGGCGTGGTGGCGGGCGC | 0.14 | 0.02 | 0.05 | 35370 | 7.20E-13 | 51.48 |
| PLAU | rs72812293 | chr10 | 73318921 | G | A | 0.13 | 0.01 | 0.12 | 35372 | 2.27E-22 | 94.64 |
| PLAU | rs77468362 | chr10 | 73851016 | T | C | 0.09 | 0.02 | 0.07 | 35376 | 3.11E-08 | 30.64 |
| PLAU | rs79445670 | chr10 | 73167333 | T | G | -0.26 | 0.04 | 0.01 | 35373 | 2.63E-13 | 53.46 |
| TREML2 | rs10485325 | chr6 | 41402809 | T | A | 0.11 | 0.01 | 0.26 | 35368 | 2.63E-30 | 130.88 |
| TREML2 | rs10947920 | chr6 | 40837968 | T | C | 0.08 | 0.01 | 0.21 | 35366 | 7.20E-13 | 51.49 |
| TREML2 | rs111583820 | chr6 | 42157486 | C | G | -0.12 | 0.02 | 0.05 | 35360 | 3.78E-09 | 34.73 |
| TREML2 | rs112392367 | chr6 | 41386120 | C | T | -0.17 | 0.03 | 0.03 | 35368 | 3.25E-11 | 44.02 |
| TREML2 | rs112449894 | chr6 | 41494763 | C | T | -0.09 | 0.02 | 0.07 | 35361 | 3.49E-08 | 30.41 |
| TREML2 | rs112814338 | chr6 | 41016334 | A | G | 0.22 | 0.03 | 0.02 | 35372 | 1.26E-11 | 45.87 |
| TREML2 | rs113009556 | chr6 | 41602725 | C | T | 0.12 | 0.02 | 0.04 | 35360 | 3.23E-08 | 30.56 |
| TREML2 | rs113384506 | chr6 | 41523833 | C | T | -0.14 | 0.02 | 0.04 | 35356 | 9.76E-10 | 37.37 |
| TREML2 | rs115550458 | chr6 | 40946743 | C | T | -0.16 | 0.03 | 0.03 | 35374 | 1.00E-09 | 37.32 |
| TREML2 | rs115782355 | chr6 | 41185506 | G | A | -0.31 | 0.04 | 0.01 | 35372 | 1.88E-17 | 72.26 |
| TREML2 | rs115960053 | chr6 | 40808300 | G | A | 0.40 | 0.03 | 0.02 | 35365 | 1.09E-43 | 192.12 |
| TREML2 | rs11753691 | chr6 | 40342263 | T | C | 0.07 | 0.01 | 0.41 | 35365 | 5.50E-17 | 70.15 |
| TREML2 | rs11961292 | chr6 | 40694447 | A | C | -0.09 | 0.01 | 0.19 | 35365 | 1.37E-17 | 72.88 |
| TREML2 | rs11963920 | chr6 | 41046183 | C | T | -0.26 | 0.02 | 0.04 | 35372 | 2.33E-28 | 121.98 |
| TREML2 | rs12197041 | chr6 | 41493891 | T | G | 0.07 | 0.01 | 0.17 | 35357 | 4.01E-10 | 39.11 |
| TREML2 | rs12200388 | chr6 | 42042682 | G | C | 0.07 | 0.01 | 0.17 | 35363 | 1.29E-09 | 36.83 |
| TREML2 | rs12209760 | chr6 | 40595181 | A | G | 0.13 | 0.02 | 0.05 | 35366 | 4.05E-10 | 39.09 |
| TREML2 | rs12214664 | chr6 | 40616131 | G | A | 0.20 | 0.02 | 0.08 | 35366 | 1.06E-37 | 164.71 |
| TREML2 | rs12528250 | chr6 | 40444210 | C | T | 0.14 | 0.02 | 0.05 | 35361 | 2.42E-13 | 53.62 |
| TREML2 | rs13196620 | chr6 | 40641068 | G | A | -0.07 | 0.01 | 0.33 | 35360 | 1.09E-13 | 55.20 |
| TREML2 | rs13197470 | chr6 | 41466834 | TTCTGGTACC | TTCCGGTACC | 0.11 | 0.02 | 0.06 | 35366 | 1.85E-08 | 31.64 |
| TREML2 | rs139456480 | chr6 | 40529205 | T | C | 0.44 | 0.03 | 0.02 | 35365 | 1.25E-40 | 178.11 |
| TREML2 | rs140136183 | chr6 | 41615734 | C | G | 0.12 | 0.02 | 0.04 | 35363 | 6.49E-09 | 33.68 |
| TREML2 | rs143522603 | chr6 | 40849512 | T | C | -0.18 | 0.03 | 0.03 | 35367 | 1.36E-11 | 45.72 |
| TREML2 | rs146298635 | chr6 | 41030585 | A | G | 0.38 | 0.04 | 0.01 | 35372 | 1.06E-21 | 91.59 |
| TREML2 | rs147373305 | chr6 | 40893392 | A | G | 0.36 | 0.02 | 0.03 | 35376 | 1.21E-48 | 214.84 |
| TREML2 | rs150504577 | chr6 | 41382614 | C | T | -0.19 | 0.02 | 0.09 | 35368 | 1.68E-35 | 154.63 |
| TREML2 | rs151307515 | chr6 | 40713913 | T | C | -0.26 | 0.03 | 0.03 | 35365 | 3.93E-22 | 93.56 |
| TREML2 | rs17701319 | chr6 | 41273665 | T | G | -0.27 | 0.02 | 0.05 | 35369 | 9.91E-41 | 178.57 |
| TREML2 | rs17704650 | chr6 | 41591791 | T | G | 0.16 | 0.03 | 0.03 | 35361 | 3.99E-09 | 34.63 |
| TREML2 | rs181021675 | chr6 | 41521371 | A | G | 0.24 | 0.04 | 0.01 | 35354 | 1.98E-09 | 35.99 |
| TREML2 | rs186497231 | chr6 | 41605405 | G | A | -0.19 | 0.03 | 0.03 | 35361 | 1.30E-11 | 45.82 |
| TREML2 | rs1968872 | chr6 | 41217159 | A | G | -0.11 | 0.02 | 0.09 | 35375 | 4.14E-13 | 52.57 |
| TREML2 | rs1983798 | chr6 | 41415193 | T | C | -0.07 | 0.01 | 0.34 | 35366 | 3.51E-14 | 57.43 |
| TREML2 | rs2006080 | chr6 | 41199030 | C | T | 0.17 | 0.01 | 0.21 | 35371 | 2.13E-56 | 250.38 |
| TREML2 | rs2038568 | chr6 | 41190394 | C | G | -0.15 | 0.02 | 0.04 | 35372 | 1.63E-12 | 49.88 |
| TREML2 | rs2268412 | chr6 | 41640131 | A | G | 0.07 | 0.01 | 0.22 | 35359 | 1.02E-10 | 41.78 |
| TREML2 | rs2473596 | chr6 | 40589376 | G | A | 0.06 | 0.01 | 0.47 | 35366 | 4.92E-12 | 47.72 |
| TREML2 | rs2477829 | chr6 | 41462812 | G | T | -0.06 | 0.01 | 0.48 | 35363 | 2.08E-10 | 40.39 |
| TREML2 | rs2477844 | chr6 | 41523487 | T | C | -0.05 | 0.01 | 0.33 | 35356 | 1.03E-08 | 32.79 |
| TREML2 | rs2496637 | chr6 | 41487318 | C | T | 0.06 | 0.01 | 0.44 | 35358 | 2.27E-12 | 49.23 |
| TREML2 | rs35248939 | chr6 | 40641654 | G | A | 0.06 | 0.01 | 0.44 | 35360 | 2.20E-13 | 53.81 |
| TREML2 | rs35528198 | chr6 | 42152600 | T | C | 0.09 | 0.01 | 0.11 | 35361 | 5.38E-12 | 47.54 |
| TREML2 | rs371514020 | chr6 | 41454224 | A | G | -0.13 | 0.02 | 0.04 | 35365 | 1.32E-10 | 41.27 |
| TREML2 | rs375681998 | chr6 | 41283717 | AAAAAACACACAC | AAAACACACACAC | -0.21 | 0.03 | 0.02 | 35371 | 1.04E-13 | 55.29 |
| TREML2 | rs3761781 | chr6 | 41777191 | CGTTAATTCAAGTTACAATA | CGTTAATTCAAGTTACAATG | -0.08 | 0.01 | 0.16 | 35368 | 1.24E-10 | 41.39 |
| TREML2 | rs3804276 | chr6 | 41277496 | T | C | -0.12 | 0.01 | 0.49 | 35372 | 2.23E-46 | 204.45 |
| TREML2 | rs3857580 | chr6 | 41248596 | G | A | -0.26 | 0.01 | 0.40 | 35373 | 4.65E-195 | 887.72 |
| TREML2 | rs41273764 | chr6 | 41163317 | G | T | -0.21 | 0.02 | 0.04 | 35372 | 1.34E-19 | 82.02 |
| TREML2 | rs4365924 | chr6 | 42027623 | C | T | 0.06 | 0.01 | 0.25 | 35363 | 2.40E-10 | 40.11 |
| TREML2 | rs4711637 | chr6 | 40484315 | A | G | 0.13 | 0.01 | 0.10 | 35360 | 9.20E-20 | 82.76 |
| TREML2 | rs4711652 | chr6 | 40885000 | A | G | 0.12 | 0.01 | 0.44 | 35372 | 1.08E-42 | 187.53 |
| TREML2 | rs4711674 | chr6 | 41395028 | G | A | -0.08 | 0.01 | 0.18 | 35368 | 3.92E-12 | 48.16 |
| TREML2 | rs55816716 | chr6 | 40704863 | G | C | 0.15 | 0.01 | 0.20 | 35365 | 1.38E-42 | 187.06 |
| TREML2 | rs56104198 | chr6 | 41216488 | T | C | 0.66 | 0.02 | 0.06 | 35375 | 1.00E-200 | 1317.80 |
| TREML2 | rs56180584 | chr6 | 42094809 | C | G | 0.10 | 0.01 | 0.10 | 35363 | 3.82E-13 | 52.73 |
| TREML2 | rs56272207 | chr6 | 41413362 | G | C | -0.16 | 0.02 | 0.05 | 35368 | 2.84E-15 | 62.37 |
| TREML2 | rs56332525 | chr6 | 41491595 | C | A | 0.07 | 0.01 | 0.16 | 35360 | 7.20E-09 | 33.48 |
| TREML2 | rs605991 | chr6 | 41394882 | TG | TA | -0.12 | 0.01 | 0.20 | 35369 | 5.58E-27 | 115.67 |
| TREML2 | rs61738668 | chr6 | 41224163 | A | G | -0.24 | 0.02 | 0.04 | 35375 | 1.12E-27 | 118.86 |
| TREML2 | rs62392778 | chr6 | 40323242 | T | C | -0.09 | 0.02 | 0.09 | 35365 | 1.52E-08 | 32.02 |
| TREML2 | rs62397195 | chr6 | 40978993 | A | G | -0.17 | 0.02 | 0.05 | 35372 | 1.20E-16 | 68.61 |
| TREML2 | rs6458198 | chr6 | 41193736 | C | A | -0.29 | 0.01 | 0.34 | 35372 | 1.00E-200 | 1000.19 |
| TREML2 | rs6918969 | chr6 | 41082156 | C | T | -0.20 | 0.01 | 0.25 | 35372 | 1.22E-94 | 425.95 |
| TREML2 | rs6923862 | chr6 | 40743634 | C | G | -0.12 | 0.01 | 0.28 | 35366 | 2.70E-33 | 144.52 |
| TREML2 | rs6929418 | chr6 | 41339856 | G | A | 0.16 | 0.01 | 0.24 | 35369 | 1.39E-58 | 260.39 |
| TREML2 | rs71573307 | chr6 | 40443686 | T | G | 0.06 | 0.01 | 0.25 | 35362 | 5.20E-09 | 34.11 |
| TREML2 | rs71573331 | chr6 | 40973880 | A | C | -0.19 | 0.02 | 0.07 | 35371 | 1.57E-29 | 127.33 |
| TREML2 | rs72851967 | chr6 | 40957516 | A | G | -0.26 | 0.03 | 0.02 | 35371 | 2.11E-19 | 81.13 |
| TREML2 | rs72851989 | chr6 | 40979606 | T | C | -0.14 | 0.01 | 0.46 | 35372 | 2.29E-58 | 259.44 |
| TREML2 | rs75469439 | chr6 | 40475770 | G | A | -0.13 | 0.02 | 0.05 | 35362 | 6.02E-10 | 38.31 |
| TREML2 | rs75569095 | chr6 | 41583193 | A | G | 0.08 | 0.01 | 0.15 | 35359 | 1.02E-11 | 46.29 |
| TREML2 | rs75839893 | chr6 | 40641884 | C | T | 0.16 | 0.03 | 0.03 | 35360 | 4.30E-10 | 38.97 |
| TREML2 | rs76292476 | chr6 | 41008808 | A | G | 0.47 | 0.02 | 0.04 | 35372 | 5.42E-92 | 413.81 |
| TREML2 | rs76598786 | chr6 | 41115335 | T | G | 0.65 | 0.02 | 0.04 | 35372 | 7.35E-186 | 845.36 |
| TREML2 | rs77279417 | chr6 | 40936828 | A | T | 0.26 | 0.02 | 0.04 | 35375 | 9.40E-30 | 128.35 |
| TREML2 | rs77303168 | chr6 | 41394702 | T | C | 0.19 | 0.02 | 0.03 | 35369 | 1.16E-14 | 59.61 |
| TREML2 | rs7760176 | chr6 | 40709088 | G | A | 0.17 | 0.01 | 0.23 | 35365 | 3.76E-64 | 285.96 |
| TREML2 | rs7761534 | chr6 | 41377837 | C | T | 0.31 | 0.02 | 0.06 | 35368 | 3.90E-62 | 276.71 |
| TREML2 | rs7762551 | chr6 | 41469521 | T | C | -0.06 | 0.01 | 0.42 | 35364 | 2.84E-12 | 48.80 |
| TREML2 | rs77645786 | chr6 | 40496755 | T | C | 0.23 | 0.03 | 0.02 | 35363 | 7.21E-13 | 51.48 |
| TREML2 | rs77787390 | chr6 | 40649100 | T | C | -0.12 | 0.02 | 0.05 | 35360 | 3.46E-09 | 34.90 |
| TREML2 | rs77889896 | chr6 | 41474873 | G | T | 0.10 | 0.01 | 0.15 | 35364 | 2.40E-17 | 71.78 |
| TREML2 | rs78055825 | chr6 | 41251036 | T | C | -0.25 | 0.03 | 0.02 | 35373 | 5.10E-16 | 65.75 |
| TREML2 | rs78462966 | chr6 | 41458692 | T | C | 0.24 | 0.02 | 0.08 | 35365 | 9.91E-54 | 238.15 |
| TREML2 | rs79137408 | chr6 | 40478076 | A | G | -0.17 | 0.03 | 0.02 | 35361 | 5.79E-09 | 33.90 |
| TREML2 | rs79627312 | chr6 | 40563387 | C | T | 0.38 | 0.03 | 0.02 | 35366 | 1.37E-38 | 168.77 |
| TREML2 | rs814837 | chr6 | 41421773 | T | C | -0.09 | 0.01 | 0.36 | 35363 | 3.70E-23 | 98.25 |
| TREML2 | rs912880 | chr6 | 41502046 | G | A | 0.08 | 0.01 | 0.30 | 35360 | 2.00E-16 | 67.59 |
| TREML2 | rs914508 | chr6 | 40889507 | A | G | -0.14 | 0.02 | 0.05 | 35374 | 1.96E-12 | 49.52 |
| TREML2 | rs9367080 | chr6 | 40809857 | T | A | -0.05 | 0.01 | 0.36 | 35365 | 2.91E-09 | 35.24 |
| TREML2 | rs9369259 | chr6 | 41042537 | T | A | 0.17 | 0.02 | 0.05 | 35372 | 3.85E-17 | 70.85 |
| TREML2 | rs9369272 | chr6 | 41345971 | G | A | 0.10 | 0.01 | 0.33 | 35369 | 3.17E-26 | 112.24 |
| TREML2 | rs9369287 | chr6 | 41535740 | T | C | 0.15 | 0.02 | 0.03 | 35356 | 2.97E-09 | 35.20 |
| TREML2 | rs9394694 | chr6 | 40438620 | C | T | -0.12 | 0.01 | 0.10 | 35363 | 2.06E-15 | 63.01 |
| TREML2 | rs9471531 | chr6 | 41274462 | G | A | 0.14 | 0.01 | 0.35 | 35372 | 1.12E-51 | 228.73 |
| TREML2 | rs9471574 | chr6 | 41332161 | A | G | 0.06 | 0.01 | 0.45 | 35370 | 4.15E-13 | 52.57 |
| TREML2 | rs991762 | chr6 | 40955671 | T | C | 0.33 | 0.01 | 0.21 | 35371 | 1.00E-200 | 1004.45 |
| MST1 | rs10446468 | chr3 | 49399591 | TT | TC | 0.64 | 0.00 | 0.43 | 35344 | 1.00E-200 | 24140.24 |
| MST1 | rs1060970 | chr3 | 49671336 | C | T | 0.58 | 0.01 | 0.19 | 35344 | 1.00E-200 | 11899.40 |
| MST1 | rs113014003 | chr3 | 50562080 | G | A | -0.36 | 0.01 | 0.14 | 35343 | 1.00E-200 | 967.99 |
| MST1 | rs114397936 | chr3 | 50429048 | G | A | 0.28 | 0.03 | 0.02 | 35341 | 7.00E-24 | 101.53 |
| MST1 | rs114508808 | chr3 | 49314794 | A | G | 0.41 | 0.03 | 0.02 | 35344 | 2.75E-35 | 153.65 |
| MST1 | rs114918429 | chr3 | 49349106 | A | T | -1.17 | 0.04 | 0.01 | 35344 | 1.00E-200 | 1034.61 |
| MST1 | rs115258642 | chr3 | 50221805 | T | C | -0.58 | 0.02 | 0.06 | 35343 | 1.00E-200 | 1105.47 |
| MST1 | rs115548824 | chr3 | 49658718 | G | C | 0.52 | 0.03 | 0.02 | 35344 | 1.73E-54 | 241.62 |
| MST1 | rs115552063 | chr3 | 49642393 | A | G | 0.36 | 0.04 | 0.01 | 35344 | 5.98E-22 | 92.73 |
| MST1 | rs116113836 | chr3 | 48775161 | A | G | 0.27 | 0.02 | 0.05 | 35344 | 1.57E-45 | 200.55 |
| MST1 | rs116285936 | chr3 | 49158519 | T | C | -1.21 | 0.03 | 0.02 | 35344 | 1.00E-200 | 1318.57 |
| MST1 | rs138838615 | chr3 | 48729234 | A | G | -0.95 | 0.01 | 0.06 | 35343 | 1.00E-200 | 11245.34 |
| MST1 | rs142412647 | chr3 | 49073834 | G | A | 0.28 | 0.02 | 0.03 | 35344 | 1.03E-35 | 155.61 |
| MST1 | rs143026203 | chr3 | 50418792 | A | G | 0.44 | 0.02 | 0.04 | 35341 | 1.81E-92 | 415.98 |
| MST1 | rs143786338 | chr3 | 50608258 | G | C | 0.46 | 0.03 | 0.03 | 35343 | 1.25E-67 | 301.91 |
| MST1 | rs144519232 | chr3 | 48828880 | G | C | 0.52 | 0.01 | 0.10 | 35344 | 1.00E-200 | 5143.06 |
| MST1 | rs145852774 | chr3 | 50568191 | A | C | 0.50 | 0.02 | 0.04 | 35343 | 1.56E-122 | 554.14 |
| MST1 | rs146714917 | chr3 | 50541148 | C | T | 0.44 | 0.03 | 0.02 | 35343 | 9.68E-40 | 174.04 |
| MST1 | rs147919666 | chr3 | 50169988 | C | A | 0.32 | 0.03 | 0.02 | 35343 | 4.60E-27 | 116.06 |
| MST1 | rs148834251 | chr3 | 49523775 | C | T | -1.14 | 0.01 | 0.04 | 35344 | 1.00E-200 | 10098.70 |
| MST1 | rs149044789 | chr3 | 48768352 | CAGACAGACAGACAGACAG | CAGAGAGACAGACAGACAG | 0.40 | 0.04 | 0.01 | 35342 | 8.35E-25 | 105.75 |
| MST1 | rs150412151 | chr3 | 49292098 | T | C | 0.51 | 0.03 | 0.02 | 35344 | 9.43E-61 | 270.35 |
| MST1 | rs150426469 | chr3 | 49042062 | T | C | -0.83 | 0.02 | 0.03 | 35344 | 1.00E-200 | 1310.81 |
| MST1 | rs150764102 | chr3 | 50562957 | T | C | -0.19 | 0.03 | 0.03 | 35343 | 8.46E-13 | 51.17 |
| MST1 | rs182210511 | chr3 | 48925196 | T | C | 0.52 | 0.03 | 0.02 | 35344 | 8.64E-69 | 307.24 |
| MST1 | rs188551769 | chr3 | 48686026 | T | G | -0.61 | 0.01 | 0.07 | 35342 | 1.00E-200 | 5204.10 |
| MST1 | rs188683902 | chr3 | 48716996 | C | T | -1.00 | 0.01 | 0.03 | 35342 | 1.00E-200 | 6932.51 |
| MST1 | rs191413135 | chr3 | 49708248 | A | G | -0.73 | 0.04 | 0.01 | 35344 | 1.40E-78 | 352.20 |
| MST1 | rs1961527 | chr3 | 48727521 | G | A | 0.45 | 0.01 | 0.16 | 35343 | 1.00E-200 | 5968.19 |
| MST1 | rs2003485 | chr3 | 50139156 | T | C | 0.36 | 0.00 | 0.30 | 35344 | 1.00E-200 | 6088.08 |
| MST1 | rs2526388 | chr3 | 50137453 | C | T | -0.29 | 0.01 | 0.26 | 35344 | 1.00E-200 | 957.36 |
| MST1 | rs35915701 | chr3 | 50264001 | G | C | 0.37 | 0.02 | 0.04 | 35343 | 3.87E-73 | 327.19 |
| MST1 | rs4688718 | chr3 | 50484632 | A | G | -0.10 | 0.01 | 0.36 | 35341 | 1.45E-33 | 145.79 |
| MST1 | rs62262721 | chr3 | 49731494 | C | T | 0.44 | 0.02 | 0.04 | 35344 | 3.90E-100 | 451.18 |
| MST1 | rs6806356 | chr3 | 48690203 | A | G | -0.47 | 0.01 | 0.21 | 35341 | 1.00E-200 | 8083.70 |
| MST1 | rs73086963 | chr3 | 50598159 | T | C | -0.55 | 0.02 | 0.03 | 35342 | 3.68E-112 | 506.47 |
| MST1 | rs7611164 | chr3 | 49471395 | G | T | 0.40 | 0.02 | 0.08 | 35344 | 4.13E-155 | 703.87 |
| MST1 | rs7613681 | chr3 | 50401362 | C | T | 0.42 | 0.00 | 0.49 | 35341 | 1.00E-200 | 9758.46 |
| MST1 | rs76644847 | chr3 | 50215214 | T | G | 0.47 | 0.04 | 0.01 | 35343 | 9.94E-38 | 164.82 |
| MST1 | rs77068319 | chr3 | 49761970 | C | G | 0.51 | 0.02 | 0.05 | 35344 | 4.85E-152 | 689.78 |
| MST1 | rs78104031 | chr3 | 50660110 | G | A | -0.36 | 0.02 | 0.04 | 35342 | 9.59E-69 | 307.02 |
| MST1 | rs78501564 | chr3 | 50030494 | T | C | 0.45 | 0.02 | 0.04 | 35344 | 3.20E-116 | 525.14 |
| MST1 | rs79402695 | chr3 | 50520942 | A | G | -0.38 | 0.03 | 0.02 | 35343 | 2.40E-47 | 208.87 |
| MST1 | rs79685183 | chr3 | 50400642 | T | C | -0.60 | 0.03 | 0.02 | 35341 | 3.05E-88 | 396.56 |
| MST1 | rs9811027 | chr3 | 48687581 | C | G | -0.77 | 0.01 | 0.11 | 35340 | 1.00E-200 | 13999.09 |
| MST1 | rs9852677 | chr3 | 50254185 | A | G | -0.64 | 0.02 | 0.03 | 35343 | 4.12E-160 | 726.91 |
| NID2 | rs10146989 | chr14 | 52011149 | A | G | -0.32 | 0.01 | 0.29 | 35369 | 1.00E-200 | 1309.72 |
| NID2 | rs10148939 | chr14 | 51986941 | C | A | -0.42 | 0.01 | 0.11 | 35369 | 1.00E-200 | 1114.38 |
| NID2 | rs10484085 | chr14 | 51614286 | A | G | 0.08 | 0.01 | 0.18 | 35368 | 4.45E-14 | 56.96 |
| NID2 | rs111304192 | chr14 | 51973153 | G | A | -0.14 | 0.02 | 0.04 | 35368 | 5.82E-11 | 42.88 |
| NID2 | rs11157865 | chr14 | 51900357 | T | C | 0.10 | 0.01 | 0.43 | 35369 | 4.51E-32 | 138.93 |
| NID2 | rs111683636 | chr14 | 51719554 | G | A | -0.17 | 0.03 | 0.02 | 35370 | 1.39E-09 | 36.69 |
| NID2 | rs111719845 | chr14 | 52089369 | G | A | 0.30 | 0.02 | 0.06 | 35367 | 3.96E-64 | 285.84 |
| NID2 | rs111849006 | chr14 | 52115175 | A | G | 0.22 | 0.01 | 0.19 | 35367 | 2.28E-98 | 443.06 |
| NID2 | rs111863224 | chr14 | 52074165 | A | T | -0.39 | 0.03 | 0.02 | 35366 | 4.36E-41 | 180.20 |
| NID2 | rs112760697 | chr14 | 51771757 | T | C | 0.18 | 0.02 | 0.06 | 35367 | 1.35E-26 | 113.92 |
| NID2 | rs112834253 | chr14 | 52288382 | T | C | 0.23 | 0.03 | 0.03 | 35367 | 5.75E-19 | 79.15 |
| NID2 | rs113387081 | chr14 | 51264289 | T | C | -0.31 | 0.04 | 0.01 | 35372 | 1.11E-14 | 59.69 |
| NID2 | rs1151579 | chr14 | 52011117 | A | G | 0.05 | 0.01 | 0.26 | 35369 | 1.86E-08 | 31.63 |
| NID2 | rs11620718 | chr14 | 51839980 | G | C | 0.15 | 0.01 | 0.15 | 35370 | 4.42E-39 | 171.03 |
| NID2 | rs11621171 | chr14 | 52307102 | C | A | -0.32 | 0.02 | 0.04 | 35363 | 4.78E-51 | 225.83 |
| NID2 | rs116877899 | chr14 | 52336534 | C | A | 0.20 | 0.02 | 0.03 | 35365 | 2.99E-17 | 71.34 |
| NID2 | rs117114022 | chr14 | 52019492 | G | C | -0.15 | 0.03 | 0.02 | 35369 | 2.88E-08 | 30.78 |
| NID2 | rs117663230 | chr14 | 52019582 | A | G | -0.31 | 0.03 | 0.02 | 35369 | 6.30E-24 | 101.74 |
| NID2 | rs117845304 | chr14 | 52143192 | T | C | 0.31 | 0.02 | 0.04 | 35366 | 3.29E-44 | 194.50 |
| NID2 | rs118035768 | chr14 | 52059726 | G | T | 0.19 | 0.03 | 0.02 | 35367 | 8.26E-11 | 42.19 |
| NID2 | rs118075982 | chr14 | 52056274 | G | A | 0.20 | 0.03 | 0.02 | 35367 | 7.72E-12 | 46.83 |
| NID2 | rs118089510 | chr14 | 51920732 | G | A | 0.12 | 0.02 | 0.04 | 35369 | 2.91E-08 | 30.76 |
| NID2 | rs118143454 | chr14 | 51682925 | AACAACACACT | GACAACACACT | 0.14 | 0.02 | 0.05 | 35370 | 2.28E-12 | 49.23 |
| NID2 | rs12432630 | chr14 | 52007106 | T | C | 0.27 | 0.02 | 0.04 | 35369 | 7.17E-32 | 138.02 |
| NID2 | rs12588519 | chr14 | 51904993 | C | T | -0.14 | 0.02 | 0.04 | 35369 | 1.14E-11 | 46.07 |
| NID2 | rs12885443 | chr14 | 51608935 | C | A | 0.07 | 0.01 | 0.22 | 35368 | 1.88E-12 | 49.61 |
| NID2 | rs139394488 | chr14 | 52194093 | G | A | -0.47 | 0.04 | 0.01 | 35366 | 1.30E-37 | 164.28 |
| NID2 | rs141578540 | chr14 | 51616256 | C | G | -0.21 | 0.04 | 0.01 | 35368 | 1.68E-09 | 36.31 |
| NID2 | rs141702292 | chr14 | 52198743 | G | A | -0.13 | 0.02 | 0.03 | 35366 | 3.33E-09 | 34.98 |
| NID2 | rs143229070 | chr14 | 51993356 | C | T | -0.30 | 0.04 | 0.01 | 35369 | 1.92E-15 | 63.14 |
| NID2 | rs144996426 | chr14 | 52992084 | T | G | -0.15 | 0.02 | 0.03 | 35366 | 3.32E-11 | 43.98 |
| NID2 | rs145454388 | chr14 | 52170332 | T | C | -0.25 | 0.02 | 0.03 | 35365 | 2.88E-26 | 112.42 |
| NID2 | rs145501594 | chr14 | 51773037 | T | C | -0.19 | 0.02 | 0.03 | 35367 | 1.43E-15 | 63.72 |
| NID2 | rs146735655 | chr14 | 51285980 | C | T | 0.13 | 0.02 | 0.03 | 35372 | 7.13E-09 | 33.50 |
| NID2 | rs149731447 | chr14 | 52070230 | T | C | -0.25 | 0.02 | 0.03 | 35366 | 1.14E-23 | 100.56 |
| NID2 | rs150110572 | chr14 | 51759483 | T | G | -0.08 | 0.01 | 0.10 | 35368 | 8.67E-09 | 33.11 |
| NID2 | rs150930175 | chr14 | 51990397 | T | C | 0.18 | 0.02 | 0.04 | 35369 | 3.46E-16 | 66.52 |
| NID2 | rs17252984 | chr14 | 52298272 | A | G | 0.09 | 0.01 | 0.08 | 35364 | 7.33E-09 | 33.44 |
| NID2 | rs17663972 | chr14 | 51580944 | G | T | -0.16 | 0.02 | 0.04 | 35368 | 5.63E-13 | 51.97 |
| NID2 | rs17831313 | chr14 | 51810244 | G | C | 0.07 | 0.01 | 0.18 | 35369 | 2.93E-10 | 39.72 |
| NID2 | rs181469889 | chr14 | 51751757 | A | G | -0.30 | 0.03 | 0.02 | 35367 | 1.66E-20 | 86.15 |
| NID2 | rs1956578 | chr14 | 51611247 | A | G | 0.10 | 0.02 | 0.06 | 35369 | 8.55E-10 | 37.63 |
| NID2 | rs2357443 | chr14 | 52152519 | C | T | 0.19 | 0.01 | 0.16 | 35365 | 1.35E-62 | 278.80 |
| NID2 | rs2357456 | chr14 | 52186532 | G | A | -0.17 | 0.02 | 0.06 | 35366 | 3.45E-23 | 98.37 |
| NID2 | rs28409499 | chr14 | 51059856 | G | C | -0.12 | 0.02 | 0.05 | 35373 | 2.74E-10 | 39.85 |
| NID2 | rs2884136 | chr14 | 51855998 | C | T | 0.17 | 0.01 | 0.28 | 35370 | 5.84E-77 | 344.76 |
| NID2 | rs34453557 | chr14 | 51383606 | T | C | -0.08 | 0.01 | 0.11 | 35373 | 2.94E-09 | 35.22 |
| NID2 | rs35170217 | chr14 | 52238799 | T | C | -0.18 | 0.02 | 0.04 | 35367 | 8.51E-19 | 78.38 |
| NID2 | rs35458896 | chr14 | 51973243 | G | C | 0.09 | 0.01 | 0.35 | 35368 | 1.15E-24 | 105.12 |
| NID2 | rs36016197 | chr14 | 51987660 | C | A | -0.32 | 0.01 | 0.22 | 35369 | 1.00E-200 | 1078.16 |
| NID2 | rs3818186 | chr14 | 52042783 | C | T | -0.35 | 0.01 | 0.20 | 35367 | 1.00E-200 | 1183.73 |
| NID2 | rs4901109 | chr14 | 51339831 | A | T | 0.17 | 0.03 | 0.02 | 35373 | 1.98E-09 | 35.99 |
| NID2 | rs4901151 | chr14 | 51756540 | C | T | 0.08 | 0.01 | 0.42 | 35367 | 5.98E-22 | 92.73 |
| NID2 | rs4901317 | chr14 | 52923587 | T | C | 0.06 | 0.01 | 0.34 | 35368 | 3.79E-11 | 43.72 |
| NID2 | rs539158291 | chr14 | 52639056 | C | G | 0.10 | 0.02 | 0.07 | 35367 | 5.55E-10 | 38.47 |
| NID2 | rs553939316 | chr14 | 52130137 | G | A | -0.23 | 0.02 | 0.03 | 35367 | 1.92E-21 | 90.41 |
| NID2 | rs56179523 | chr14 | 52223673 | T | C | 0.09 | 0.01 | 0.29 | 35367 | 2.87E-23 | 98.75 |
| NID2 | rs565351603 | chr14 | 52853812 | C | T | 0.24 | 0.04 | 0.01 | 35367 | 1.50E-10 | 41.02 |
| NID2 | rs56799730 | chr14 | 51687859 | C | A | -0.14 | 0.02 | 0.07 | 35369 | 1.71E-18 | 76.99 |
| NID2 | rs58168773 | chr14 | 51399503 | T | C | 0.06 | 0.01 | 0.31 | 35373 | 1.50E-11 | 45.53 |
| NID2 | rs61971478 | chr14 | 51922585 | G | C | 0.15 | 0.02 | 0.04 | 35369 | 6.72E-14 | 56.15 |
| NID2 | rs61971509 | chr14 | 52001614 | G | A | 0.25 | 0.01 | 0.16 | 35369 | 2.32E-114 | 516.61 |
| NID2 | rs62003545 | chr14 | 52944663 | G | A | -0.20 | 0.03 | 0.02 | 35368 | 1.36E-11 | 45.73 |
| NID2 | rs62003676 | chr14 | 52337138 | C | T | -0.11 | 0.02 | 0.05 | 35365 | 3.63E-10 | 39.30 |
| NID2 | rs6572804 | chr14 | 51942297 | A | T | -0.30 | 0.03 | 0.01 | 35369 | 5.32E-18 | 74.75 |
| NID2 | rs67565529 | chr14 | 52447597 | G | A | 0.11 | 0.01 | 0.24 | 35366 | 8.23E-30 | 128.62 |
| NID2 | rs708539 | chr14 | 52413986 | G | A | -0.16 | 0.03 | 0.02 | 35365 | 2.07E-09 | 35.91 |
| NID2 | rs71422024 | chr14 | 51511855 | T | C | 0.10 | 0.02 | 0.07 | 35369 | 4.39E-11 | 43.43 |
| NID2 | rs71422076 | chr14 | 52297887 | G | C | -0.18 | 0.01 | 0.13 | 35364 | 1.60E-50 | 223.43 |
| NID2 | rs7145865 | chr14 | 51775448 | A | G | -0.13 | 0.01 | 0.30 | 35367 | 2.19E-46 | 204.48 |
| NID2 | rs7147946 | chr14 | 51026243 | T | C | -0.06 | 0.01 | 0.22 | 35373 | 4.83E-09 | 34.26 |
| NID2 | rs7153059 | chr14 | 52151494 | T | C | -0.24 | 0.01 | 0.18 | 35367 | 8.96E-117 | 527.66 |
| NID2 | rs7160397 | chr14 | 51603619 | G | A | -0.10 | 0.02 | 0.06 | 35367 | 1.04E-08 | 32.77 |
| NID2 | rs72678129 | chr14 | 51869114 | C | T | 0.19 | 0.01 | 0.09 | 35369 | 4.20E-41 | 180.29 |
| NID2 | rs72682499 | chr14 | 52364392 | A | G | 0.22 | 0.01 | 0.09 | 35364 | 7.44E-56 | 247.89 |
| NID2 | rs72687121 | chr14 | 51011012 | A | C | -0.14 | 0.03 | 0.03 | 35373 | 1.46E-08 | 32.11 |
| NID2 | rs74566463 | chr14 | 51624660 | A | C | -0.13 | 0.01 | 0.09 | 35368 | 3.40E-19 | 80.19 |
| NID2 | rs75065699 | chr14 | 51931613 | A | G | 0.13 | 0.02 | 0.03 | 35368 | 1.60E-08 | 31.92 |
| NID2 | rs75073548 | chr14 | 51473150 | A | G | -0.13 | 0.02 | 0.06 | 35370 | 5.02E-15 | 61.25 |
| NID2 | rs75293060 | chr14 | 52364226 | G | C | -0.24 | 0.02 | 0.07 | 35365 | 3.73E-49 | 217.15 |
| NID2 | rs75294755 | chr14 | 52003775 | T | A | -0.40 | 0.03 | 0.02 | 35369 | 5.58E-32 | 138.52 |
| NID2 | rs75478143 | chr14 | 51889803 | C | T | -0.44 | 0.03 | 0.02 | 35368 | 1.20E-44 | 196.51 |
| NID2 | rs76200085 | chr14 | 51999784 | A | G | -0.21 | 0.02 | 0.03 | 35369 | 6.76E-20 | 83.38 |
| NID2 | rs76569378 | chr14 | 51920229 | TATT | TATC | -0.23 | 0.03 | 0.02 | 35369 | 5.80E-16 | 65.50 |
| NID2 | rs77053796 | chr14 | 51900736 | G | T | -0.13 | 0.02 | 0.03 | 35369 | 3.81E-08 | 30.24 |
| NID2 | rs77662174 | chr14 | 52447353 | A | G | -0.14 | 0.02 | 0.05 | 35366 | 2.47E-14 | 58.12 |
| NID2 | rs77792633 | chr14 | 52976430 | G | C | -0.24 | 0.04 | 0.01 | 35367 | 2.81E-11 | 44.30 |
| NID2 | rs77814107 | chr14 | 51920465 | T | C | -0.19 | 0.02 | 0.04 | 35369 | 5.03E-19 | 79.41 |
| NID2 | rs79192483 | chr14 | 52372268 | G | A | -0.27 | 0.04 | 0.01 | 35365 | 9.19E-12 | 46.49 |
| NID2 | rs79564307 | chr14 | 51935085 | T | C | 0.07 | 0.01 | 0.47 | 35368 | 7.50E-18 | 74.07 |
| NID2 | rs8008292 | chr14 | 52301846 | C | T | -0.16 | 0.01 | 0.09 | 35363 | 2.18E-28 | 122.11 |
| NID2 | rs8008348 | chr14 | 52407427 | G | A | 0.07 | 0.01 | 0.34 | 35364 | 4.53E-17 | 70.53 |
| NID2 | rs8014336 | chr14 | 51658789 | C | A | 0.08 | 0.01 | 0.12 | 35369 | 8.95E-10 | 37.54 |
| NID2 | rs802994 | chr14 | 52217487 | A | C | 0.36 | 0.02 | 0.04 | 35366 | 1.25E-73 | 329.45 |
| NID2 | rs803020 | chr14 | 52260914 | T | C | -0.12 | 0.02 | 0.08 | 35367 | 4.39E-14 | 56.98 |
| NID2 | rs873914 | chr14 | 51005127 | T | G | -0.06 | 0.01 | 0.46 | 35374 | 4.50E-12 | 47.90 |
| NID2 | rs941618 | chr14 | 52177949 | T | C | -0.09 | 0.01 | 0.38 | 35366 | 3.89E-25 | 107.26 |
| NID2 | rs9671290 | chr14 | 51757287 | A | G | 0.12 | 0.01 | 0.10 | 35367 | 1.00E-17 | 73.51 |
| HIBCH | rs10451566 | chr2 | 190611751 | T | C | -0.16 | 0.01 | 0.16 | 35368 | 2.69E-48 | 213.24 |
| HIBCH | rs1074247 | chr2 | 190323115 | A | C | 0.13 | 0.02 | 0.04 | 35369 | 7.65E-10 | 37.84 |
| HIBCH | rs10804035 | chr2 | 190380345 | T | C | -0.19 | 0.01 | 0.48 | 35369 | 4.06E-122 | 552.20 |
| HIBCH | rs112590960 | chr2 | 190540324 | A | C | 0.14 | 0.02 | 0.03 | 35367 | 9.93E-09 | 32.85 |
| HIBCH | rs112857039 | chr2 | 190279917 | A | G | 0.18 | 0.01 | 0.09 | 35367 | 8.98E-39 | 169.60 |
| HIBCH | rs113047810 | chr2 | 190538825 | C | T | -0.16 | 0.02 | 0.04 | 35367 | 8.00E-17 | 69.40 |
| HIBCH | rs113226180 | chr2 | 191138646 | G | A | -0.10 | 0.02 | 0.07 | 35361 | 6.41E-10 | 38.19 |
| HIBCH | rs113629677 | chr2 | 190403662 | A | G | 0.09 | 0.01 | 0.16 | 35368 | 2.84E-15 | 62.37 |
| HIBCH | rs114148522 | chr2 | 191028544 | T | G | -0.11 | 0.02 | 0.04 | 35364 | 1.83E-08 | 31.66 |
| HIBCH | rs114635403 | chr2 | 190316871 | C | T | -0.24 | 0.02 | 0.05 | 35369 | 6.13E-37 | 161.20 |
| HIBCH | rs114962169 | chr2 | 190830904 | A | C | 0.11 | 0.02 | 0.07 | 35366 | 6.33E-12 | 47.22 |
| HIBCH | rs116168094 | chr2 | 190862667 | T | C | 0.14 | 0.02 | 0.03 | 35363 | 5.33E-10 | 38.55 |
| HIBCH | rs116275586 | chr2 | 189791328 | A | G | -0.11 | 0.02 | 0.04 | 35362 | 2.70E-08 | 30.91 |
| HIBCH | rs116305095 | chr2 | 190256855 | A | T | -0.17 | 0.03 | 0.02 | 35369 | 1.04E-09 | 37.25 |
| HIBCH | rs12693570 | chr2 | 190374424 | A | C | 0.12 | 0.01 | 0.16 | 35369 | 1.87E-29 | 126.99 |
| HIBCH | rs144775951 | chr2 | 189461823 | G | A | 0.11 | 0.02 | 0.05 | 35359 | 4.38E-09 | 34.44 |
| HIBCH | rs144945343 | chr2 | 190640283 | T | C | -0.19 | 0.02 | 0.04 | 35368 | 1.16E-20 | 86.87 |
| HIBCH | rs149278148 | chr2 | 190146200 | C | T | -0.14 | 0.02 | 0.06 | 35368 | 1.31E-16 | 68.44 |
| HIBCH | rs149836304 | chr2 | 190240785 | A | G | 0.16 | 0.02 | 0.06 | 35369 | 6.72E-23 | 97.06 |
| HIBCH | rs2008950 | chr2 | 190718373 | G | T | -0.11 | 0.01 | 0.48 | 35368 | 1.79E-41 | 181.94 |
| HIBCH | rs2067385 | chr2 | 190342721 | C | T | -0.17 | 0.02 | 0.05 | 35369 | 6.51E-20 | 83.45 |
| HIBCH | rs2117577 | chr2 | 190004858 | G | T | -0.06 | 0.01 | 0.26 | 35360 | 5.36E-10 | 38.54 |
| HIBCH | rs2280232 | chr2 | 190986040 | A | C | -0.07 | 0.01 | 0.24 | 35364 | 2.71E-15 | 62.46 |
| HIBCH | rs2459611 | chr2 | 191074461 | T | C | -0.09 | 0.01 | 0.09 | 35362 | 1.76E-10 | 40.72 |
| HIBCH | rs2690751 | chr2 | 190190540 | A | G | -0.15 | 0.01 | 0.28 | 35363 | 2.98E-61 | 272.63 |
| HIBCH | rs291467 | chr2 | 190322225 | G | A | 0.14 | 0.02 | 0.03 | 35369 | 1.99E-08 | 31.51 |
| HIBCH | rs35178261 | chr2 | 190995286 | C | A | -0.15 | 0.02 | 0.03 | 35364 | 4.16E-10 | 39.03 |
| HIBCH | rs35672585 | chr2 | 191143477 | A | G | -0.07 | 0.01 | 0.16 | 35361 | 1.31E-10 | 41.30 |
| HIBCH | rs35726844 | chr2 | 190843210 | C | G | -0.05 | 0.01 | 0.45 | 35364 | 1.12E-08 | 32.61 |
| HIBCH | rs36015572 | chr2 | 190453427 | C | T | 0.14 | 0.01 | 0.09 | 35366 | 6.08E-25 | 106.38 |
| HIBCH | rs3791778 | chr2 | 189826449 | CCTTTTTTTTTTTTTTGCTA | TCTTTTTTTTTTTTTTGCTA | 0.10 | 0.01 | 0.09 | 35358 | 2.98E-13 | 53.22 |
| HIBCH | rs4853638 | chr2 | 190083959 | C | A | 0.05 | 0.01 | 0.49 | 35361 | 3.01E-10 | 39.67 |
| HIBCH | rs56297515 | chr2 | 190307833 | G | T | 0.14 | 0.01 | 0.11 | 35369 | 2.72E-26 | 112.53 |
| HIBCH | rs62179860 | chr2 | 190892965 | A | G | -0.16 | 0.03 | 0.02 | 35363 | 1.58E-09 | 36.44 |
| HIBCH | rs62184288 | chr2 | 189885390 | G | A | 0.11 | 0.02 | 0.05 | 35359 | 7.91E-10 | 37.78 |
| HIBCH | rs62184386 | chr2 | 190220892 | T | C | 0.24 | 0.01 | 0.48 | 35369 | 1.00E-200 | 941.95 |
| HIBCH | rs62186787 | chr2 | 190071449 | C | T | 0.12 | 0.02 | 0.05 | 35360 | 3.14E-11 | 44.08 |
| HIBCH | rs72903333 | chr2 | 190382656 | A | C | 0.13 | 0.01 | 0.08 | 35369 | 7.44E-20 | 83.19 |
| HIBCH | rs72905384 | chr2 | 189933345 | C | T | 0.11 | 0.02 | 0.05 | 35360 | 3.29E-10 | 39.49 |
| HIBCH | rs72915405 | chr2 | 190174465 | G | A | 0.10 | 0.01 | 0.24 | 35364 | 9.24E-25 | 105.56 |
| HIBCH | rs72916273 | chr2 | 189543786 | G | A | 0.08 | 0.01 | 0.09 | 35357 | 2.11E-09 | 35.87 |
| HIBCH | rs73049729 | chr2 | 190084873 | C | A | -0.14 | 0.01 | 0.09 | 35361 | 2.21E-25 | 108.38 |
| HIBCH | rs751219 | chr2 | 190137321 | G | C | -0.13 | 0.02 | 0.04 | 35368 | 4.78E-10 | 38.76 |
| HIBCH | rs7563737 | chr2 | 189605597 | C | T | -0.07 | 0.01 | 0.13 | 35358 | 2.15E-09 | 35.83 |
| HIBCH | rs75949659 | chr2 | 189399596 | A | C | 0.09 | 0.01 | 0.12 | 35359 | 3.86E-13 | 52.71 |
| HIBCH | rs77124718 | chr2 | 190279161 | T | C | -0.28 | 0.04 | 0.01 | 35369 | 7.39E-14 | 55.96 |
| HIBCH | rs77549534 | chr2 | 189515885 | A | G | -0.19 | 0.03 | 0.02 | 35358 | 1.54E-10 | 40.97 |
| HIBCH | rs77768906 | chr2 | 190069537 | C | T | -0.14 | 0.02 | 0.05 | 35360 | 2.39E-13 | 53.65 |
| HIBCH | rs78925766 | chr2 | 190031517 | A | G | -0.18 | 0.03 | 0.01 | 35361 | 1.49E-08 | 32.07 |
| HIBCH | rs79598899 | chr2 | 190274425 | C | T | -0.22 | 0.01 | 0.09 | 35369 | 2.50E-54 | 240.89 |
| UKB-PPP |  |  |  |  |  |  |  |  |  |  |  |
| Gene names | SNP | Chr | Pos | Effect_allele | Other_allele | Beta | Se | Eaf | Samplesize | Pval | F-statistic |
| TNFSF8 | rs7032773 | 9 | 114883282 | T | C | -0.23 | 0.01 | 0.43 | 33924 | 1.00E-200 | 944.27 |
| TNFSF8 | rs7048418 | 9 | 114622846 | C | T | -0.06 | 0.01 | 0.76 | 33924 | 3.68E-11 | 43.77 |
| HSD17B14 | rs117646304 | 19 | 49202421 | A | G | -0.16 | 0.02 | 0.02 | 33853 | 4.59E-11 | 43.34 |
| HSD17B14 | rs147540796 | 19 | 48794238 | T | C | 0.74 | 0.02 | 0.05 | 33853 | 1.00E-200 | 1552.94 |
| HSD17B14 | rs148552194 | 19 | 48760070 | A | G | -0.23 | 0.03 | 0.02 | 33853 | 1.58E-12 | 49.94 |
| HSD17B14 | rs151024690 | 19 | 48783029 | A | G | -1.44 | 0.02 | 0.04 | 33853 | 1.00E-200 | 5017.77 |
| HSD17B14 | rs77772024 | 19 | 48913817 | A | G | -0.17 | 0.02 | 0.08 | 33853 | 2.08E-30 | 131.33 |
| HSD17B14 | rs8100882 | 19 | 48885205 | T | A | -0.29 | 0.01 | 0.37 | 33853 | 1.00E-200 | 1387.39 |
| PLAU | rs138437684 | 10 | 74012183 | T | C | -0.22 | 0.02 | 0.03 | 33687 | 2.56E-22 | 94.41 |
| PLAU | rs58048583 | 10 | 73908753 | G | T | 0.11 | 0.01 | 0.16 | 33687 | 1.65E-30 | 131.80 |
| TREML2 | rs113582625 | 6 | 41179674 | T | C | 0.22 | 0.02 | 0.04 | 33687 | 2.49E-28 | 121.84 |
| TREML2 | rs116149597 | 6 | 41075409 | A | T | 0.14 | 0.02 | 0.03 | 33687 | 2.10E-10 | 40.37 |
| TREML2 | rs13207171 | 6 | 41198081 | T | C | -0.37 | 0.01 | 0.13 | 33687 | 1.00E-200 | 1131.18 |
| TREML2 | rs145799064 | 6 | 41318320 | C | T | -0.24 | 0.03 | 0.01 | 33687 | 1.26E-12 | 50.38 |
| TREML2 | rs9471493 | 6 | 41186121 | A | G | -0.14 | 0.02 | 0.03 | 33687 | 3.50E-10 | 39.37 |
| MST1 | rs116254545 | 3 | 49471470 | T | C | 0.39 | 0.03 | 0.01 | 33473 | 6.08E-32 | 138.35 |
| MST1 | rs116285936 | 3 | 49158519 | T | C | -1.56 | 0.03 | 0.01 | 33473 | 1.00E-200 | 2390.74 |
| MST1 | rs138138536 | 3 | 49575420 | G | A | -0.69 | 0.04 | 0.01 | 33473 | 1.81E-71 | 319.54 |
| MST1 | rs138838615 | 3 | 48729234 | A | G | -1.24 | 0.02 | 0.04 | 33473 | 1.00E-200 | 3596.80 |
| MST1 | rs140864960 | 3 | 49725193 | A | G | -1.58 | 0.03 | 0.02 | 33473 | 1.00E-200 | 2843.10 |
| MST1 | rs188551769 | 3 | 48686026 | T | G | -0.63 | 0.02 | 0.06 | 33473 | 1.00E-200 | 1565.41 |
| MST1 | rs565220954 | 3 | 49018670 | A | G | 0.21 | 0.03 | 0.01 | 33473 | 2.37E-10 | 40.13 |
| MST1 | rs77462565 | 3 | 50547209 | T | C | 0.32 | 0.02 | 0.06 | 33473 | 7.91E-84 | 376.29 |
| NID2 | rs10148939 | 14 | 51986941 | C | A | -0.39 | 0.01 | 0.14 | 33687 | 1.00E-200 | 1246.37 |
| NID2 | rs1253676 | 14 | 51985856 | A | G | 0.29 | 0.01 | 0.71 | 33687 | 1.00E-200 | 1269.96 |
| NID2 | rs12879081 | 14 | 52210021 | C | A | -0.12 | 0.02 | 0.04 | 33687 | 5.71E-09 | 33.93 |
| NID2 | rs12896877 | 14 | 51765470 | A | G | -0.06 | 0.01 | 0.22 | 33687 | 5.22E-12 | 47.60 |
| NID2 | rs802969 | 14 | 52247393 | T | C | 0.10 | 0.01 | 0.76 | 33687 | 3.85E-27 | 116.41 |
| eQTLs |  |  |  |  |  |  |  |  |  |  |  |
| Gene names | SNP | Chr | Pos | Effect_allele | Other_allele | Beta | Se | Eaf | Samplesize | Pval | F-statistic |
| TNFSF8 | rs10125315 | 9 | 117479509 | T | C | -0.07 | 0.01 | 0.21 | 27641 | 1.67E-12 | 49.84 |
| TNFSF8 | rs10739421 | 9 | 117317872 | T | C | 0.04 | 0.01 | 0.32 | 31562 | 1.14E-05 | 19.26 |
| TNFSF8 | rs10982379 | 9 | 117484812 | G | A | -0.10 | 0.02 | 0.05 | 31237 | 1.60E-07 | 27.46 |
| TNFSF8 | rs10982412 | 9 | 117570856 | A | G | -0.08 | 0.01 | 0.89 | 31683 | 1.01E-10 | 41.79 |
| TNFSF8 | rs10982461 | 9 | 117701408 | A | C | 0.06 | 0.01 | 0.43 | 28464 | 1.79E-13 | 54.22 |
| TNFSF8 | rs112948348 | 9 | 117369926 | T | C | -0.15 | 0.03 | 0.02 | 26794 | 2.91E-07 | 26.31 |
| TNFSF8 | rs113452999 | 9 | 117675501 | G | T | -0.15 | 0.03 | 0.03 | 24544 | 1.07E-08 | 32.71 |
| TNFSF8 | rs116996618 | 9 | 117606250 | T | A | 0.14 | 0.03 | 0.08 | 7972 | 3.03E-06 | 21.79 |
| TNFSF8 | rs117713577 | 9 | 117316464 | T | A | -0.12 | 0.03 | 0.03 | 30264 | 1.96E-06 | 22.64 |
| TNFSF8 | rs118001727 | 9 | 117484989 | T | C | 0.16 | 0.04 | 0.02 | 23692 | 8.86E-06 | 19.74 |
| TNFSF8 | rs141314664 | 9 | 117309486 | G | A | -0.16 | 0.03 | 0.02 | 26155 | 7.41E-06 | 20.08 |
| TNFSF8 | rs144480373 | 9 | 117608388 | G | A | 0.23 | 0.04 | 0.02 | 25812 | 1.08E-10 | 41.68 |
| TNFSF8 | rs145742560 | 9 | 117515346 | T | C | -0.09 | 0.02 | 0.05 | 30782 | 9.51E-07 | 24.02 |
| TNFSF8 | rs150189674 | 9 | 117997490 | G | T | -0.15 | 0.03 | 0.02 | 26276 | 1.05E-05 | 19.41 |
| TNFSF8 | rs185085061 | 9 | 117204663 | C | A | 0.14 | 0.03 | 0.02 | 26082 | 5.18E-06 | 20.77 |
| TNFSF8 | rs190787196 | 9 | 117636135 | A | G | -0.48 | 0.08 | 0.01 | 5526 | 6.41E-09 | 33.69 |
| TNFSF8 | rs2006996 | 9 | 117592638 | C | T | -0.10 | 0.02 | 0.06 | 30971 | 2.61E-09 | 35.45 |
| TNFSF8 | rs2015408 | 9 | 116997994 | T | C | 0.05 | 0.01 | 0.19 | 31676 | 5.77E-06 | 20.56 |
| TNFSF8 | rs2208638 | 9 | 117671525 | G | T | 0.31 | 0.06 | 0.01 | 12462 | 7.52E-08 | 28.92 |
| TNFSF8 | rs35843739 | 9 | 117511629 | C | T | 0.11 | 0.02 | 0.06 | 29661 | 7.16E-10 | 37.98 |
| TNFSF8 | rs4979481 | 9 | 117750232 | C | T | -0.04 | 0.01 | 0.31 | 31556 | 2.13E-07 | 26.91 |
| TNFSF8 | rs55785114 | 9 | 117544314 | T | G | -0.29 | 0.05 | 0.02 | 13345 | 5.63E-09 | 33.95 |
| TNFSF8 | rs56321035 | 9 | 117551489 | T | C | 0.18 | 0.03 | 0.02 | 27801 | 1.52E-11 | 45.50 |
| TNFSF8 | rs62578684 | 9 | 117705336 | A | C | 0.08 | 0.01 | 0.09 | 31355 | 3.69E-09 | 34.78 |
| TNFSF8 | rs7043108 | 9 | 117480466 | A | C | 0.07 | 0.01 | 0.16 | 26701 | 2.61E-09 | 35.45 |
| TNFSF8 | rs725340 | 9 | 117249874 | A | G | -0.08 | 0.01 | 0.08 | 31684 | 1.81E-07 | 27.22 |
| TNFSF8 | rs72754560 | 9 | 117491583 | G | A | 0.17 | 0.03 | 0.02 | 24888 | 2.37E-07 | 26.71 |
| TNFSF8 | rs72756584 | 9 | 117690853 | G | T | -0.22 | 0.03 | 0.02 | 21674 | 5.61E-11 | 42.95 |
| TNFSF8 | rs72760417 | 9 | 117842393 | G | A | -0.13 | 0.03 | 0.02 | 29323 | 4.12E-06 | 21.21 |
| TNFSF8 | rs74612843 | 9 | 117708888 | G | A | -0.09 | 0.02 | 0.06 | 30077 | 8.28E-08 | 28.74 |
| TNFSF8 | rs76315683 | 9 | 117485517 | G | A | -0.23 | 0.04 | 0.06 | 4773 | 2.99E-08 | 30.70 |
| TNFSF8 | rs7847158 | 9 | 117560265 | G | A | -0.15 | 0.01 | 0.27 | 31684 | 5.58E-60 | 266.81 |
| TNFSF8 | rs7871089 | 9 | 117541147 | C | G | -0.06 | 0.01 | 0.23 | 31567 | 4.49E-11 | 43.38 |
| TNFSF8 | rs78872717 | 9 | 117330462 | C | T | -0.07 | 0.02 | 0.07 | 31567 | 5.50E-06 | 20.65 |
| TNFSF8 | rs78979172 | 9 | 117554489 | G | A | -0.31 | 0.03 | 0.02 | 24991 | 2.04E-24 | 103.98 |
| NID2 | rs10146989 | 14 | 52477867 | A | G | -0.04 | 0.01 | 0.28 | 30400 | 1.43E-06 | 23.24 |
| NID2 | rs10148939 | 14 | 52453659 | C | A | -0.11 | 0.01 | 0.13 | 30902 | 4.52E-21 | 88.72 |
| NID2 | rs2101919 | 14 | 52520368 | C | T | -0.04 | 0.01 | 0.24 | 31300 | 1.92E-05 | 18.26 |
| HIBCH | rs10208033 | 2 | 191879417 | C | T | -0.05 | 0.01 | 0.40 | 31568 | 3.86E-10 | 39.18 |
| HIBCH | rs10209516 | 2 | 191061408 | G | A | -0.32 | 0.01 | 0.24 | 31562 | 1.00E-200 | 1215.28 |
| HIBCH | rs10804034 | 2 | 191244826 | T | C | 0.18 | 0.01 | 0.65 | 31565 | 3.88E-100 | 451.21 |
| HIBCH | rs112185050 | 2 | 190843412 | G | A | -0.14 | 0.03 | 0.03 | 19957 | 7.42E-06 | 20.08 |
| HIBCH | rs113157103 | 2 | 191271570 | A | G | 0.23 | 0.04 | 0.02 | 16094 | 1.04E-07 | 28.29 |
| HIBCH | rs113550116 | 2 | 191232289 | C | T | 0.31 | 0.07 | 0.02 | 5218 | 9.05E-06 | 19.70 |
| HIBCH | rs114090683 | 2 | 190244325 | C | T | -0.13 | 0.02 | 0.03 | 25173 | 2.08E-07 | 26.96 |
| HIBCH | rs114137338 | 2 | 191133275 | A | G | 0.21 | 0.02 | 0.04 | 27069 | 8.95E-22 | 91.93 |
| HIBCH | rs114300649 | 2 | 191089610 | A | C | -0.41 | 0.05 | 0.01 | 17092 | 5.98E-19 | 79.06 |
| HIBCH | rs114428434 | 2 | 191567537 | C | T | 0.14 | 0.03 | 0.02 | 22064 | 9.70E-06 | 19.57 |
| HIBCH | rs114488159 | 2 | 191050116 | A | G | 0.21 | 0.03 | 0.02 | 24429 | 3.14E-12 | 48.60 |
| HIBCH | rs114977554 | 2 | 191052513 | A | G | -0.17 | 0.04 | 0.02 | 22591 | 5.15E-06 | 20.78 |
| HIBCH | rs115221611 | 2 | 191479670 | A | G | -0.21 | 0.04 | 0.02 | 25252 | 5.86E-09 | 33.88 |
| HIBCH | rs115499558 | 2 | 191661741 | G | A | 0.12 | 0.02 | 0.04 | 28308 | 2.83E-08 | 30.82 |
| HIBCH | rs116678411 | 2 | 191103442 | A | G | -0.31 | 0.03 | 0.02 | 25850 | 7.35E-22 | 92.32 |
| HIBCH | rs11676165 | 2 | 192042056 | T | C | 0.10 | 0.02 | 0.04 | 24950 | 1.13E-05 | 19.27 |
| HIBCH | rs11685878 | 2 | 192009455 | T | C | -0.05 | 0.01 | 0.38 | 31355 | 6.14E-09 | 33.78 |
| HIBCH | rs117967769 | 2 | 190161061 | T | C | -0.14 | 0.03 | 0.02 | 23346 | 4.81E-06 | 20.91 |
| HIBCH | rs11889341 | 2 | 191943742 | T | C | -0.05 | 0.01 | 0.22 | 31684 | 4.40E-07 | 25.51 |
| HIBCH | rs1263142 | 2 | 191592857 | T | C | -0.07 | 0.01 | 0.16 | 31684 | 1.08E-11 | 46.18 |
| HIBCH | rs12693570 | 2 | 191239150 | A | C | 0.12 | 0.01 | 0.15 | 29992 | 4.70E-26 | 111.45 |
| HIBCH | rs13024726 | 2 | 190492804 | A | G | 0.05 | 0.01 | 0.17 | 31355 | 1.74E-05 | 18.46 |
| HIBCH | rs140724771 | 2 | 191250672 | A | G | -0.24 | 0.02 | 0.03 | 29741 | 5.56E-24 | 101.99 |
| HIBCH | rs140840179 | 2 | 191044686 | T | C | 0.15 | 0.03 | 0.02 | 22697 | 1.36E-06 | 23.33 |
| HIBCH | rs141699035 | 2 | 191093099 | A | G | 0.28 | 0.05 | 0.01 | 18045 | 8.37E-09 | 33.18 |
| HIBCH | rs143457746 | 2 | 191088802 | A | G | -0.38 | 0.04 | 0.01 | 20258 | 1.39E-19 | 81.95 |
| HIBCH | rs144945343 | 2 | 191505009 | T | C | -0.22 | 0.03 | 0.02 | 28439 | 8.61E-16 | 64.72 |
| HIBCH | rs146003777 | 2 | 191091177 | T | C | 0.24 | 0.04 | 0.02 | 23727 | 2.85E-11 | 44.27 |
| HIBCH | rs147365564 | 2 | 191275527 | C | T | -0.33 | 0.03 | 0.02 | 22199 | 1.96E-25 | 108.61 |
| HIBCH | rs148125398 | 2 | 191143785 | A | G | -0.30 | 0.03 | 0.02 | 25364 | 1.37E-24 | 104.77 |
| HIBCH | rs149789304 | 2 | 190507240 | A | G | -0.15 | 0.03 | 0.02 | 24857 | 6.90E-06 | 20.22 |
| HIBCH | rs150035553 | 2 | 191299116 | G | A | 0.13 | 0.03 | 0.03 | 29006 | 4.16E-07 | 25.62 |
| HIBCH | rs150330068 | 2 | 191130572 | A | G | -0.28 | 0.04 | 0.01 | 23514 | 2.66E-13 | 53.44 |
| HIBCH | rs17736581 | 2 | 191111045 | G | A | 0.16 | 0.01 | 0.11 | 30867 | 2.04E-33 | 145.10 |
| HIBCH | rs17769459 | 2 | 191993548 | T | C | -0.14 | 0.02 | 0.04 | 25173 | 1.72E-11 | 45.26 |
| HIBCH | rs184888371 | 2 | 191104241 | C | T | 0.34 | 0.06 | 0.01 | 10217 | 7.15E-09 | 33.49 |
| HIBCH | rs184952365 | 2 | 191089878 | T | C | 0.26 | 0.03 | 0.02 | 24347 | 6.40E-19 | 78.94 |
| HIBCH | rs2066797 | 2 | 191854423 | C | T | -0.08 | 0.02 | 0.05 | 31684 | 9.71E-06 | 19.57 |
| HIBCH | rs2690751 | 2 | 191055266 | A | G | -0.17 | 0.01 | 0.26 | 31684 | 1.05E-84 | 380.32 |
| HIBCH | rs56101840 | 2 | 191281032 | C | A | 0.10 | 0.02 | 0.06 | 31563 | 3.47E-10 | 39.39 |
| HIBCH | rs59924974 | 2 | 191186368 | G | A | -0.12 | 0.03 | 0.02 | 30617 | 1.52E-05 | 18.71 |
| HIBCH | rs62182902 | 2 | 191502767 | C | G | -0.13 | 0.01 | 0.77 | 31564 | 9.65E-45 | 196.94 |
| HIBCH | rs62184383 | 2 | 191061603 | C | A | -0.39 | 0.03 | 0.02 | 24861 | 5.18E-38 | 166.12 |
| HIBCH | rs62187111 | 2 | 190374084 | T | C | 0.12 | 0.02 | 0.03 | 28439 | 4.36E-07 | 25.53 |
| HIBCH | rs66911790 | 2 | 191904670 | C | G | 0.07 | 0.01 | 0.10 | 30157 | 2.28E-07 | 26.78 |
| HIBCH | rs6750727 | 2 | 190993716 | T | G | -0.08 | 0.02 | 0.05 | 31355 | 4.85E-06 | 20.89 |
| HIBCH | rs6751901 | 2 | 191039920 | C | T | -0.05 | 0.01 | 0.17 | 28588 | 2.59E-06 | 22.09 |
| HIBCH | rs72905384 | 2 | 190798071 | C | T | 0.11 | 0.02 | 0.05 | 26835 | 2.46E-08 | 31.09 |
| HIBCH | rs72907244 | 2 | 191775183 | G | A | 0.18 | 0.03 | 0.02 | 25720 | 4.62E-10 | 38.83 |
| HIBCH | rs73978974 | 2 | 191030714 | C | T | 0.08 | 0.01 | 0.18 | 31569 | 1.09E-15 | 64.26 |
| HIBCH | rs74654840 | 2 | 191071369 | C | T | 0.28 | 0.05 | 0.01 | 12505 | 2.45E-07 | 26.64 |
| HIBCH | rs75482720 | 2 | 190961468 | C | T | 0.12 | 0.02 | 0.03 | 29471 | 2.21E-06 | 22.41 |
| HIBCH | rs7557586 | 2 | 191264497 | T | A | -0.05 | 0.01 | 0.24 | 31568 | 2.39E-07 | 26.68 |
| HIBCH | rs7586853 | 2 | 190137108 | T | A | 0.05 | 0.01 | 0.80 | 31569 | 5.95E-07 | 24.93 |
| HIBCH | rs7605252 | 2 | 191041290 | G | A | -0.12 | 0.02 | 0.03 | 27251 | 5.39E-07 | 25.12 |
| HIBCH | rs76626815 | 2 | 190883750 | A | G | -0.25 | 0.04 | 0.02 | 19807 | 6.70E-11 | 42.60 |
| HIBCH | rs77124718 | 2 | 191143887 | T | C | -0.50 | 0.05 | 0.04 | 4888 | 5.95E-21 | 88.15 |
| HIBCH | rs77586566 | 2 | 190955493 | T | C | 0.09 | 0.02 | 0.07 | 31355 | 9.97E-08 | 28.38 |
| HIBCH | rs77811961 | 2 | 190786628 | T | G | -0.14 | 0.03 | 0.03 | 24013 | 3.70E-07 | 25.84 |
| HIBCH | rs785234 | 2 | 190957240 | C | G | 0.07 | 0.01 | 0.29 | 31567 | 8.08E-16 | 64.85 |
| HIBCH | rs785242 | 2 | 190947260 | A | G | -0.04 | 0.01 | 0.42 | 31684 | 1.16E-05 | 19.23 |
| HIBCH | rs79727714 | 2 | 190946134 | A | C | 0.06 | 0.01 | 0.09 | 31228 | 1.06E-05 | 19.40 |
| HSD17B14 | rs10408884 | 19 | 49315046 | T | G | 0.05 | 0.01 | 0.69 | 27215 | 2.99E-09 | 35.19 |
| HSD17B14 | rs114122193 | 19 | 49337647 | T | C | -0.22 | 0.01 | 0.11 | 25822 | 3.16E-57 | 254.17 |
| HSD17B14 | rs116922356 | 19 | 49315136 | T | C | 0.08 | 0.02 | 0.09 | 25902 | 2.11E-07 | 26.93 |
| HSD17B14 | rs12459187 | 19 | 49336881 | T | C | -0.14 | 0.01 | 0.10 | 27156 | 8.79E-22 | 91.96 |
| HSD17B14 | rs12981368 | 19 | 49419018 | G | A | 0.04 | 0.01 | 0.28 | 26130 | 4.18E-06 | 21.18 |
| HSD17B14 | rs150368897 | 19 | 49410262 | T | C | -0.22 | 0.05 | 0.02 | 10841 | 1.29E-05 | 19.02 |
| HSD17B14 | rs151024690 | 19 | 49286286 | A | G | 0.11 | 0.02 | 0.04 | 25249 | 9.62E-07 | 24.00 |
| HSD17B14 | rs2387579 | 19 | 49416456 | G | A | -0.08 | 0.01 | 0.42 | 19770 | 1.05E-16 | 68.86 |
| HSD17B14 | rs281407 | 19 | 49233335 | A | G | 0.04 | 0.01 | 0.65 | 31684 | 1.38E-05 | 18.90 |
| HSD17B14 | rs35815377 | 19 | 49706655 | C | T | 0.07 | 0.02 | 0.10 | 23025 | 1.89E-05 | 18.29 |
| HSD17B14 | rs368654 | 19 | 49116555 | A | G | -0.04 | 0.01 | 0.24 | 29670 | 3.29E-06 | 21.64 |
| HSD17B14 | rs3786733 | 19 | 49408303 | C | T | 0.11 | 0.02 | 0.07 | 18187 | 3.51E-08 | 30.40 |
| HSD17B14 | rs4645896 | 19 | 49461834 | A | G | -0.09 | 0.02 | 0.06 | 19856 | 5.76E-06 | 20.56 |
| HSD17B14 | rs4802503 | 19 | 49279628 | G | A | 0.07 | 0.01 | 0.56 | 23209 | 2.81E-14 | 57.86 |
| HSD17B14 | rs518388 | 19 | 49285487 | C | T | 0.06 | 0.01 | 0.22 | 29738 | 3.07E-09 | 35.14 |
| HSD17B14 | rs560343 | 19 | 49273784 | G | A | 0.05 | 0.01 | 0.29 | 26514 | 1.49E-06 | 23.16 |
| HSD17B14 | rs623520 | 19 | 49369943 | G | A | -0.21 | 0.01 | 0.32 | 31566 | 5.92E-132 | 597.44 |
| HSD17B14 | rs7260346 | 19 | 49384786 | A | C | 0.06 | 0.01 | 0.08 | 31567 | 1.44E-05 | 18.82 |
| HSD17B14 | rs73061670 | 19 | 49387489 | A | G | 0.19 | 0.04 | 0.02 | 18867 | 4.28E-06 | 21.13 |
| HSD17B14 | rs78120516 | 19 | 49411198 | G | T | 0.10 | 0.02 | 0.05 | 18697 | 1.58E-05 | 18.64 |
| HSD17B14 | rs78298080 | 19 | 49094470 | C | T | 0.07 | 0.02 | 0.09 | 23095 | 9.65E-06 | 19.58 |
| HSD17B14 | rs79187430 | 19 | 49359958 | A | G | 0.17 | 0.01 | 0.12 | 26145 | 4.41E-37 | 161.86 |
| HSD17B14 | rs79390987 | 19 | 49264232 | C | T | 0.07 | 0.02 | 0.07 | 26497 | 1.10E-05 | 19.33 |
| XXYLT1 | rs10933693 | 3 | 194767164 | G | T | 0.06 | 0.01 | 0.12 | 25357 | 2.74E-06 | 21.99 |
| XXYLT1 | rs111620138 | 3 | 194780324 | C | T | 0.13 | 0.03 | 0.03 | 21588 | 1.24E-05 | 19.10 |
| XXYLT1 | rs114044674 | 3 | 194945481 | T | C | 0.13 | 0.03 | 0.03 | 23838 | 3.49E-07 | 25.95 |
| XXYLT1 | rs115986307 | 3 | 194795210 | A | G | 0.21 | 0.04 | 0.02 | 16900 | 5.38E-08 | 29.57 |
| XXYLT1 | rs116179639 | 3 | 194721224 | C | T | 0.25 | 0.05 | 0.02 | 9998 | 3.80E-07 | 25.79 |
| XXYLT1 | rs116420460 | 3 | 195074002 | T | C | 0.18 | 0.03 | 0.03 | 15697 | 2.91E-08 | 30.76 |
| XXYLT1 | rs116833683 | 3 | 194797921 | A | G | -0.23 | 0.05 | 0.01 | 13295 | 1.79E-05 | 18.40 |
| XXYLT1 | rs11922145 | 3 | 195520807 | T | A | -0.07 | 0.01 | 0.12 | 23749 | 8.01E-07 | 24.35 |
| XXYLT1 | rs11925666 | 3 | 195518660 | G | A | -0.05 | 0.01 | 0.20 | 25243 | 1.89E-05 | 18.30 |
| XXYLT1 | rs13066800 | 3 | 195032392 | G | A | 0.17 | 0.02 | 0.05 | 18098 | 8.56E-13 | 51.14 |
| XXYLT1 | rs140680425 | 3 | 194899792 | T | C | 0.23 | 0.03 | 0.02 | 20897 | 1.31E-13 | 54.83 |
| XXYLT1 | rs145097443 | 3 | 195270328 | C | T | 0.14 | 0.03 | 0.04 | 14588 | 5.02E-06 | 20.83 |
| XXYLT1 | rs1464504 | 3 | 195300284 | A | G | 0.05 | 0.01 | 0.70 | 20484 | 1.78E-05 | 18.42 |
| XXYLT1 | rs148087002 | 3 | 194779008 | T | C | -0.22 | 0.05 | 0.01 | 13140 | 1.62E-05 | 18.59 |
| XXYLT1 | rs149597644 | 3 | 194758037 | T | C | 0.31 | 0.06 | 0.02 | 9451 | 2.96E-08 | 30.73 |
| XXYLT1 | rs2642390 | 3 | 194798161 | G | A | 0.06 | 0.01 | 0.79 | 25574 | 2.91E-07 | 26.31 |
| XXYLT1 | rs2676903 | 3 | 194758864 | G | A | 0.11 | 0.02 | 0.91 | 21939 | 1.34E-10 | 41.25 |
| XXYLT1 | rs2720932 | 3 | 194775336 | T | C | 0.07 | 0.02 | 0.08 | 25690 | 1.64E-05 | 18.57 |
| XXYLT1 | rs2720956 | 3 | 194770579 | G | T | 0.05 | 0.01 | 0.72 | 24622 | 8.84E-07 | 24.16 |
| XXYLT1 | rs28535132 | 3 | 194819390 | C | T | 0.05 | 0.01 | 0.80 | 25361 | 1.63E-05 | 18.58 |
| XXYLT1 | rs4677820 | 3 | 194982437 | C | T | -0.06 | 0.01 | 0.20 | 25242 | 2.40E-07 | 26.68 |
| XXYLT1 | rs56170710 | 3 | 194899060 | A | C | 0.11 | 0.02 | 0.04 | 24621 | 2.82E-07 | 26.37 |
| XXYLT1 | rs59834989 | 3 | 194888873 | T | C | -0.19 | 0.02 | 0.95 | 25360 | 1.10E-21 | 91.53 |
| XXYLT1 | rs59954466 | 3 | 195550412 | C | T | 0.08 | 0.02 | 0.08 | 22156 | 8.61E-06 | 19.80 |
| XXYLT1 | rs60677730 | 3 | 194931112 | C | T | -0.13 | 0.03 | 0.03 | 24976 | 2.04E-07 | 26.99 |
| XXYLT1 | rs62283272 | 3 | 195562917 | G | C | 0.12 | 0.03 | 0.04 | 20348 | 2.27E-06 | 22.35 |
| XXYLT1 | rs62284390 | 3 | 195648485 | T | C | -0.14 | 0.03 | 0.03 | 21094 | 1.88E-06 | 22.71 |
| XXYLT1 | rs62287064 | 3 | 195019509 | T | C | -0.09 | 0.02 | 0.12 | 20917 | 1.78E-08 | 31.72 |
| XXYLT1 | rs62292506 | 3 | 194739822 | A | C | 0.06 | 0.01 | 0.13 | 25361 | 4.91E-06 | 20.87 |
| XXYLT1 | rs6780281 | 3 | 194784551 | A | G | 0.09 | 0.01 | 0.21 | 25243 | 4.33E-17 | 70.62 |
| XXYLT1 | rs72611104 | 3 | 195009583 | A | G | -0.09 | 0.02 | 0.94 | 21743 | 4.56E-06 | 21.01 |
| XXYLT1 | rs73196126 | 3 | 195246825 | A | G | -0.27 | 0.06 | 0.02 | 7126 | 7.19E-06 | 20.14 |
| XXYLT1 | rs73205723 | 3 | 195485854 | A | G | 0.35 | 0.05 | 0.02 | 11117 | 8.22E-14 | 55.75 |
| XXYLT1 | rs73206696 | 3 | 195065978 | G | A | -0.32 | 0.03 | 0.02 | 21784 | 2.28E-24 | 103.75 |
| XXYLT1 | rs75602459 | 3 | 195197272 | A | G | -0.17 | 0.03 | 0.03 | 13786 | 5.93E-07 | 24.93 |
| XXYLT1 | rs7613798 | 3 | 194778285 | G | C | 0.13 | 0.03 | 0.54 | 2157 | 1.23E-05 | 19.09 |
| XXYLT1 | rs7625484 | 3 | 195035537 | C | A | 0.11 | 0.01 | 0.19 | 24908 | 1.77E-22 | 95.14 |
| XXYLT1 | rs76326750 | 3 | 194856978 | T | C | -0.21 | 0.05 | 0.02 | 13511 | 1.40E-05 | 18.86 |
| XXYLT1 | rs9859891 | 3 | 194826872 | C | T | 0.05 | 0.01 | 0.28 | 25564 | 2.50E-06 | 22.16 |
| XXYLT1 | rs9870813 | 3 | 194924130 | C | T | 0.06 | 0.01 | 0.41 | 25690 | 1.29E-12 | 50.35 |
| PLAU | rs11000814 | 10 | 75700056 | T | C | 0.09 | 0.01 | 0.21 | 25788 | 1.73E-17 | 72.42 |
| PLAU | rs11000851 | 10 | 75781960 | G | T | 0.05 | 0.01 | 0.82 | 31672 | 1.03E-07 | 28.32 |
| PLAU | rs117172379 | 10 | 75696966 | T | C | 0.55 | 0.03 | 0.02 | 24839 | 4.87E-65 | 290.02 |
| PLAU | rs138918631 | 10 | 75240778 | A | C | 0.19 | 0.04 | 0.02 | 14937 | 4.47E-06 | 21.05 |
| PLAU | rs143680871 | 10 | 74678994 | C | A | 0.24 | 0.03 | 0.03 | 24752 | 1.25E-20 | 86.72 |
| PLAU | rs2227581 | 10 | 75671875 | A | G | -0.12 | 0.02 | 0.07 | 18211 | 2.01E-09 | 35.96 |
| PLAU | rs34262333 | 10 | 76649033 | C | T | 0.19 | 0.02 | 0.05 | 24981 | 4.05E-21 | 88.95 |
| PLAU | rs35601073 | 10 | 75900464 | G | A | 0.07 | 0.01 | 0.14 | 26669 | 1.21E-07 | 28.00 |
| PLAU | rs3870401 | 10 | 75473704 | C | G | 0.14 | 0.01 | 0.75 | 27132 | 3.85E-44 | 194.19 |
| PLAU | rs4746142 | 10 | 75430070 | T | C | 0.09 | 0.02 | 0.10 | 14105 | 1.92E-05 | 18.27 |
| PLAU | rs59035627 | 10 | 74713046 | G | T | -0.09 | 0.02 | 0.06 | 27069 | 2.22E-07 | 26.83 |
| PLAU | rs7090105 | 10 | 75131545 | T | C | 0.10 | 0.01 | 0.71 | 28474 | 3.34E-26 | 112.12 |
| PLAU | rs7095447 | 10 | 75119899 | A | G | -0.08 | 0.01 | 0.17 | 25304 | 4.75E-11 | 43.27 |
| PLAU | rs71471654 | 10 | 75732341 | G | T | 0.16 | 0.03 | 0.03 | 23570 | 1.95E-08 | 31.54 |
| PLAU | rs72820527 | 10 | 76463300 | A | C | -0.08 | 0.02 | 0.08 | 23876 | 8.03E-07 | 24.35 |
| PLAU | rs77079999 | 10 | 75201067 | G | A | 0.22 | 0.04 | 0.03 | 11192 | 2.11E-08 | 31.38 |
| PLAU | rs78077522 | 10 | 74698394 | C | T | 0.12 | 0.02 | 0.05 | 25444 | 3.35E-08 | 30.49 |
| PLAU | rs79014227 | 10 | 75855980 | T | C | -0.08 | 0.02 | 0.06 | 23939 | 1.97E-05 | 18.22 |
| PLAU | rs7924176 | 10 | 76295789 | G | A | -0.05 | 0.01 | 0.59 | 31684 | 1.07E-08 | 32.71 |
| MST1 | rs111327216 | 3 | 49525218 | T | C | -0.12 | 0.03 | 0.13 | 6408 | 9.98E-06 | 19.51 |
| MST1 | rs111494817 | 3 | 49120006 | C | T | -0.17 | 0.04 | 0.94 | 6412 | 2.08E-06 | 22.51 |
| MST1 | rs12634780 | 3 | 50043341 | A | T | -0.06 | 0.02 | 0.40 | 9176 | 1.64E-05 | 18.56 |
| MST1 | rs12635454 | 3 | 48763656 | A | G | 0.16 | 0.03 | 0.09 | 6415 | 2.50E-07 | 26.59 |
| MST1 | rs138838615 | 3 | 48766667 | A | G | 0.46 | 0.06 | 0.03 | 5258 | 2.35E-14 | 58.19 |
| MST1 | rs146231194 | 3 | 49651603 | T | C | -0.19 | 0.03 | 0.12 | 6409 | 1.13E-12 | 50.58 |
| MST1 | rs188683902 | 3 | 48754429 | C | T | 0.35 | 0.07 | 0.02 | 5236 | 8.24E-07 | 24.29 |
| MST1 | rs74673105 | 3 | 48916980 | C | A | -0.44 | 0.10 | 0.01 | 3645 | 8.51E-06 | 19.81 |
| MST1 | rs76037525 | 3 | 48924190 | T | G | -0.26 | 0.05 | 0.04 | 5592 | 4.15E-07 | 25.61 |
| MST1 | rs79448908 | 3 | 49169782 | A | G | 0.27 | 0.06 | 0.03 | 6417 | 1.78E-06 | 22.82 |
| MST1 | rs9811027 | 3 | 48725014 | C | G | 0.12 | 0.02 | 0.11 | 9187 | 3.83E-07 | 25.78 |
| TREML2 | rs115706695 | 6 | 40619865 | A | C | -0.19 | 0.03 | 0.02 | 28161 | 3.40E-10 | 39.42 |
| TREML2 | rs116677682 | 6 | 41312534 | T | C | -0.17 | 0.02 | 0.03 | 27940 | 5.24E-12 | 47.59 |
| TREML2 | rs11757145 | 6 | 41313373 | C | T | 0.06 | 0.01 | 0.11 | 31569 | 8.02E-06 | 19.93 |
| TREML2 | rs11759347 | 6 | 41167097 | A | C | 0.08 | 0.01 | 0.23 | 31339 | 4.84E-17 | 70.40 |
| TREML2 | rs13218943 | 6 | 41321645 | T | C | 0.08 | 0.02 | 0.07 | 28673 | 5.19E-07 | 25.19 |
| TREML2 | rs147373305 | 6 | 40861131 | A | G | 0.21 | 0.04 | 0.02 | 26192 | 1.63E-09 | 36.37 |
| TREML2 | rs1534994 | 6 | 40954700 | T | C | -0.18 | 0.03 | 0.03 | 29003 | 1.26E-12 | 50.38 |
| TREML2 | rs1535582 | 6 | 41364255 | C | T | 0.04 | 0.01 | 0.41 | 31684 | 1.52E-06 | 23.13 |
| TREML2 | rs16894387 | 6 | 41240171 | C | A | 0.11 | 0.01 | 0.14 | 31556 | 2.54E-21 | 89.87 |
| TREML2 | rs191348366 | 6 | 41272871 | T | C | 0.23 | 0.04 | 0.02 | 18336 | 1.31E-08 | 32.32 |
| TREML2 | rs1983799 | 6 | 41382998 | A | T | -0.05 | 0.01 | 0.84 | 30902 | 6.22E-07 | 24.84 |
| TREML2 | rs2008287 | 6 | 41217258 | C | T | -0.07 | 0.01 | 0.14 | 30077 | 1.63E-09 | 36.38 |
| TREML2 | rs2395751 | 6 | 41169428 | T | G | -0.09 | 0.01 | 0.17 | 31684 | 3.14E-16 | 66.71 |
| TREML2 | rs3807040 | 6 | 41170895 | T | G | 0.06 | 0.01 | 0.08 | 31568 | 1.96E-05 | 18.23 |
| TREML2 | rs3807041 | 6 | 41170670 | T | C | -0.05 | 0.01 | 0.35 | 31565 | 5.47E-10 | 38.50 |
| TREML2 | rs4714409 | 6 | 40927795 | A | G | -0.07 | 0.01 | 0.09 | 31684 | 5.26E-07 | 25.16 |
| TREML2 | rs56177516 | 6 | 41334319 | C | G | -0.04 | 0.01 | 0.60 | 30077 | 1.43E-05 | 18.83 |
| TREML2 | rs56302558 | 6 | 41165271 | A | G | 0.16 | 0.01 | 0.19 | 30192 | 2.92E-54 | 240.58 |
| TREML2 | rs61738668 | 6 | 41191901 | A | G | -0.10 | 0.02 | 0.03 | 28112 | 9.16E-06 | 19.68 |
| TREML2 | rs620922 | 6 | 41345565 | T | G | -0.10 | 0.02 | 0.03 | 29951 | 1.10E-05 | 19.33 |
| TREML2 | rs6458191 | 6 | 40927983 | C | A | -0.03 | 0.01 | 0.48 | 31569 | 1.66E-05 | 18.54 |
| TREML2 | rs6914090 | 6 | 41242972 | C | T | 0.05 | 0.01 | 0.53 | 31555 | 2.54E-11 | 44.50 |
| TREML2 | rs6917512 | 6 | 40828285 | T | C | 0.04 | 0.01 | 0.53 | 31684 | 3.37E-06 | 21.59 |
| TREML2 | rs75397525 | 6 | 41016368 | T | C | -0.11 | 0.02 | 0.04 | 29143 | 2.32E-06 | 22.31 |
| TREML2 | rs75546655 | 6 | 40931950 | A | G | -0.26 | 0.03 | 0.02 | 26133 | 2.39E-15 | 62.71 |
| TREML2 | rs75976212 | 6 | 41167732 | C | T | -0.09 | 0.02 | 0.96 | 31236 | 1.00E-05 | 19.51 |
| TREML2 | rs76271738 | 6 | 40876663 | G | A | 0.09 | 0.01 | 0.87 | 31558 | 1.24E-13 | 54.93 |
| TREML2 | rs76292476 | 6 | 40976547 | A | G | 0.15 | 0.03 | 0.02 | 28466 | 6.84E-08 | 29.11 |
| TREML2 | rs76474117 | 6 | 40779272 | A | G | -0.08 | 0.02 | 0.07 | 31230 | 1.43E-06 | 23.24 |
| TREML2 | rs77280640 | 6 | 40929244 | C | T | 0.17 | 0.03 | 0.02 | 27522 | 1.41E-07 | 27.71 |
| TREML2 | rs77303168 | 6 | 41362440 | T | C | 0.10 | 0.02 | 0.03 | 28740 | 1.91E-05 | 18.28 |
| TREML2 | rs9296352 | 6 | 41052874 | C | A | -0.08 | 0.01 | 0.14 | 31185 | 1.97E-12 | 49.51 |
| TREML2 | rs9369253 | 6 | 40986538 | G | A | -0.05 | 0.01 | 0.24 | 31568 | 1.11E-06 | 23.72 |
| TREML2 | rs9471531 | 6 | 41242200 | G | A | 0.11 | 0.01 | 0.64 | 31355 | 5.39E-42 | 184.36 |
| TREML2 | rs9471553 | 6 | 41290704 | C | T | -0.05 | 0.01 | 0.21 | 31684 | 3.70E-06 | 21.41 |

Chr,Chromosome number;Pos,position;Effect_allele,effect allele.Other_allele,other allele.EAF,effect allele frequency.Beta,beta regression coefficient.Se,standard error. Pval, P value.F- statistic, F statistic.

**Appendix Table 3. Results of MR analysis of plasma proteins and OC**

| deCODE | | |  | | | |  | | |  | | | |  | | | UKB-PPP | | | |  | | |  | | | |  | | |
| --- | --- | --- | --- | --- | --- | --- | --- | --- | --- | --- | --- | --- | --- | --- | --- | --- | --- | --- | --- | --- | --- | --- | --- | --- | --- | --- | --- | --- | --- | --- |
| Exposure | Gene names | Method | | nsnp | Beta | Se | | Pval | FDR_Pval | | OR（95％CI） | Egger_intercept | Pleiotropy pval | | Q_pval | MR Egger Q_pval | | Method | nsnp | Beta | | Se | Pval | | OR（95％CI） | Egger_intercept | Pleiotropy pval | | Q_pval | MR Egger Q_pval |
| CD30 Ligand | TNFSF8 | Inverse variance weighted | | 10 | 0.84 | 0.15 | | 2.49E-08 | 1.69E-04 | | 2.31(1.72-3.11) | 0.04 | 0.25 | | 0.51 | 0.57 | | Inverse variance weighted | 2 | 0.43 | | 0.13 | 8.25E-04 | | 1.53(1.19-1.96) | NA | NA | | 0.64 | NA |
| uPA | PLAU | Inverse variance weighted | | 24 | -0.33 | 0.07 | | 4.92E-06 | 0.01 | | 0.72(0.62-0.83) | -0.07 | 0.02 | | 0.73 | 0.96 | | Inverse variance weighted | 2 | 0.25 | | 0.43 | 0.56 | | 1.28(0.55-2.96) | NA | NA | | 0.32 | NA |
| TREML2 | TREML2 | Inverse variance weighted | | 94 | -0.15 | 0.04 | | 2.92E-05 | 0.05 | | 0.86(0.80-0.92) | -0.01 | 0.18 | | 0.13 | 0.14 | | Inverse variance weighted | 5 | 0.17 | | 0.17 | 0.32 | | 1.19(0.84-1.67) | -0.04 | 0.75 | | 0.65 | 0.50 |
| MSP | MST1 | Inverse variance weighted | | 44 | 0.09 | 0.02 | | 4.40E-05 | 0.05 | | 1.09(1.05-1.14) | -0.02 | 0.51 | | 0.39 | 0.37 | | Inverse variance weighted | 8 | 0.07 | | 0.04 | 0.07 | | 1.08(1.00-1.17) | -0.03 | 0.76 | | 0.62 | 0.51 |
| NID2 | NID2 | Inverse variance weighted | | 87 | 0.17 | 0.03 | | 5.27E-07 | 1.79E-03 | | 1.19(1.11-1.27) | 0.02 | 0.06 | | 0.98 | 0.99 | | Inverse variance weighted | 5 | 0.04 | | 0.10 | 0.66 | | 1.04(0.87-1.26) | 0.04 | 0.48 | | 0.16 | 0.15 |
| HIBCH | HIBCH | Inverse variance weighted | | 48 | -0.28 | 0.07 | | 4.13E-05 | 0.05 | | 0.76(0.66-0.86) | -0.01 | 0.56 | | 0.17 | 0.16 | | NA | NA | NA | | NA | NA | | NA | NA | NA | | NA | NA |
| Additional findings |  |  | |  |  |  | |  |  | |  |  |  | |  |  | |  |  |  | |  |  | |  |  |  | |  |  |
| XXLT1 | XXYLT1 | Inverse variance weighted | | 32 | 0.29 | 0.08 | | 1.14E-04 | 0.09 | | 1.34(1.15-1.55) | 0.04 | 0.13 | | 0.42 | 0.49 | | NA | NA | NA | | NA | NA | | NA | NA | NA | | NA | NA |
| DHB14 | HSD17B14 | Inverse variance weighted | | 4 | 0.69 | 0.34 | | 0.04 | 0.98 | | 2.00(1.02-3.91) | -0.09 | 0.26 | | 0.21 | 0.37 | | Inverse variance weighted | 6 | 0.07 | | 0.05 | 0.14 | | 1.07(0.98-1.18) | -0.08 | 0.11 | | 0.48 | 0.99 |

MR,Mendelian Randomizatio;OC,Oral cancer;SNP,Single nucleotide polymorphism;Beta,beta regression coefficient;Se,standard error;Pval,P-value; FDR,False Discovery Rate;OR,Odds ratio;(95%CI), 95%Confidence interval;Pleiotropy, Pleiotropy Test; Q_pval, horizontal pleiotropy P-value for Q-test.

Appendix Table 4. Results of MR analysis of OC and plasma proteins

| Exposure | Outcome | Method | nsnp | Beta | Se | Pval | Heterogeneity(Pval) | | Pleiotropy(Pval) |
| --- | --- | --- | --- | --- | --- | --- | --- | --- | --- |
| Oral Cancer | TNFSF8 | MR Egger | 3 | -0.010 | 0.459 | 0.986 | MR Egger | 0.797 | 0.990 |
| Oral Cancer | TNFSF8 | Weighted median | 3 | -0.007 | 0.029 | 0.817 | Inverse variance weighted | 0.967 |  |
| Oral Cancer | TNFSF8 | Inverse variance weighted | 3 | -0.003 | 0.025 | 0.904 |  |  |  |
| Oral Cancer | TNFSF8 | Simple mode | 3 | -0.008 | 0.033 | 0.840 |  |  |  |
| Oral Cancer | TNFSF8 | Weighted mode | 3 | -0.008 | 0.034 | 0.845 |  |  |  |
|  |  |  |  |  |  |  |  |  |  |
| Oral Cancer | HSD17B14 | MR Egger | 3 | -0.519 | 0.436 | 0.445 | MR Egger | 0.843 | 0.448 |
| Oral Cancer | HSD17B14 | Weighted median | 3 | 0.002 | 0.030 | 0.953 | Inverse variance weighted | 0.490 |  |
| Oral Cancer | HSD17B14 | Inverse variance weighted | 3 | -0.006 | 0.024 | 0.801 |  |  |  |
| Oral Cancer | HSD17B14 | Simple mode | 3 | 0.014 | 0.037 | 0.743 |  |  |  |
| Oral Cancer | HSD17B14 | Weighted mode | 3 | 0.012 | 0.036 | 0.764 |  |  |  |
|  |  |  |  |  |  |  |  |  |  |
| Oral Cancer | XXYLT1 | MR Egger | 3 | -0.010 | 0.459 | 0.986 | MR Egger | 0.797 | 0.990 |
| Oral Cancer | XXYLT1 | Weighted median | 3 | -0.007 | 0.030 | 0.822 | Inverse variance weighted | 0.967 |  |
| Oral Cancer | XXYLT1 | Inverse variance weighted | 3 | -0.003 | 0.025 | 0.904 |  |  |  |
| Oral Cancer | XXYLT1 | Simple mode | 3 | -0.008 | 0.034 | 0.842 |  |  |  |
| Oral Cancer | XXYLT1 | Weighted mode | 3 | -0.008 | 0.033 | 0.840 |  |  |  |
|  |  |  |  |  |  |  |  |  |  |

MR,Mendelian Randomization;OC,Oral cancer;Beta,beta regression coefficient;Se,standard error;Heterogeneity, Heterogeneity test;Pleiotropy, Pleiotropy test;Pval,P-value.

Appendix Table 5. SMR and HEIDI results of 8 plasma proteins and OC

| deCODE | | | | | | | | UKB-PPP | | | | | |
| --- | --- | --- | --- | --- | --- | --- | --- | --- | --- | --- | --- | --- | --- |
| TOP SNP | Protein | Gene names | Beta_SMR | Se_SMR | Pval_SMR | HEIDI -pval | nsnp_HEIDI | TOP SNP | Beta_SMR | Se_SMR | Pval_SMR | HEIDI -pval | nsnp_HEIDI |
| rs1006026 | CD30 Ligand | TNFSF8 | 0.71 | 0.22 | 1.26E-03 | 0.90 | 20 | rs10081728 | 0.41 | 0.13 | 1.43E-03 | 0.68 | 20 |
| rs2633298 | uPA | PLAU | -0.33 | 0.12 | 4.23E-03 | 0.98 | 20 | rs55744193 | -0.13 | 0.37 | 0.72 | NA | NA |
| rs6458198 | TREML2 | TREML2 | -0.17 | 0.11 | 0.12 | 0.83 | 20 | rs11759347 | -0.44 | 0.19 | 0.02 | 0.72 | 20 |
| rs148834251 | MSP | MST1 | 0.21 | 0.08 | 0.01 | NA | NA | rs11130213 | 0.05 | 0.03 | 0.09 | 0.49 | 20 |
| rs10146989 | NID2 | NID2 | 0.21 | 0.11 | 0.04 | 0.87 | 20 | rs2516600 | 0.09 | 0.05 | 0.07 | 0.49 | 20 |
| rs62184386 | HIBCH | HIBCH | -0.31 | 0.14 | 0.02 | 0.47 | 20 | NA | NA | NA | NA | NA | NA |
| Additional findings |  |  |  |  |  |  |  |  |  |  |  |  |  |
| rs12695049 | XXLT1 | XXYLT1 | 0.49 | 0.16 | 1.90E-03 | 1.00 | 20 | NA | NA | NA | NA | NA | NA |
| rs35299026 | DHB14 | HSD17B14 | 1.44 | 0.49 | 3.53E-03 | 0.85 | 20 | rs35299026 | 0.15 | 0.05 | 2.04E-03 | 0.93 | 20 |

SMR,Summary-data-based MR;MR,Mendelian Randomization;OC,Oral cancer;SNP,Single nucleotide polymorphism;Beta,beta regression coefficient;Se,standard error;Pval,P-value;HEIDI, Heterogeneity in dependent instruments test;nSNP, Number of SNPs.

Appendix Table 6. Colocalization results of 8 significant associated proteins with OC

| Gene | protein names | protein full name | PP.H0 | PP.H1 | PP.H2 | PP.H3 | PP.H4 | PP.H0 | PP.H1 | PP.H2 | PP.H3 | PP.H4 |
| --- | --- | --- | --- | --- | --- | --- | --- | --- | --- | --- | --- | --- |
| deCODE | | | | | | | | UKB-PPP | | | | |
| PLAU | uPA | Urokinase-type plasminogen activator | 0.00E+00 | 3.89E-01 | 0.00E+00 | 5.78E-01 | 3.28E-02 | 4.07E-72 | 4.07E-72 | 4.07E-72 | 5.52E-01 | 3.52E-02 |
| TNFSF8 | CD30 Ligand | Tumor necrosis factor ligand superfamily member 8 | 2.79E-57 | 2.79E-57 | 3.45E-57 | 1.88E-01 | 6.59E-01 | 2.09E-01 | 2.09E-01 | 2.45E-208 | 2.56E-01 | 5.35E-01 |
| NID2 | NID2 | Nidogen 2 | 0.00E+00 | 4.19E-01 | 0.00E+00 | 5.49E-01 | 3.25E-02 | 0.00E+00 | 4.19E-01 | 0.00E+00 | 2.91E-02 | 2.91E-02 |
| TREML2 | TREM2 | Triggering receptor expressed on myeloid cells like 2 | 0.00E+00 | 5.42E-01 | 0.00E+00 | 4.16E-01 | 4.17E-02 | 4.93E-01 | 4.93E-01 | 3.61E-01 | 3.61E-01 | 1.46E-01 |
| MST1 | MSP | Macrophage stimulating 1 | 0.00E+00 | 6.46E-01 | 0.00E+00 | 3.10E-01 | 4.42E-02 | 0.00E+00 | 7.00E-01 | 0.00E+00 | 2.53E-01 | 4.62E-02 |
| HIBCH | HIBCH | 3-hydroxyisobutyryl-CoA hydrolase | 0.00E+00 | 5.63E-01 | 0.00E+00 | 3.84E-01 | 5.28E-02 | NA | NA | NA | NA | NA |
| Additional findings |  |  |  |  |  |  |  |  |  |  |  |  |
| HSD17B14 | DHB14 | 17-beta-hydroxysteroid dehydrogenase 14 | 6.56E-14 | 2.59E-01 | 4.92E-14 | 1.94E-01 | 5.47E-01 | 0.00E+00 | 2.53E-01 | 0.00E+00 | 1.71E-01 | 5.76E-01 |
| XXYLT1 | XXLT1 | Xyloside xylosyltransferase 1 | 2.58E-120 | 2.26E-01 | 2.27E-120 | 1.99E-01 | 5.75E-01 | NA | NA | NA | NA | NA |

OC, Oral cancer;H0 ：No association with either trait;H1：Association with trait 1, not with trait 2;H2：Association with trait 2, not with trait 1;H3: Association with trait 1 and trait 2, two independent SNPs;H4: Association with trait 1 and trait 2, one shared SNP;

Appendix Table 7. Results of SMR,Heidi tests and MR for eQTLs and OC

| ProbeID | Top SNP | Gene names | Protein names | Beta_SMR | Se_SMR | Pval_SMR | Pval_HEIDI | nsnp_HEIDI |  |  |  |
| --- | --- | --- | --- | --- | --- | --- | --- | --- | --- | --- | --- |
| ENSG00000173950 | rs12695049 | XXYLT1 | XXLT1 | 0.49 | 0.16 | 0.00 | 0.43 | 17 |  |  |  |
| ENSG00000106952 | rs1006026 | TNFSF8 | CD30 Ligand | 0.72 | 0.22 | 0.00 | 0.54 | 16 |  |  |  |
| ENSG00000087076 | rs4802517 | HSD17B14 | DHB14 | 0.63 | 0.65 | 0.33 | 0.59 | 7 |  |  |  |
| ENSG00000122861 | rs2688623 | PLAU | uPA | -0.44 | 0.17 | 0.01 | 0.27 | 12 |  |  |  |
| ENSG00000112195 | rs4714431 | TREML2 | TREML2 | -0.18 | 0.07 | 0.01 | 0.57 | 20 |  |  |  |
| ENSG00000087303 | rs2516600 | NID2 | NID2 | 0.11 | 0.06 | 0.07 | 0.02 | 19 |  |  |  |
| ENSG00000173531 | rs9858213 | MST1 | MSP | 0.05 | 0.03 | 0.08 | 0.72 | 20 |  |  |  |
| ENSG00000198130 | rs291466 | HIBCH | HIBCH | -0.18 | 0.09 | 0.06 | 0.93 | 20 |  |  |  |
|  |  |  |  |  |  |  |  |  |  |  |  |
| Gene names | Protein names | Method | nSNP | Beta | Se | Pval | OR95%CI | Egger_intercept | Pleiotropy pval | Q_pval | MR Egger Q_pval |
| XXYLT1 | XXLT1 | Inverse variance weighted | 38 | 0.20 | 0.10 | 0.05 | 1.22(1.00-1.48) | 0.01 | 0.42 | 0.27 | 0.26 |
| TNFSF8 | CD30 Ligand | Inverse variance weighted | 35 | 0.30 | 0.11 | 0.01 | 1.35(1.09-1.67) | -0.01 | 0.72 | 0.27 | 0.24 |
| HSD17B14 | DHB14 | Inverse variance weighted | 23 | 0.02 | 0.12 | 0.84 | 1.02(0.81-1.29) | -0.04 | 0.10 | 0.07 | 0.13 |
| PLAU | uPA | Inverse variance weighted | 12 | -0.44 | 0.16 | 0.01 | 0.65(0.47-0.88) | -0.06 | 0.21 | 0.56 | 0.64 |
| TREML2 | TREML2 | Inverse variance weighted | 34 | -0.32 | 0.10 | 0.00 | 0.73(0.60-0.89) | 0.00 | 0.81 | 0.41 | 0.36 |
| NID2 | NID2 | Inverse variance weighted | 2 | 0.03 | 0.68 | 0.97 | 1.03(0.27-3.93) | NA | NA | 0.06 | NA |
| MST1 | MSP | Inverse variance weighted | 10 | -0.07 | 0.10 | 0.50 | 0.94(0.77-1.14) | 0.07 | 0.07 | 0.68 | 0.98 |
| HIBCH | HIBCH | Inverse variance weighted | 56 | -0.16 | 0.06 | 0.01 | 0.86(0.76-0.96) | -0.02 | 0.10 | 0.93 | 0.96 |

SMR,Summary-data-based MR;MR, Mendelian Randomization;HEIDI, Heterogeneity in dependent instruments test;eQTL,Expression quantitative trait loci;Heterogeneity, Heterogeneity Test; SNP,Single nucleotide polymorphism;Beta,beta regression coefficient;Se,standard error;(95%CI),95%Confidence interval;OR,Odds ratio;Pval,P-value; FDR,False Discovery Rate;Pleiotropy, Pleiotropy Test; Q_pval, horizontal pleiotropy P-value for Q-test.

Appendix Table 8. The MR results between variable risk factors and oral cancer

| Exposure Trait | IVW（Wald ratio) | | | Weighted median | | | MR Egger | | | Simple mode | | | Weighted mode | | |
| --- | --- | --- | --- | --- | --- | --- | --- | --- | --- | --- | --- | --- | --- | --- | --- |
| Anthropometric traits | SNPs | OR(95%CI) | Pval | SNPs | OR(95%CI) | Pval | SNPs | OR(95%CI) | Pval | SNPs | OR(95%CI) | Pval | SNPs | OR(95%CI) | Pval |
| BMI | 72 | 1.000(0.998-1.002) | 0.783 | 72 | 1.000(0.998-1.002) | 1.000 | 72 | 0.997(0.993-1.002) | 0.247 | 72 | 1.006(0.815-1.241) | 0.958 | 72 | 1.006(0.979-1.032) | 0.677 |
| HIP | 5 | 1.002(0.997-1.006) | 0.441 | 5 | 1.002(0.997-1.006) | 0.573 | 5 | 1.007(0.990-1.025) | 0.475 | 5 | 1.003(0.996-1.010) | 0.486 | 5 | 1.003(0.996-1.009) | 0.459 |
| HIP(adjBMI) | 11 | 1.002(0.999-1.006) | 0.252 | 11 | 1.002(0.998-1.006) | 0.284 | 11 | 1.002(0.991-1.013) | 0.733 | 11 | 1.153(0.810-1.640) | 0.448 | 11 | 1.003(0.988-1.018) | 0.742 |
| WC(adjBMI) | 18 | 1.004(0.998-1.009) | 0.206 | 18 | 1.004(0.998-1.009) | 0.187 | 18 | 1.000(0.975-1.026) | 0.986 | 18 | 1.361(0.575-3.223) | 0.493 | 18 | 1.013(0.991-1.035) | 0.256 |
| WHR | 17 | 0.997(0.992-1.002) | 0.239 | 17 | 0.996(0.990-1.002) | 0.237 | 17 | 0.995(0.954-1.038) | 0.822 | 17 | 0.985(0.538-1.802) | 0.961 | 17 | 0.999(0.980-1.019) | 0.949 |
| WHR(adjBMI) | 34 | 0.998(0.995-1.001) | 0.315 | 34 | 0.999(0.995-1.003) | 0.598 | 34 | 1.011(0.984-1.040) | 0.428 | 34 | 1.000(0.710-1.408) | 0.999 | 34 | 1.000(0.978-1.022) | 0.989 |
| Basal metabolic rate | 467 | 0.999(0.998-1.001) | 0.384 | 467 | 1.000(0.998-1.002) | 1.000 | 467 | 1.001(0.997-1.004) | 0.682 | 467 | 1.000(0.847-1.182) | 0.996 | 467 | 1.000(0.933-1.073) | 0.990 |
| Arm fat mass (left) | 359 | 1.000(0.999-1.002) | 0.722 | 359 | 1.000(0.998-1.002) | 1.000 | 359 | 1.000(0.998-1.002) | 0.751 | 359 | 1.025(0.900-1.168) | 0.710 | 359 | 0.977(0.931-1.025) | 0.342 |
| Body fat percentage | 332 | 1.000(0.998-1.002) | 0.849 | 332 | 1.000(0.997-1.003) | 1.000 | 332 | 0.998(0.992-1.004) | 0.555 | 332 | 1.058(0.854-1.031) | 0.608 | 332 | 0.955(0.868-1.050) | 0.340 |
| Waist circumference | 320 | 1.000(0.998-1.002) | 0.858 | 320 | 1.000(0.997-1.003) | 1.000 | 320 | 1.001(0.995-1.006) | 0.795 | 320 | 0.945(0.805-1.019) | 0.486 | 320 | 1.021(0.972-1.074) | 0.407 |
| Whole body fat mass | 371 | 1.000(0.999-1.001) | 0.879 | 371 | 1.000(0.998-1.002) | 1.000 | 371 | 1.000(0.997-1.004) | 0.821 | 371 | 1.048(0.915-1.200) | 0.498 | 371 | 0.998(0.951-1.048) | 0.939 |
| Trunk fat mass | 359 | 1.001(0.998-1.002) | 0.391 | 359 | 1.000(0.998-1.002) | 1.000 | 359 | 1.001(0.997-1.004) | 0.687 | 359 | 1.049(0.908-1.212) | 0.515 | 359 | 1.000(0.957-1.046) | 0.978 |
| Leg fat mass (left) | 358 | 1.000(0.998-1.002) | 0.911 | 358 | 1.000(0.997-1.003) | 1.000 | 358 | 1.001(0.997-1.005) | 0.645 | 358 | 1.027(0.867-1.216) | 0.758 | 358 | 0.984(0.936-1.035) | 0.540 |
| diastolic blood pressure | 397 | 1.000(1.000-1.000) | 0.976 | 397 | 1.000(1.000-1.000) | 1.000 | 397 | 1.000(1.000-1.000) | 0.419 | 397 | 0.999(0.986-1.012) | 0.869 | 397 | 0.999(0.994-1.004) | 0.637 |
| Overall health rating | 96 | 1.000(0.995-1.006) | 0.861 | 96 | 1.000(0.992-1.008) | 1.000 | 96 | 0.992(0.971-1.013) | 0.445 | 96 | 0.930(0.494-1.751) | 0.824 | 96 | 1.006(0.940-1.078) | 0.854 |
| Lifestyle factors |  |  |  |  |  |  |  |  |  |  |  |  |  |  |  |
| Coffee consumption | 26 | 0.997(0.987-1.006) | 0.515 | 26 | 0.998(0.987-1.009) | 0.757 | 26 | 0.969(0.890-1.056) | 0.481 | 26 | 0.488(0.165-1.447) | 0.208 | 26 | 0.993(0.956-1.033) | 0.740 |
| Bitter alcoholic beverages | 25 | 1.000(0.988-1.012) | 0.968 | 25 | 0.999(0.986-1.012) | 0.842 | 25 | 1.028(0.981-1.077) | 0.260 | 25 | 6.891(1.722-27.580) | 0.012 | 25 | 0.996(0.930-1.068) | 0.917 |
| Tea consumption | 9 | 1.266(0.198-8.079) | 0.803 | 9 | 1.527(0.171-13.642) | 0.705 | 9 | 40.263(0.151-10768.852) | 0.236 | 9 | 1.095(0.036-32.868) | 0.960 | 9 | 1.817(0.087-38.071) | 0.710 |
| Age at first sexual intercourse | 153 | 1.001(0.999-1.004) | 0.233 | 153 | 1.000(0.997-1.003) | 1.000 | 153 | 1.006(0.996-1.017) | 0.265 | 153 | 1.004(0.815-1.236) | 0.973 | 153 | 1.004(0.952-1.058) | 0.896 |
| Lifetime number of sexual partners | 54 | 0.998(0.993-1.003) | 0.493 | 54 | 1.000(0.993-1.007) | 1.000 | 54 | 0.966(0.945-0.988) | 0.003 | 54 | 1.030(0.662-1.603) | 0.896 | 54 | 0.999(0.964-1.036) | 0.975 |
| Alcohol intake versus 10 years previously | 7 | 1.014(1.002-1.026) | 0.025 | 7 | 1.008(0.996-1.021) | 0.185 | 7 | 1.143(0.922-1.416) | 0.277 | 7 | 1.140(0.671-1.936) | 0.644 | 7 | 1.023(0.996-1.050） | 0.147 |
| Smoking Cessation | 10 | 1.931(0.853-4.371) | 0.114 | 10 | 3.147(1.095-9.041) | 0.033 | 10 | 1.063(0.077-14.725） | 0.965 | 10 | 3.668(0.755-17.820) | 0.142 | 10 | 3.554(0.901-14.018) | 0.104 |
| Age of Initiation of Regular Smoking | 6 | 1.005(0.995-1.016) | 0.317 | 6 | 1.005(0.995-1.016) | 0.338 | 6 | 0.226(0.000-193.747） | 0.688 | 6 | 0.671(0.153-2.932) | 0.618 | 6 | 1.004(0.982-1.027) | 0.736 |
| Smoking Initiation | 178 | 1.000(0.998-1.002) | 0.996 | 178 | 1.000(0.998-1.002) | 1.000 | 178 | 0.997(0.990-1.005) | 0.502 | 178 | 1.036(0.899-1.194) | 0.628 | 178 | 1.005(0.974-1.037) | 0.736 |
| Cigarettes per Day | 20 | 1.445(1.088-1.945) | 0.011 | 20 | 1.490(0.969-2.289) | 0.069 | 20 | 1.269(0.709-2.274） | 0.433 | 20 | 1.595(0.831-3.062) | 0.177 | 20 | 1.498(0.930-2.411) | 0.113 |
| Smoking status: Never | 63 | 1.002(0.995-1.010) | 0.523 | 63 | 1.000(0.991-1.009) | 1.000 | 63 | 1.016(0.975-1.059) | 0.442 | 63 | 0.728(0.387-1.369) | 0.328 | 63 | 0.971(0.903-1.044) | 0.434 |
| Current tobacco smoking | 15 | 1.021(1.000-1.042) | 0.045 | 15 | 1.000(0.998-1.002) | 0.067 | 15 | 1.000(0.998-1.002) | 0.214 | 15 | 1.000(0.998-1.002) | 0.752 | 15 | 1.000(0.998-1.002) | 0.202 |
| Drinks per Week | 30 | 0.995(0.989-1.002) | 0.138 | 30 | 0.995(0.988-1.001) | 0.122 | 30 | 0.999(0.975-1.024) | 0.967 | 30 | 1.153(0.604-2.203) | 0.669 | 30 | 0.992(0.962-1.023) | 0.621 |
| Daytime nap | 85 | 0.996(0.991-1.000) | 0.031 | 85 | 1.000(0.994-1.006) | 1.000 | 85 | 1.002(0.991-1.014) | 0.717 | 85 | 0.839(0.559-1.259) | 0.400 | 85 | 0.984(0.929-1.043) | 0.594 |
| insomnia | 32 | 0.999(0.994-1.005) | 0.853 | 32 | 1.001(0.993-1.008) | 0.870 | 32 | 1.005(0.994-1.016) | 0.408 | 32 | 1.515(0.612-3.752) | 0.376 | 32 | 0.993(0.953-1.034) | 0.722 |
| Time spent doing light physical activity | 3 | 1.010(0.996-1.025) | 0.156 | 3 | 1.008(0.991-1.025) | 0.359 | 3 | 1.028(0.987-1.071) | 0.410 | 3 | 1.006(0.984-1.028) | 0.647 | 3 | 1.007(0.988)-1.026) | 0.557 |
| Time spent driving | 6 | 1.004(0.993-1.015) | 0.489 | 6 | 1.004(0.992-1.016) | 0.536 | 6 | 0.843(0.526-1.350) | 0.516 | 6 | 0.729(0.422-1.257) | 0.307 | 6 | 1.003(0.983-1.024) | 0.792 |
| Time spent using computer | 75 | 1.002(0.998-1.007) | 0.291 | 75 | 1.004(0.998-1.010) | 0.146 | 75 | 1.007(0.983-1.032) | 0.579 | 75 | 0.911(0.555-1.496) | 0.715 | 75 | 0.990(0.946-1.036) | 0.657 |
| Time spent watching television (TV) | 100 | 1.003(0.999-1.008) | 0.156 | 100 | 1.000(0.995-1.006) | 0.899 | 100 | 0.997(0.972-1.024) | 0.845 | 100 | 1.517(0.972-2.367) | 0.070 | 100 | 0.992(0.944-1.042) | 0.753 |
| Education and family factors |  |  |  |  |  |  |  |  |  |  |  |  |  |  |  |
| Years of schooling | 20 | 1.000(0.995-1.005) | 0.989 | 20 | 1.000(0.994-1.006) | 1.000 | 20 | 0.999(0.940-1.063) | 0.982 | 20 | 0.885(0.419-1.871) | 0.753 | 20 | 1.001(0.985-1.016) | 0.940 |
| Subjective well being | 1 | 2.425(0.087-67.248) | 0.601 |  |  |  |  |  |  |  |  |  |  |  |  |
| Average total household income before tax | 44 | 0.996(0.992-1.001) | 0.118 | 44 | 0.998(0.993-1.003) | 0.483 | 44 | 0.961(0.924-0.999) | 0.051 | 44 | 1.206(0.753-1.929) | 0.440 | 44 | 0.984(0.952-1.017) | 0.335 |
| College or University degree | 216 | 1.003(0.999-1.008) | 0.149 | 216 | 1.000(0.994-1.006) | 1.000 | 216 | 1.002(0.984-1.020) | 0.851 | 216 | 0.981(0.718-1.341) | 0.905 | 216 | 0.981(0.901-1.068) | 0.661 |
| Cardiovascular disease |  |  |  |  |  |  |  |  |  |  |  |  |  |  |  |
| hypertension | 53 | 0.994(0.979-1.009) | 0.423 | 53 | 1.000(0.985-1.015) | 1.000 | 53 | 0.982(0.929-1.039) | 0.533 | 53 | 1.036(0.165-6.507) | 0.970 | 53 | 1.036(0.922-1.164) | 0.557 |
| Coronary heart disease | 13 | 1.001(0.999-1002) | 0.431 | 13 | 1.001(0.999-1002) | 0.361 | 13 | 1.008(0.999-1.017) | 0.106 | 13 | 1.002(0.863-1.163) | 0.983 | 13 | 1.002(0.998-1.006) | 0.449 |
| Coronary artery disease | 132 | 1.000(0.999-1.001) | 0.447 | 132 | 1.000(0.999-1.001) | 1.000 | 132 | 1.002(1.000-1.005) | 0.049 | 132 | 0.950(0.865-1.044) | 0.287 | 132 | 1.002(0.987-1.018) | 0.763 |
| Atrial fibrillation | 99 | 1.001(1.000-1.001) | 0.186 | 99 | 1.001(1.000-1.002) | 0.182 | 99 | 1.002(1.000-1.004) | 0.100 | 99 | 0.989(0.919-1.065) | 0.770 | 99 | 1.004(0.994-1.013) | 0.451 |
| Heart failure | 9 | 1.000(0.997-1.003) | 0.997 | 9 | 1.000(0.997-1.003) | 1.000 | 9 | 1.332(0.343-5.089) | 0.697 | 9 | 0.921(0.556-1.527) | 0.758 | 9 | 0.995(0.985-1.004) | 0.301 |
| Stroke | 16 | 0.999(0.997-1.001) | 0.377 | 16 | 0.999(0.997-1.001) | 0.269 | 16 | 1.005(0.987-1.024) | 0.573 | 16 | 1.055(0.821-1.356) | 0.681 | 16 | 0.998(0.989-1.007) | 0.650 |
| Immune system |  |  | (p.adj) |  |  |  |  |  |  |  |  |  |  |  |  |
| Effector Memory CD4+ T cell %T cell | 1 | 0.454(0.325-0.634) | 0.003 |  |  |  |  |  |  |  |  |  |  |  |  |
| CD45RA+ CD8+ T cell Absolute Count | 1 | 2.866(1.835-4.476) | 0.003 |  |  |  |  |  |  |  |  |  |  |  |  |
| CD4 on HLA DR+ CD4+ T cell | 1 | 2.169(1.478-3.185) | 0.028 |  |  |  |  |  |  |  |  |  |  |  |  |
| CD8 on CD28+ CD45RA- CD8+ T cell | 1 | 2.008(1.418-2.843) | 0.028 |  |  |  |  |  |  |  |  |  |  |  |  |
| CD28 on CD45RA- CD4 not regulatory T cell | 2 | 1.002(1.001-1.003) | 0.028 |  |  |  |  |  |  |  |  |  |  |  |  |
| CD8+ T cell %leukocyte | 1 | 1.921(1.374-2.686) | 0.038 |  |  |  |  |  |  |  |  |  |  |  |  |
| T2D and Related Trait |  |  |  |  |  |  |  |  |  |  |  |  |  |  |  |
| Fasting glucose | 59 | 1.001(0.999-1.003) | 0.456 | 59 | 1.001(0.998-1.004) | 0.519 | 59 | 1.001(0.997-1.005) | 0.712 | 59 | 0.745(0.481-1.156) | 0.194 | 59 | 0.997(0.965-1.03) | 0.855 |
| Fasting insulin | 34 | 0.997(0.991-1.004) | 0.444 | 34 | 0.998(0.991-1.004) | 0.466 | 34 | 0.971(0.890-1.059) | 0.507 | 34 | 1.125(0.274-4.618) | 0.871 | 34 | 0.988(0.950-1.028) | 0.552 |
| HbA1c levels | 326 | 1.001(1.000-1.002) | 0.070 | 326 | 1.001(1.000-1.003) | 0.091 | 326 | 1.002(0.999-1.004) | 0.163 | 326 | 0.968(0.850-1.099) | 0.630 | 326 | 1.006(0.966-1.046) | 0.775 |
| Type 2 diabetes | 201 | 1.000(0.999-1.001) | 0.598 | 201 | 1.000(0.999-1.001) | 1.000 | 201 | 1.000(0.998-1.002) | 0.944 | 201 | 0.898(0.819-0.985) | 0.023 | 201 | 0.993(0.978-1.008) | 0.333 |
| Serum metabolite |  |  |  |  |  |  |  |  |  |  |  |  |  |  |  |
| Alanine | 29 | 1.002(0.998-1.006) | 0.260 | 29 | 1.002(0.999-1.005) | 0.196 | 29 | 1.000(0.989-1.011) | 0.987 | 29 | 1.032(0.470-2.268) | 0.938 | 29 | 1.009(0.991-1.028) | 0.345 |
| Glutamine | 35 | 0.999(0.996-1.001) | 0.343 | 35 | 0.999(0.996-1.001) | 0.330 | 35 | 1.136(0.873-1.477) | 0.349 | 35 | 1.052(0.625-1.769) | 0.851 | 35 | 0.997(0.979-1.015) | 0.719 |
| Glycine | 36 | 0.998(0.991-1.006) | 0.655 | 36 | 0.998(0.992-1.004) | 0.522 | 36 | 1.131(0.996-1.285) | 0.066 | 36 | 0.907(0.596-1.380) | 0.650 | 36 | 1.010(0.986-1.035) | 0.408 |
| Histidine | 11 | 0.524(0.322-0.852) | 0.009 | 11 | 0.474(0.251-0.897) | 0.022 | 11 | 0.343(0.104-1.129) | 0.112 | 11 | 0.929(0.283-3.047) | 0.905 | 11 | 0.393(0.180-0.858) | 0.041 |
| Isoleucine | 10 | 1.001(0.998-1.005) | 0.486 | 10 | 1.001(0.997-1.005) | 0.584 | 10 | 1.005(0.991-1.019) | 0.516 | 10 | 1.016(0.806-1.281) | 0.893 | 10 | 1.006(0.997-1.015) | 0.214 |
| Leucine | 14 | 1.001(0.998-1.005) | 0.522 | 14 | 1.001(0.998-1.004) | 0.600 | 14 | 1.005(0.990-1.020) | 0.532 | 14 | 0.777(0.534-1.132) | 0.211 | 14 | 1.000(0.985-1.014) | 0.974 |
| 25-Hydroxyvitamin D levels | 99 | 1.000(0.998-1.003) | 0.694 | 99 | 1.000(0.997-1.003) | 1.000 | 99 | 1.003(0.998-1.009) | 0.245 | 99 | 1.087(0.897-1.317) | 0.397 | 99 | 0.994(0.961-1.028) | 0.732 |
| Tyrosine | 28 | 1.076(0.749-1.548) | 0.691 | 28 | 1.331(0.873-2.030) | 0.184 | 28 | 1.379(0.781-2.437) | 0.278 | 28 | 0.930(0.314-2.757) | 0.897 | 28 | 1.355(0.908-2.023) | 0.149 |
| Sex hormone |  |  |  |  |  |  |  |  |  |  |  |  |  |  |  |
| Testosterone levels | 83 | 0.996(0.992-1.000) | 0.045 | 83 | 0.995(0.991-1.000) | 0.065 | 83 | 0.995(0.988-1.002) | 0.167 | 83 | 1.121(0.522-2.409) | 0.770 | 83 | 0.977(0.926-1.029) | 0.381 |
| Oestradiol levels | 1 | 0.999(0.994-1.003) | 0.491 |  |  |  |  |  |  |  |  |  |  |  |  |
| eQTLs |  |  |  |  |  |  |  |  |  |  |  |  |  |  |  |
| TNFSF8 levels | 23 | 1.29(1.20-1.39) | 8.53E-13 | 23 | 1.26(1.14-1.40) | 9.48E-06 | 23 | 1.23(1.07-1.40) | 0.01 | 23 | 1.28(1.05-1.57) | 0.02 | 23 | 1.21(1.05-1.38) | 0.01 |
| XXYLT1 levels | 9 | 1.19(1.08-1.31) | 4.63E-04 | 9 | 1.14(0.99-1.30) | 0.07 | 9 | 0.93(0.73-1.20) | 0.61 | 9 | 1.11(0.90-1.36) | 0.36 | 9 | 1.09(0.90-1.33) | 0.41 |
| HSD17B14 levels | 23 | 1.02(0.81-1.29) | 0.84 | 23 | 1.18(0.89-1.55) |  | 23 | 1.40(0.92-2.13) | 0.13 | 23 | 1.14(0.65-2.02) | 0.65 | 23 | 1.21(0.93-1.57) | 0.16 |

MR,Mendelian randomization;BMI,body mass index;HIP,Hip circumference;WC,Waist circumference;WHR,waist-to-hip ratio;SNP,Single nucleotide polymorphism;IVW, Inverse variance weighted;OR,Odds ratio ;(95%CI),95%Confidence interval;

Appendix Table 9. Results of Mediation Analysis from Gene Expression to OC Risk via Protein Levels

| Exposure | Mediator | Total Effect β (95% CI) | Direct Effect β1 (95% CI) | Direct Effect β2 (95% CI) | Mediated effect（β1*β2） | Mediated effect ratios（β1*β2/β）*100% | Total Pval | Direct Pval | Direct Pval 2 |
| --- | --- | --- | --- | --- | --- | --- | --- | --- | --- |
| XXYLT1 eQTL | XXYLT1 pQTL | 0.20(1.00-1.48) | 0.17(1.08-1.31) | 0.29(1.15-1.55) | 0.05 | 24.65% | 0.05 | 4.63E-04 | 1.14E-04 |
| TNFSF8 eQTL | TNFSF8 pQTL | 0.30(1.09-1.67) | 0.26(1.20-1.39) | 0.84(1.72-3.11) | 0.22 | 72.80% | 5.28E-03 | 8.53E-13 | 2.49E-08 |

MR, Mendelian randomization;(95%CI), 95%Confidence interval;OC, Oral caner;β, Variable - outcome effect value;β1, Modifiable factor-protein effect value;β2, protein-outcome effect value;eQTL,expression quantitative trait loci;pQTL,protein quantitative trait loci;

Appendix Table 10. Results of Mendelian randomization analysis of other immune cells and oral cancer(Not positive)

| Exposure | Method | nsnp | Beta | Se | Pval | Pval-FDR | OR(95%CI) |
| --- | --- | --- | --- | --- | --- | --- | --- |
| Naive CD4+ T cell Absolute Count | Wald ratio | 1 | 0.863 | 0.233 | 0.000 | 0.052 | 2.370(1.499-3.745) |
| CD45 on CD4+ T cell | Wald ratio | 1 | -0.950 | 0.287 | 0.001 | 0.195 | 0.387(0.220-0.679) |
| CD64 on CD14+ CD16+ monocyte | Inverse variance weighted | 7 | 0.000 | 0.000 | 0.002 | 0.321 | 1.000(1.000-1.001) |
| CD28 on secreting CD4 regulatory T cell | Inverse variance weighted | 3 | 0.002 | 0.001 | 0.003 | 0.325 | 1.002(1.001-1.002) |
| CD45RA on Terminally Differentiated CD8+ T cell | Wald ratio | 1 | 0.001 | 0.000 | 0.003 | 0.325 | 1.001(1.000-1.002) |
| CD64 on monocyte | Inverse variance weighted | 7 | 0.000 | 0.000 | 0.003 | 0.325 | 1.000(1.000-1.000) |
| CD64 on CD14+ CD16- monocyte | Inverse variance weighted | 7 | 0.000 | 0.000 | 0.003 | 0.325 | 1.000(1.000-1.000) |
| CD8 on Natural Killer T | Wald ratio | 1 | 1.070 | 0.350 | 0.002 | 0.325 | 2.916(1.468-5.793) |
| CD64 on CD14- CD16- | Inverse variance weighted | 3 | 0.001 | 0.000 | 0.003 | 0.325 | 1.001(1.000-1.002) |
| CD8 on Terminally Differentiated CD8+ T cell | Wald ratio | 1 | -0.534 | 0.201 | 0.008 | 0.727 | 0.586(0.395-0.869) |
| CCR7 on naive CD4+ T cell | Inverse variance weighted | 4 | 0.042 | 0.063 | 0.512 | 1.000 | 1.043(0.921-1.181) |
| CD25++ CD45RA- CD4 not regulatory T cell %CD4+ T cell | Inverse variance weighted | 3 | 0.064 | 0.065 | 0.324 | 1.000 | 1.066(0.939-1.210) |
| HLA DR+ CD4+ T cell Absolute Count | Inverse variance weighted | 2 | 0.269 | 0.253 | 0.287 | 1.000 | 1.309(0.798-2.147) |
| CX3CR1 on monocyte | Inverse variance weighted | 2 | 0.000 | 0.000 | 0.317 | 1.000 | 1.000(0.999-1.000) |
| CD20 on IgD- CD24- B cell | Inverse variance weighted | 2 | 0.057 | 0.427 | 0.893 | 1.000 | 1.059(0.459-2.447) |
| CD34 on Hematopoietic Stem Cell | Inverse variance weighted | 4 | -0.113 | 0.080 | 0.154 | 1.000 | 0.893(0.764-1.044) |
| CD11c+ CD62L- monocyte Absolute Count | Wald ratio | 1 | 0.000 | 0.001 | 1.000 | 1.000 | 1.000(0.999-1.001) |
| CD86 on myeloid Dendritic Cell | Wald ratio | 1 | 0.000 | 0.000 | 1.000 | 1.000 | 1.000(1.000-1.000) |
| CD127 on granulocyte | Inverse variance weighted | 7 | 0.060 | 0.087 | 0.489 | 1.000 | 1.062(0.896-1.259) |
| CD20 on IgD- CD38dim B cell | Inverse variance weighted | 6 | 0.078 | 0.077 | 0.312 | 1.000 | 1.081(0.929-1.259) |
| CD62L- CD86+ myeloid Dendritic Cell Absolute Count | Inverse variance weighted | 5 | 0.000 | 0.000 | 0.908 | 1.000 | 1.000(1.000-1.000) |
| HLA DR on CD33dim HLA DR+ CD11b+ | Inverse variance weighted | 3 | -0.014 | 0.175 | 0.936 | 1.000 | 0.986(0.700-1.389) |
| CD28- CD8dim T cell %CD8dim T cell | Wald ratio | 1 | 0.555 | 0.376 | 0.140 | 1.000 | 1.742(0.833-3.642) |
| CD19 on CD20- CD38- B cell | Wald ratio | 1 | -0.066 | 0.253 | 0.794 | 1.000 | 0.936(0.570-1.537) |
| CD11c on granulocyte | Inverse variance weighted | 3 | 0.000 | 0.000 | 0.315 | 1.000 | 1.000(1.000-1.001) |
| CD11c on CD62L+ myeloid Dendritic Cell | Wald ratio | 1 | 0.064 | 0.089 | 0.476 | 1.000 | 1.066(0.894-1.270) |
| CD39+ resting CD4 regulatory T cell %resting CD4 regulatory T cell | Inverse variance weighted | 5 | 0.000 | 0.000 | 0.617 | 1.000 | 1.000(1.000-1.000) |
| HVEM on naive CD4+ T cell | Wald ratio | 1 | 0.169 | 0.199 | 0.396 | 1.000 | 1.184(0.801-1.750) |
| CD24 on transitional B cell | Wald ratio | 1 | -0.061 | 0.151 | 0.687 | 1.000 | 0.941(0.700-1.265) |
| HLA DR on CD14- CD16+ monocyte | Inverse variance weighted | 5 | -0.036 | 0.135 | 0.788 | 1.000 | 0.964(0.741-1.256) |
| Resting CD4 regulatory T cell Absolute Count | Inverse variance weighted | 3 | 0.001 | 0.001 | 0.194 | 1.000 | 1.001(1.000-1.002) |
| CD80 on myeloid Dendritic Cell | Inverse variance weighted | 4 | 0.000 | 0.000 | 0.999 | 1.000 | 1.000(0.999-1.001) |
| IgD- CD38dim B cell %lymphocyte | Inverse variance weighted | 2 | 0.001 | 0.001 | 0.353 | 1.000 | 1.001(0.999-1.002) |
| Terminally Differentiated CD4-CD8- T cell Absolute Count | Wald ratio | 1 | -0.180 | 0.364 | 0.622 | 1.000 | 0.836(0.409-1.706) |
| Terminally Differentiated CD4+ T cell %T cell | Inverse variance weighted | 2 | -0.428 | 0.421 | 0.310 | 1.000 | 0.652(0.285-1.489) |
| CD62L- plasmacytoid Dendritic Cell Absolute Count | Inverse variance weighted | 2 | -0.005 | 0.115 | 0.962 | 1.000 | 0.995(0.793-1.247) |
| BAFF-R on IgD+ CD38- naive B cell | Inverse variance weighted | 7 | 0.036 | 0.056 | 0.517 | 1.000 | 1.037(0.930-1.156) |
| CD20 on unswitched memory B cell | Inverse variance weighted | 3 | 0.085 | 0.107 | 0.429 | 1.000 | 1.089(0.882-1.344) |
| Resting CD4 regulatory T cell %CD4 regulatory T cell | Inverse variance weighted | 8 | 0.000 | 0.000 | 0.642 | 1.000 | 1.000(1.000-1.000) |
| Naive CD8+ T cell %T cell | Inverse variance weighted | 3 | 0.000 | 0.001 | 0.966 | 1.000 | 1.000(0.998-1.002) |
| CD14 on Monocytic Myeloid-Derived Suppressor Cells | Wald ratio | 1 | 0.074 | 0.105 | 0.482 | 1.000 | 1.077(0.876-1.324) |
| CD25 on IgD+ CD38- unswitched memory B cell | Wald ratio | 1 | -0.061 | 0.082 | 0.458 | 1.000 | 0.941(0.801-1.105) |
| CD16+ monocyte %monocyte | Inverse variance weighted | 2 | 0.000 | 0.000 | 0.711 | 1.000 | 1.000(1.000-1.000) |
| CD33 on CD33+ HLA DR+ | Inverse variance weighted | 3 | -0.004 | 0.026 | 0.877 | 1.000 | 0.996(0.946-1.048) |
| CD127 on CD45RA+ CD4+ T cell | Inverse variance weighted | 2 | 0.305 | 0.344 | 0.374 | 1.000 | 1.357(0.692-2.661) |
| CD86 on granulocyte | Inverse variance weighted | 2 | -0.265 | 0.323 | 0.412 | 1.000 | 0.767(0.408-1.444) |
| CD3 on Natural Killer T | Inverse variance weighted | 2 | -0.191 | 0.161 | 0.236 | 1.000 | 0.826(0.602-1.133) |
| CD45 on Immature Myeloid-Derived Suppressor Cells | Inverse variance weighted | 2 | 0.025 | 0.053 | 0.636 | 1.000 | 1.026(0.924-1.139) |
| BAFF-R on IgD+ CD38- unswitched memory B cell | Inverse variance weighted | 6 | 0.016 | 0.039 | 0.690 | 1.000 | 1.016(0.941-1.096) |
| CD27 on CD24+ CD27+ B cell | Inverse variance weighted | 11 | 0.000 | 0.000 | 0.319 | 1.000 | 1.000(0.999-1.000) |
| Terminally Differentiated CD4-CD8- T cell %CD4-CD8- T cell | Wald ratio | 1 | 0.001 | 0.001 | 0.046 | 1.000 | 1.001(1.000-1.002) |
| Monocyte Absolute Count | Wald ratio | 1 | -0.017 | 0.216 | 0.938 | 1.000 | 0.983(0.645-1.500) |
| CD28+ CD45RA- CD8dim T cell %CD8dim T cell | Wald ratio | 1 | 0.000 | 0.000 | 0.617 | 1.000 | 1.000(1.000-1.000) |
| PDL-1 on CD14+ CD16- monocyte | Wald ratio | 1 | 0.020 | 0.219 | 0.929 | 1.000 | 1.020(0.664-1.567) |
| CD80 on CD62L+ myeloid Dendritic Cell | Inverse variance weighted | 3 | -0.039 | 0.180 | 0.830 | 1.000 | 0.962(0.676-1.369) |
| Effector Memory CD4-CD8- T cell %CD4-CD8- T cell | Inverse variance weighted | 2 | 0.181 | 0.253 | 0.474 | 1.000 | 1.198(0.730-1.967) |
| CD16-CD56 on Natural Killer | Inverse variance weighted | 7 | 0.000 | 0.000 | 0.999 | 1.000 | 1.000(0.999-1.001) |
| HLA DR++ monocyte Absolute Count | Wald ratio | 1 | 0.165 | 0.214 | 0.441 | 1.000 | 1.180(0.775-1.795) |
| CD45RA- CD28- CD8+ T cell Absolute Count | Inverse variance weighted | 122 | 0.000 | 0.000 | 0.092 | 1.000 | 1.000(1.000-1.000) |
| CD3 on activated & secreting CD4 regulatory T cell | Inverse variance weighted | 4 | 0.000 | 0.000 | 0.735 | 1.000 | 1.000(1.000-1.000) |
| IgD on IgD+ CD24- B cell | Inverse variance weighted | 5 | -0.014 | 0.059 | 0.819 | 1.000 | 0.987(0.878-1.108) |
| CD25 on IgD+ CD24- B cell | Inverse variance weighted | 5 | 0.119 | 0.077 | 0.122 | 1.000 | 1.126(0.969-1.308) |
| Granulocytic Myeloid-Derived Suppressor Cells Absolute Count | Inverse variance weighted | 2 | -0.006 | 0.101 | 0.951 | 1.000 | 0.994(0.815-1.212) |
| IgD on IgD+ CD38+ B cell | Inverse variance weighted | 3 | 0.013 | 0.065 | 0.840 | 1.000 | 1.013(0.891-1.152) |
| IgD+ CD38- B cell %B cell | Wald ratio | 1 | 0.265 | 0.235 | 0.259 | 1.000 | 1.304(0.822-2.068) |
| PDL-1 on CD14- CD16- | Wald ratio | 1 | 0.281 | 0.223 | 0.207 | 1.000 | 1.325(0.856-2.052) |
| HLA DR on CD33+ HLA DR+ CD14- | Inverse variance weighted | 4 | -0.117 | 0.051 | 0.022 | 1.000 | 0.889(0.804-0.984) |
| CD33+ HLA DR+ CD14dim Absolute Count | Inverse variance weighted | 2 | -0.058 | 0.108 | 0.595 | 1.000 | 0.944(0.763-1.168) |
| CD38 on IgD+ CD38+ B cell | Inverse variance weighted | 3 | 0.031 | 0.167 | 0.852 | 1.000 | 1.032(0.744-1.430) |
| HLA DR on CD14+ CD16- monocyte | Inverse variance weighted | 4 | -0.114 | 0.060 | 0.059 | 1.000 | 0.892(0.793-1.004) |
| CD3 on CD4+ T cell | Inverse variance weighted | 2 | 0.000 | 0.000 | 0.999 | 1.000 | 1.000(0.999-1.001) |
| HLA DR+ CD8+ T cell %T cell | Inverse variance weighted | 3 | 0.037 | 0.100 | 0.710 | 1.000 | 1.038(0.853-1.262) |
| CD80 on monocyte | Inverse variance weighted | 6 | 0.001 | 0.001 | 0.355 | 1.000 | 1.001(0.999-1.002) |
| CD40 on CD14+ CD16- monocyte | Inverse variance weighted | 4 | 0.000 | 0.000 | 0.315 | 1.000 | 1.000(1.000-1.001) |
| CD38 on IgD+ CD24- B cell | Inverse variance weighted | 2 | -0.173 | 0.114 | 0.129 | 1.000 | 0.841(0.673-1.052) |
| CD28+ CD4-CD8- T cell %T cell | Wald ratio | 1 | 0.000 | 0.001 | 1.000 | 1.000 | 1.000(0.999-1.001) |
| BAFF-R on memory B cell | Inverse variance weighted | 9 | 0.016 | 0.089 | 0.855 | 1.000 | 1.016(0.853-1.211) |
| CD4 regulatory T cell %CD4+ T cell | Inverse variance weighted | 2 | 0.355 | 0.149 | 0.017 | 1.000 | 1.425(1.065-1.908) |
| CD3 on activated CD4 regulatory T cell | Inverse variance weighted | 4 | 0.000 | 0.000 | 0.739 | 1.000 | 1.000(1.000-1.000) |
| CD33+ HLA DR+ CD14- Absolute Count | Wald ratio | 1 | -0.001 | 0.084 | 0.987 | 1.000 | 0.999(0.847-1.177) |
| CD8 on CD39+ CD8+ T cell | Inverse variance weighted | 4 | 0.000 | 0.000 | 0.999 | 1.000 | 1.000(0.999-1.001) |
| CD4 on CD39+ resting CD4 regulatory T cell | Wald ratio | 1 | 0.111 | 0.186 | 0.550 | 1.000 | 1.118(0.776-1.609) |
| CD14 on CD33dim HLA DR+ CD11b+ | Wald ratio | 1 | 0.000 | 0.000 | 0.317 | 1.000 | 1.000(0.999-1.000) |
| CD80 on CD62L+ plasmacytoid Dendritic Cell | Inverse variance weighted | 2 | -0.046 | 0.098 | 0.637 | 1.000 | 0.955(0.788-1.157) |
| BAFF-R on IgD+ CD24- B cell | Inverse variance weighted | 7 | -0.001 | 0.001 | 0.375 | 1.000 | 0.999(0.998-1.001) |
| HLA DR+ CD4+ T cell %lymphocyte | Inverse variance weighted | 2 | 0.236 | 0.251 | 0.347 | 1.000 | 1.266(0.774-2.069) |
| CD39 on CD39+ CD8+ T cell | Inverse variance weighted | 2 | 0.088 | 0.117 | 0.454 | 1.000 | 1.092(0.868-1.374) |
| CD4 on CD39+ activated CD4 regulatory T cell | Inverse variance weighted | 3 | 0.164 | 0.185 | 0.377 | 1.000 | 1.178(0.819-1.693) |
| CD39+ resting CD4 regulatory T cell Absolute Count | Inverse variance weighted | 10 | 0.000 | 0.000 | 0.453 | 1.000 | 1.000(1.000-1.000) |
| CD25++ CD45RA- CD4 not regulatory T cell %T cell | Inverse variance weighted | 2 | 0.000 | 0.000 | 0.634 | 1.000 | 1.000(1.000-1.000) |
| HLA DR on plasmacytoid Dendritic Cell | Inverse variance weighted | 7 | -0.033 | 0.045 | 0.463 | 1.000 | 0.968(0.886-1.056) |
| CD28 on CD28+ CD4-CD8- T cell | Inverse variance weighted | 3 | 0.017 | 0.104 | 0.873 | 1.000 | 1.017(0.829-1.246) |
| CD86+ myeloid Dendritic Cell %Dendritic Cell | Inverse variance weighted | 3 | 0.000 | 0.000 | 0.691 | 1.000 | 1.000(1.000-1.000) |
| CD45 on granulocyte | Wald ratio | 1 | 0.000 | 0.001 | 1.000 | 1.000 | 1.000(0.999-1.001) |
| CD8+ T cell Absolute Count | Wald ratio | 1 | -0.359 | 0.275 | 0.192 | 1.000 | 0.699(0.407-1.198) |
| IgD+ B cell %Lymphocyte | Inverse variance weighted | 2 | 0.123 | 0.191 | 0.518 | 1.000 | 1.131(0.778-1.644) |
| CD28 on activated CD4 regulatory T cell | Wald ratio | 1 | -0.042 | 0.114 | 0.713 | 1.000 | 0.959(0.768-1.198) |
| HLA DR+ Natural Killer %Natural Killer | Inverse variance weighted | 5 | 0.000 | 0.000 | 0.668 | 1.000 | 1.000(1.000-1.000) |
| CD3 on Terminally Differentiated CD4+ T cell | Wald ratio | 1 | 0.000 | 0.000 | 1.000 | 1.000 | 1.000(0.999-1.001) |
| SSC-A on T cell | Wald ratio | 1 | 0.191 | 0.250 | 0.444 | 1.000 | 1.211(0.742-1.978) |
| HLA DR on monocyte | Inverse variance weighted | 5 | -0.121 | 0.064 | 0.057 | 1.000 | 0.886(0.782-1.004) |
| CD45 on CD33- HLA DR- | Inverse variance weighted | 2 | 0.000 | 0.001 | 0.997 | 1.000 | 1.000(0.999-1.001) |
| CD45 on Natural Killer T | Wald ratio | 1 | -0.109 | 0.324 | 0.737 | 1.000 | 0.897(0.475-1.693) |
| CD3- lymphocyte Absolute Count | Wald ratio | 1 | 0.050 | 0.115 | 0.660 | 1.000 | 1.052(0.840-1.317) |
| CD28 on CD4+ T cell | Inverse variance weighted | 2 | 0.007 | 0.086 | 0.932 | 1.000 | 1.007(0.850-1.193) |
| CD4 on CD28+ CD4+ T cell | Wald ratio | 1 | 0.043 | 0.161 | 0.791 | 1.000 | 1.044(0.761-1.432) |
| CD3 on Effector Memory CD8+ T cell | Inverse variance weighted | 3 | 0.000 | 0.000 | 0.573 | 1.000 | 1.000(0.999-1.000) |
| CD127 on CD28+ CD45RA- CD8+ T cell | Wald ratio | 1 | 0.069 | 0.102 | 0.501 | 1.000 | 1.071(0.877-1.308) |
| CD3 on Central Memory CD4+ T cell | Inverse variance weighted | 4 | 0.000 | 0.000 | 0.719 | 1.000 | 1.000(1.000-1.000) |
| CD45RA- CD4+ T cell %T cell | Wald ratio | 1 | -0.001 | 0.001 | 0.046 | 1.000 | 0.999(0.998-1.000) |
| Switched memory B cell %lymphocyte | Inverse variance weighted | 3 | 0.107 | 0.125 | 0.389 | 1.000 | 1.113(0.872-1.422) |
| CD39+ CD4+ T cell Absolute Count | Inverse variance weighted | 5 | 0.029 | 0.062 | 0.642 | 1.000 | 1.029(0.911-1.163) |
| CD38 on CD3- CD19- | Wald ratio | 1 | 0.054 | 0.161 | 0.740 | 1.000 | 1.055(0.769-1.448) |
| CD3 on CD45RA- CD4+ T cell | Inverse variance weighted | 3 | 0.000 | 0.000 | 0.706 | 1.000 | 1.000(1.000-1.000) |
| CD25++ CD8+ T cell %T cell | Inverse variance weighted | 2 | 0.000 | 0.000 | 0.340 | 1.000 | 1.000(0.999-1.000) |
| IgD- CD27- B cell %B cell | Inverse variance weighted | 3 | -0.022 | 0.112 | 0.843 | 1.000 | 0.978(0.786-1.218) |
| Immature Myeloid-Derived Suppressor Cells Absolute Count | Inverse variance weighted | 2 | -0.099 | 0.117 | 0.399 | 1.000 | 0.906(0.720-1.140) |
| CD127 on CD8+ T cell | Wald ratio | 1 | -0.113 | 0.173 | 0.514 | 1.000 | 0.893(0.637-1.253) |
| BAFF-R on CD20- CD38- B cell | Wald ratio | 1 | 0.002 | 0.104 | 0.984 | 1.000 | 1.002(0.818-1.228) |
| CD8 on HLA DR+ CD8+ T cell | Wald ratio | 1 | 0.000 | 0.000 | 1.000 | 1.000 | 1.000(0.999-1.001) |
| Immature Myeloid-Derived Suppressor Cells %CD33dim HLA DR- CD66b- | Inverse variance weighted | 2 | 0.000 | 0.000 | 0.999 | 1.000 | 1.000(1.000-1.000) |
| CD25 on IgD- CD27- B cell | Inverse variance weighted | 2 | 0.059 | 0.223 | 0.792 | 1.000 | 1.061(0.685-1.643) |
| HLA DR on CD33+ HLA DR+ CD14dim | Inverse variance weighted | 4 | -0.175 | 0.075 | 0.020 | 1.000 | 0.840(0.724-0.973) |
| CD64 on CD14- CD16+ monocyte | Inverse variance weighted | 2 | 0.001 | 0.001 | 0.327 | 1.000 | 1.001(0.999-1.002) |
| CD33dim HLA DR- Absolute Count | Inverse variance weighted | 2 | 0.027 | 0.037 | 0.456 | 1.000 | 1.028(0.957-1.104) |
| CD62L on CD62L+ myeloid Dendritic Cell | Inverse variance weighted | 2 | 0.000 | 0.000 | 0.999 | 1.000 | 1.000(0.999-1.001) |
| IgD on IgD+ CD38- B cell | Wald ratio | 1 | 0.041 | 0.106 | 0.699 | 1.000 | 1.042(0.846-1.282) |
| CD14- CD16+ monocyte Absolute Count | Wald ratio | 1 | 0.000 | 0.000 | 1.000 | 1.000 | 1.000(1.000-1.000) |
| IgD- CD38+ B cell %B cell | Wald ratio | 1 | -0.001 | 0.001 | 0.317 | 1.000 | 0.999(0.998-1.001) |
| HLA DR on HLA DR+ CD4+ T cell | Wald ratio | 1 | -0.251 | 0.197 | 0.202 | 1.000 | 0.778(0.529-1.144) |
| CD3 on CD28+ CD45RA- CD8+ T cell | Inverse variance weighted | 3 | 0.000 | 0.000 | 0.999 | 1.000 | 1.000(0.999-1.001) |
| CD27 on IgD- CD38dim B cell | Inverse variance weighted | 9 | 0.000 | 0.000 | 0.320 | 1.000 | 1.000(0.999-1.000) |
| Central Memory CD4+ T cell Absolute Count | Wald ratio | 1 | -0.543 | 0.313 | 0.082 | 1.000 | 0.581(0.315-1.072) |
| CD28- CD8+ T cell %CD8+ T cell | Inverse variance weighted | 3 | 0.338 | 0.233 | 0.146 | 1.000 | 1.402(0.889-2.212) |
| HLA DR on CD33- HLA DR+ | Wald ratio | 1 | -0.070 | 0.066 | 0.287 | 1.000 | 0.932(0.819-1.061) |
| HVEM on Terminally Differentiated CD4+ T cell | Wald ratio | 1 | 0.000 | 0.000 | 0.317 | 1.000 | 1.000(1.000-1.001) |
| Basophil Absolute Count | Inverse variance weighted | 3 | 0.021 | 0.032 | 0.520 | 1.000 | 1.021(0.959-1.087) |
| CD27 on unswitched memory B cell | Inverse variance weighted | 10 | -0.001 | 0.001 | 0.320 | 1.000 | 0.999(0.998-1.001) |
| CD25++ CD45RA+ CD4 not regulatory T cell %CD4+ T cell | Inverse variance weighted | 4 | 0.000 | 0.000 | 0.524 | 1.000 | 1.000(1.000-1.000) |
| CD86+ plasmacytoid Dendritic Cell %Dendritic Cell | Wald ratio | 1 | 0.000 | 0.000 | 1.000 | 1.000 | 1.000(0.999-1.001) |
| CD33 on CD33dim HLA DR+ CD11b+ | Inverse variance weighted | 6 | 0.000 | 0.000 | 0.315 | 1.000 | 1.000(0.999-1.000) |
| CD39 on CD39+ activated CD4 regulatory T cell | Inverse variance weighted | 5 | 0.019 | 0.030 | 0.537 | 1.000 | 1.019(0.960-1.081) |
| Natural Killer T Absolute Count | Inverse variance weighted | 5 | 0.043 | 0.169 | 0.797 | 1.000 | 1.044(0.750-1.453) |
| CD16-CD56 on HLA DR+ Natural Killer | Inverse variance weighted | 2 | 0.000 | 0.000 | 0.999 | 1.000 | 1.000(0.999-1.001) |
| CD27 on IgD+ CD38- unswitched memory B cell | Inverse variance weighted | 5 | 0.036 | 0.058 | 0.529 | 1.000 | 1.037(0.926-1.161) |
| CD24 on IgD+ CD38+ B cell | Inverse variance weighted | 2 | 0.046 | 0.190 | 0.808 | 1.000 | 1.047(0.721-1.522) |
| CD8 on Central Memory CD8+ T cell | Inverse variance weighted | 2 | 0.000 | 0.001 | 0.998 | 1.000 | 1.000(0.998-1.002) |
| CD33 on Monocytic Myeloid-Derived Suppressor Cells | Inverse variance weighted | 3 | -0.006 | 0.027 | 0.838 | 1.000 | 0.994(0.943-1.049) |
| CD39 on CD39+ CD4+ T cell | Inverse variance weighted | 3 | 0.070 | 0.064 | 0.275 | 1.000 | 1.072(0.946-1.216) |
| CD123 on CD62L+ plasmacytoid Dendritic Cell | Inverse variance weighted | 2 | -0.043 | 0.092 | 0.642 | 1.000 | 0.958(0.799-1.148) |
| CD3 on Terminally Differentiated CD8+ T cell | Wald ratio | 1 | 0.000 | 0.000 | 1.000 | 1.000 | 1.000(0.999-1.001) |
| CD3 on naive CD8+ T cell | Inverse variance weighted | 7 | 0.000 | 0.000 | 1.000 | 1.000 | 1.000(1.000-1.000) |
| Effector Memory CD8+ T cell Absolute Count | Wald ratio | 1 | 0.050 | 0.079 | 0.525 | 1.000 | 1.052(0.900-1.228) |
| CD25 on memory B cell | Inverse variance weighted | 3 | -0.051 | 0.075 | 0.499 | 1.000 | 0.951(0.821-1.101) |
| CD24 on IgD+ CD24+ B cell | Inverse variance weighted | 2 | -0.033 | 0.177 | 0.851 | 1.000 | 0.967(0.684-1.368) |
| FSC-A on granulocyte | Wald ratio | 1 | -0.430 | 0.239 | 0.072 | 1.000 | 0.651(0.408-1.039) |
| CD39 on monocyte | Inverse variance weighted | 2 | -0.273 | 0.180 | 0.129 | 1.000 | 0.761(0.535-1.082) |
| CD20 on naive-mature B cell | Inverse variance weighted | 5 | -0.112 | 0.102 | 0.269 | 1.000 | 0.894(0.732-1.091) |
| CD33dim HLA DR+ CD11b- %CD33dim HLA DR+ | Inverse variance weighted | 3 | 0.000 | 0.000 | 0.430 | 1.000 | 1.000(1.000-1.000) |
| CD25 on IgD+ CD38dim B cell | Inverse variance weighted | 3 | 0.113 | 0.107 | 0.288 | 1.000 | 1.120(0.909-1.380) |
| CD25 on CD45RA+ CD4 not regulatory T cell | Wald ratio | 1 | -0.007 | 0.139 | 0.962 | 1.000 | 0.993(0.757-1.303) |
| CD8dim T cell Absolute Count | Inverse variance weighted | 3 | -0.331 | 0.152 | 0.029 | 1.000 | 0.718(0.533-0.968) |
| CD27 on IgD- CD38+ B cell | Inverse variance weighted | 2 | -0.020 | 0.150 | 0.895 | 1.000 | 0.980(0.730-1.316) |
| CD39+ CD8+ T cell Absolute Count | Inverse variance weighted | 6 | -0.002 | 0.062 | 0.969 | 1.000 | 0.998(0.883-1.127) |
| HLA DR+ T cell Absolute Count | Inverse variance weighted | 3 | 0.133 | 0.169 | 0.430 | 1.000 | 1.142(0.821-1.590) |
| Natural Killer T %T cell | Inverse variance weighted | 5 | 0.001 | 0.115 | 0.995 | 1.000 | 1.001(0.799-1.254) |
| HLA DR+ CD8+ T cell Absolute Count | Inverse variance weighted | 3 | 0.128 | 0.177 | 0.470 | 1.000 | 1.136(0.804-1.606) |
| CCR2 on CD14- CD16- | Inverse variance weighted | 3 | 0.223 | 0.135 | 0.099 | 1.000 | 1.249(0.959-1.628) |
| T cell Absolute Count | Inverse variance weighted | 2 | 0.383 | 0.780 | 0.623 | 1.000 | 1.467(0.318-6.772) |
| Memory B cell Absolute Count | Wald ratio | 1 | 0.228 | 0.432 | 0.598 | 1.000 | 1.256(0.539-2.927) |
| IgD on IgD+ CD24+ B cell | Inverse variance weighted | 5 | -0.051 | 0.132 | 0.700 | 1.000 | 0.950(0.733-1.231) |
| FSC-A on plasmacytoid Dendritic Cell | Inverse variance weighted | 4 | -0.059 | 0.124 | 0.634 | 1.000 | 0.943(0.740-1.202) |
| HLA DR on Dendritic Cell | Inverse variance weighted | 5 | 0.000 | 0.000 | 0.997 | 1.000 | 1.000(0.999-1.001) |
| Effector Memory CD4-CD8- T cell %T cell | Inverse variance weighted | 3 | 0.000 | 0.000 | 1.000 | 1.000 | 1.000(0.999-1.001) |
| SSC-A on granulocyte | Inverse variance weighted | 2 | -0.277 | 0.137 | 0.043 | 1.000 | 0.758(0.579-0.992) |
| CD3 on CD28+ CD45RA+ CD8+ T cell | Inverse variance weighted | 4 | 0.000 | 0.000 | 0.627 | 1.000 | 1.000(1.000-1.000) |
| CD62L- Dendritic Cell Absolute Count | Inverse variance weighted | 4 | 0.000 | 0.000 | 0.662 | 1.000 | 1.000(1.000-1.000) |
| CD25 on activated & secreting CD4 regulatory T cell | Wald ratio | 1 | 0.136 | 0.226 | 0.547 | 1.000 | 1.146(0.736-1.785) |
| CD8+ Natural Killer T %T cell | Wald ratio | 1 | 0.298 | 0.250 | 0.234 | 1.000 | 1.347(0.825-2.201) |
| CD4-CD8- T cell %T cell | Inverse variance weighted | 3 | -0.193 | 0.165 | 0.241 | 1.000 | 0.824(0.597-1.139) |
| CD28+ CD45RA- CD8dim T cell Absolute Count | Inverse variance weighted | 2 | 0.000 | 0.000 | 0.618 | 1.000 | 1.000(1.000-1.000) |
| CD45 on CD8+ T cell | Inverse variance weighted | 2 | 0.000 | 0.001 | 0.995 | 1.000 | 1.000(0.997-1.003) |
| CD4 regulatory T cell %T cell | Wald ratio | 1 | 0.272 | 0.232 | 0.240 | 1.000 | 1.313(0.834-2.068) |
| B cell Absolute Count | Wald ratio | 1 | 0.070 | 0.236 | 0.768 | 1.000 | 1.072(0.676-1.701) |
| Terminally Differentiated CD8+ T cell Absolute Count | Wald ratio | 1 | 0.001 | 0.001 | 0.046 | 1.000 | 1.001(1.000-1.002) |
| Activated & resting CD4 regulatory T cell Absolute Count | Wald ratio | 1 | 0.000 | 0.001 | 1.000 | 1.000 | 1.000(0.999-1.001) |
| CD3 on HLA DR+ T cell | Inverse variance weighted | 2 | 0.000 | 0.000 | 0.999 | 1.000 | 1.000(0.999-1.001) |
| CD25 on unswitched memory B cell | Inverse variance weighted | 3 | -0.051 | 0.078 | 0.514 | 1.000 | 0.950(0.815-1.107) |
| SSC-A on plasmacytoid Dendritic Cell | Inverse variance weighted | 4 | -0.081 | 0.118 | 0.488 | 1.000 | 0.922(0.732-1.161) |
| Dendritic Cell Absolute Count | Inverse variance weighted | 2 | 0.000 | 0.000 | 0.767 | 1.000 | 1.000(0.999-1.001) |
| CD19 on memory B cell | Inverse variance weighted | 2 | 0.014 | 0.241 | 0.954 | 1.000 | 1.014(0.632-1.626) |
| CD19 on IgD+ B cell | Inverse variance weighted | 2 | -0.008 | 0.188 | 0.967 | 1.000 | 0.992(0.686-1.435) |
| CCR2 on plasmacytoid Dendritic Cell | Inverse variance weighted | 2 | 0.090 | 0.074 | 0.221 | 1.000 | 1.094(0.947-1.264) |
| CD14+ CD16- monocyte %monocyte | Inverse variance weighted | 2 | 0.000 | 0.000 | 0.714 | 1.000 | 1.000(1.000-1.000) |
| CD45RA+ CD28- CD8+ T cell Absolute Count | Inverse variance weighted | 373 | 0.000 | 0.000 | 0.114 | 1.000 | 1.000(1.000-1.000) |
| IgD+ CD38+ B cell Absolute Count | Inverse variance weighted | 2 | -0.214 | 0.284 | 0.450 | 1.000 | 0.807(0.463-1.407) |
| CD4-CD8- T cell %leukocyte | Inverse variance weighted | 4 | -0.185 | 0.136 | 0.174 | 1.000 | 0.831(0.636-1.085) |
| CD25 on IgD+ B cell | Inverse variance weighted | 2 | 0.128 | 0.154 | 0.405 | 1.000 | 1.137(0.840-1.538) |
| BAFF-R on IgD+ B cell | Inverse variance weighted | 8 | -0.001 | 0.001 | 0.461 | 1.000 | 0.999(0.998-1.001) |
| CD20 on memory B cell | Inverse variance weighted | 4 | -0.023 | 0.117 | 0.846 | 1.000 | 0.977(0.777-1.230) |
| HLA DR on CD14- CD16- | Inverse variance weighted | 3 | 0.029 | 0.102 | 0.780 | 1.000 | 1.029(0.842-1.258) |
| CD33 on CD33dim HLA DR+ CD11b- | Inverse variance weighted | 6 | 0.000 | 0.000 | 0.316 | 1.000 | 1.000(0.999-1.000) |
| CD19 on IgD+ CD38dim B cell | Inverse variance weighted | 3 | -0.002 | 0.162 | 0.990 | 1.000 | 0.998(0.727-1.370) |
| CD62L- Dendritic Cell %Dendritic Cell | Inverse variance weighted | 4 | 0.000 | 0.000 | 0.830 | 1.000 | 1.000(1.000-1.001) |
| HVEM on Effector Memory CD4+ T cell | Wald ratio | 1 | 0.000 | 0.000 | 0.317 | 1.000 | 1.000(1.000-1.001) |
| CD11b on CD33dim HLA DR- | Wald ratio | 1 | -0.201 | 0.093 | 0.031 | 1.000 | 0.818(0.682-0.982) |
| CD4 on naive CD4+ T cell | Inverse variance weighted | 3 | -0.091 | 0.126 | 0.469 | 1.000 | 0.913(0.713-1.169) |
| CD11b on basophil | Wald ratio | 1 | -0.195 | 0.090 | 0.031 | 1.000 | 0.823(0.689-0.982) |
| CD16 on CD14+ CD16+ monocyte | Inverse variance weighted | 3 | 0.000 | 0.000 | 0.905 | 1.000 | 1.000(1.000-1.000) |
| CD4 on CD4 regulatory T cell | Wald ratio | 1 | 0.036 | 0.136 | 0.791 | 1.000 | 1.037(0.795-1.353) |
| HVEM on CD8+ T cell | Wald ratio | 1 | 0.000 | 0.000 | 0.317 | 1.000 | 1.000(1.000-1.001) |
| IgD+ CD24+ B cell %lymphocyte | Inverse variance weighted | 2 | 0.078 | 0.179 | 0.662 | 1.000 | 1.081(0.762-1.535) |
| CD45 on HLA DR+ CD4+ T cell | Wald ratio | 1 | 0.161 | 0.177 | 0.364 | 1.000 | 1.174(0.830-1.660) |
| CD20 on IgD+ CD38+ B cell | Inverse variance weighted | 5 | -0.017 | 0.084 | 0.843 | 1.000 | 0.984(0.835-1.159) |
| Plasma Blast-Plasma Cell %lymphocyte | Wald ratio | 1 | -0.197 | 0.247 | 0.425 | 1.000 | 0.821(0.507-1.332) |
| CD8+ Natural Killer T %lymphocyte | Wald ratio | 1 | 0.216 | 0.244 | 0.375 | 1.000 | 1.241(0.770-2.001) |
| BAFF-R on IgD+ CD38- B cell | Inverse variance weighted | 7 | -0.008 | 0.114 | 0.942 | 1.000 | 0.992(0.793-1.241) |
| CD19 on naive-mature B cell | Inverse variance weighted | 2 | 0.000 | 0.001 | 0.998 | 1.000 | 1.000(0.997-1.003) |
| CD45RA- CD28- CD8+ T cell %T cell | Inverse variance weighted | 33 | 0.000 | 0.000 | 0.336 | 1.000 | 1.000(1.000-1.000) |
| CD8dim Natural Killer T %lymphocyte | Inverse variance weighted | 6 | -0.025 | 0.093 | 0.784 | 1.000 | 0.975(0.813-1.169) |
| CD24+ CD27+ B cell %B cell | Inverse variance weighted | 3 | -0.019 | 0.138 | 0.891 | 1.000 | 0.981(0.748-1.287) |
| Granulocyte %leukocyte | Wald ratio | 1 | 0.550 | 0.281 | 0.051 | 1.000 | 1.733(0.999-3.006) |
| CD11c on monocyte | Inverse variance weighted | 2 | 0.000 | 0.000 | 0.317 | 1.000 | 1.000(1.000-1.001) |
| BAFF-R on IgD+ CD24+ B cell | Inverse variance weighted | 9 | 0.016 | 0.090 | 0.859 | 1.000 | 1.016(0.852-1.211) |
| CD62L- myeloid Dendritic Cell Absolute Count | Inverse variance weighted | 4 | 0.000 | 0.000 | 0.653 | 1.000 | 1.000(1.000-1.000) |
| CD4 on CD39+ secreting CD4 regulatory T cell | Inverse variance weighted | 2 | 0.112 | 0.104 | 0.280 | 1.000 | 1.119(0.913-1.372) |
| HLA DR on Hematopoietic Stem Cell | Inverse variance weighted | 3 | -0.083 | 0.140 | 0.551 | 1.000 | 0.920(0.699-1.210) |
| Secreting CD4 regulatory T cell %CD4+ T cell | Inverse variance weighted | 6 | 0.000 | 0.000 | 0.356 | 1.000 | 1.000(1.000-1.000) |
| IgD on unswitched memory B cell | Inverse variance weighted | 5 | 0.026 | 0.073 | 0.726 | 1.000 | 1.026(0.889-1.185) |
| BAFF-R on IgD- CD38- B cell | Inverse variance weighted | 8 | -0.015 | 0.077 | 0.842 | 1.000 | 0.985(0.847-1.145) |
| T cell %lymphocyte | Inverse variance weighted | 2 | -0.220 | 0.137 | 0.109 | 1.000 | 0.802(0.613-1.050) |
| CD25++ CD45RA+ CD4 not regulatory T cell Absolute Count | Inverse variance weighted | 3 | 0.000 | 0.000 | 0.375 | 1.000 | 1.000(1.000-1.000) |
| SSC-A on CD14+ monocyte | Inverse variance weighted | 5 | -0.003 | 0.071 | 0.965 | 1.000 | 0.997(0.867-1.146) |
| CD25++ CD4+ T cell %T cell | Wald ratio | 1 | 0.253 | 0.241 | 0.295 | 1.000 | 1.287(0.802-2.066) |
| Myeloid Dendritic Cell %Dendritic Cell | Inverse variance weighted | 2 | 0.000 | 0.000 | 0.703 | 1.000 | 1.000(1.000-1.000) |
| CD28 on CD39+ CD4+ T cell | Inverse variance weighted | 6 | 0.045 | 0.061 | 0.457 | 1.000 | 1.046(0.929-1.179) |
| IgD on IgD+ B cell | Inverse variance weighted | 2 | 0.033 | 0.080 | 0.677 | 1.000 | 1.034(0.884-1.208) |
| CD33- HLA DR- Absolute Count | Wald ratio | 1 | 0.216 | 0.168 | 0.198 | 1.000 | 1.241(0.894-1.723) |
| HLA DR+ T cell%lymphocyte | Inverse variance weighted | 5 | 0.161 | 0.123 | 0.193 | 1.000 | 1.174(0.922-1.495) |
| Hematopoietic Stem Cell Absolute Count | Inverse variance weighted | 2 | -0.025 | 0.069 | 0.720 | 1.000 | 0.976(0.853-1.116) |
| SSC-A on CD4+ T cell | Inverse variance weighted | 2 | 0.276 | 0.212 | 0.193 | 1.000 | 1.317(0.869-1.996) |
| Myeloid Dendritic Cell Absolute Count | Inverse variance weighted | 3 | 0.000 | 0.000 | 0.823 | 1.000 | 1.000(1.000-1.000) |
| B cell %CD3- lymphocyte | Inverse variance weighted | 3 | 0.024 | 0.059 | 0.692 | 1.000 | 1.024(0.911-1.150) |
| CD62L on CD62L+ plasmacytoid Dendritic Cell | Inverse variance weighted | 2 | 0.000 | 0.000 | 0.999 | 1.000 | 1.000(0.999-1.001) |
| CD4 on CD4+ T cell | Inverse variance weighted | 2 | 0.127 | 0.194 | 0.512 | 1.000 | 1.136(0.776-1.661) |
| CD25 on IgD+ CD38+ B cell | Inverse variance weighted | 3 | 0.199 | 0.116 | 0.085 | 1.000 | 1.221(0.973-1.532) |
| IgD- CD27- B cell %lymphocyte | Inverse variance weighted | 2 | -0.102 | 0.157 | 0.515 | 1.000 | 0.903(0.664-1.228) |
| CD25 on B cell | Inverse variance weighted | 3 | 0.113 | 0.097 | 0.244 | 1.000 | 1.120(0.926-1.355) |
| HVEM on T cell | Wald ratio | 1 | 0.000 | 0.000 | 0.317 | 1.000 | 1.000(1.000-1.001) |
| CD4+CD8+ T cell Absolute Count | Inverse variance weighted | 2 | 0.057 | 0.100 | 0.573 | 1.000 | 1.058(0.869-1.288) |
| CD20- CD38- B cell %B cell | Wald ratio | 1 | -0.104 | 0.150 | 0.486 | 1.000 | 0.901(0.671-1.209) |
| Activated & resting CD4 regulatory T cell %CD4 regulatory T cell | Inverse variance weighted | 5 | 0.000 | 0.000 | 0.643 | 1.000 | 1.000(1.000-1.000) |
| CD14+ CD16+ monocyte %monocyte | Inverse variance weighted | 3 | 0.000 | 0.000 | 0.748 | 1.000 | 1.000(1.000-1.000) |
| CD62L on monocyte | Wald ratio | 1 | 0.000 | 0.000 | 0.317 | 1.000 | 1.000(0.999-1.000) |
| CD39+ CD8+ T cell %T cell | Inverse variance weighted | 7 | 0.013 | 0.068 | 0.848 | 1.000 | 1.013(0.887-1.156) |
| IgD+ CD38+ B cell %lymphocyte | Wald ratio | 1 | -0.688 | 0.402 | 0.087 | 1.000 | 0.502(0.228-1.105) |
| BAFF-R on switched memory B cell | Inverse variance weighted | 9 | -0.001 | 0.001 | 0.317 | 1.000 | 0.999(0.998-1.001) |
| CD45 on CD33+ HLA DR+ CD14- | Wald ratio | 1 | 0.000 | 0.001 | 0.617 | 1.000 | 1.000(0.999-1.001) |
| CD4 on Central Memory CD4+ T cell | Inverse variance weighted | 2 | -0.052 | 0.125 | 0.678 | 1.000 | 0.950(0.744-1.212) |
| FSC-A on Natural Killer T | Inverse variance weighted | 2 | -0.047 | 0.179 | 0.792 | 1.000 | 0.954(0.671-1.355) |
| CD24 on IgD+ CD38- B cell | Inverse variance weighted | 2 | -0.028 | 0.298 | 0.926 | 1.000 | 0.973(0.543-1.744) |
| CD19 on IgD+ CD24+ B cell | Inverse variance weighted | 3 | 0.019 | 0.156 | 0.905 | 1.000 | 1.019(0.751-1.383) |
| IgD+ CD38- B cell %lymphocyte | Wald ratio | 1 | 0.230 | 0.436 | 0.598 | 1.000 | 1.258(0.536-2.957) |
| PDL-1 on CD14+ CD16+ monocyte | Wald ratio | 1 | 0.001 | 0.001 | 0.317 | 1.000 | 1.001(0.999-1.002) |
| CD80 on granulocyte | Inverse variance weighted | 3 | -0.001 | 0.001 | 0.088 | 1.000 | 0.999(0.998-1.000) |
| BAFF-R on unswitched memory B cell | Inverse variance weighted | 9 | 0.016 | 0.089 | 0.860 | 1.000 | 1.016(0.853-1.209) |
| CD8 on CD28- CD8+ T cell | Inverse variance weighted | 2 | 0.000 | 0.000 | 0.999 | 1.000 | 1.000(0.999-1.001) |
| CD19 on IgD+ CD38+ B cell | Inverse variance weighted | 3 | -0.001 | 0.135 | 0.995 | 1.000 | 0.999(0.767-1.301) |
| CD3 on CD28- CD8+ T cell | Wald ratio | 1 | 0.000 | 0.000 | 1.000 | 1.000 | 1.000(0.999-1.001) |
| HLA DR on HLA DR+ T cell | Wald ratio | 1 | -0.173 | 0.135 | 0.202 | 1.000 | 0.841(0.646-1.097) |
| Natural Killer Absolute Count | Inverse variance weighted | 3 | 0.079 | 0.183 | 0.668 | 1.000 | 1.082(0.756-1.549) |
| SSC-A on Natural Killer | Wald ratio | 1 | -0.150 | 0.217 | 0.490 | 1.000 | 0.861(0.563-1.317) |
| CD4 on secreting CD4 regulatory T cell | Wald ratio | 1 | 0.043 | 0.160 | 0.791 | 1.000 | 1.043(0.762-1.429) |
| CD25 on IgD+ CD38- naive B cell | Inverse variance weighted | 3 | 0.166 | 0.106 | 0.116 | 1.000 | 1.181(0.960-1.452) |
| CD28+ CD45RA+ CD8+ T cell %T cell | Inverse variance weighted | 34 | -0.001 | 0.006 | 0.811 | 1.000 | 0.999(0.987-1.010) |
| CD19 on CD24+ CD27+ B cell | Inverse variance weighted | 2 | 0.012 | 0.227 | 0.957 | 1.000 | 1.012(0.648-1.580) |
| CD38 on naive-mature B cell | Inverse variance weighted | 3 | -0.157 | 0.091 | 0.085 | 1.000 | 0.855(0.716-1.022) |
| CD8dim Natural Killer T %T cell | Inverse variance weighted | 5 | 0.016 | 0.094 | 0.868 | 1.000 | 1.016(0.844-1.222) |
| CD86+ myeloid Dendritic Cell Absolute Count | Inverse variance weighted | 4 | 0.000 | 0.000 | 0.687 | 1.000 | 1.000(1.000-1.000) |
| Plasmacytoid Dendritic Cell Absolute Count | Wald ratio | 1 | 0.001 | 0.001 | 0.317 | 1.000 | 1.001(1.000-1.002) |
| CD39+ activated CD4 regulatory T cell %CD4 regulatory T cell | Inverse variance weighted | 5 | 0.039 | 0.071 | 0.586 | 1.000 | 1.039(0.904-1.195) |
| Unswitched memory B cell %lymphocyte | Inverse variance weighted | 3 | 0.173 | 0.170 | 0.309 | 1.000 | 1.189(0.852-1.660) |
| BAFF-R on B cell | Inverse variance weighted | 7 | -0.001 | 0.001 | 0.357 | 1.000 | 0.999(0.998-1.001) |
| CD25++ CD45RA- CD4 not regulatory T cell Absolute Count | Wald ratio | 1 | 0.235 | 0.224 | 0.295 | 1.000 | 1.265(0.815-1.963) |
| CD80 on plasmacytoid Dendritic Cell | Inverse variance weighted | 2 | -0.045 | 0.097 | 0.640 | 1.000 | 0.956(0.790-1.156) |
| CD25++ CD8+ T cell %CD8+ T cell | Wald ratio | 1 | 0.000 | 0.000 | 0.617 | 1.000 | 1.000(0.999-1.000) |
| CD25++ CD45RA+ CD4 not regulatory T cell %T cell | Inverse variance weighted | 4 | 0.000 | 0.000 | 0.524 | 1.000 | 1.000(1.000-1.000) |
| CD11c+ HLA DR++ monocyte %monocyte | Wald ratio | 1 | -0.382 | 0.200 | 0.056 | 1.000 | 0.682(0.461-1.010) |
| CD4+CD8+ T cell %leukocyte | Wald ratio | 1 | 0.083 | 0.107 | 0.438 | 1.000 | 1.086(0.881-1.340) |
| CD33+ HLA DR+ CD14dim %CD33+ HLA DR+ | Wald ratio | 1 | -0.187 | 0.215 | 0.385 | 1.000 | 0.829(0.544-1.265) |
| CD28 on resting CD4 regulatory T cell | Inverse variance weighted | 2 | -0.023 | 0.058 | 0.690 | 1.000 | 0.977(0.873-1.094) |
| BAFF-R on IgD- CD38dim B cell | Inverse variance weighted | 9 | -0.001 | 0.001 | 0.437 | 1.000 | 0.999(0.998-1.001) |
| CD25 on transitional B cell | Inverse variance weighted | 2 | 0.280 | 0.178 | 0.116 | 1.000 | 1.323(0.933-1.876) |
| Effector Memory CD4-CD8- T cell Absolute Count | Inverse variance weighted | 4 | 0.000 | 0.001 | 0.998 | 1.000 | 1.000(0.999-1.001) |
| CD20 on switched memory B cell | Inverse variance weighted | 6 | 0.094 | 0.078 | 0.230 | 1.000 | 1.098(0.942-1.280) |
| CD20 on IgD+ B cell | Inverse variance weighted | 5 | -0.194 | 0.102 | 0.056 | 1.000 | 0.823(0.674-1.005) |
| PDL-1 on CD14- CD16+ monocyte | Inverse variance weighted | 2 | 0.000 | 0.000 | 0.690 | 1.000 | 1.000(1.000-1.000) |
| CD40 on CD14- CD16+ monocyte | Inverse variance weighted | 8 | 0.000 | 0.000 | 0.398 | 1.000 | 1.000(1.000-1.000) |
| CD33 on CD33+ HLA DR+ CD14- | Inverse variance weighted | 3 | -0.004 | 0.026 | 0.875 | 1.000 | 0.996(0.947-1.048) |
| CD45 on CD33- HLA DR+ | Wald ratio | 1 | -0.143 | 0.138 | 0.300 | 1.000 | 0.867(0.662-1.135) |
| Naive CD4-CD8- T cell %T cell | Wald ratio | 1 | -0.117 | 0.371 | 0.752 | 1.000 | 0.890(0.430-1.839) |
| CD28+ CD45RA- CD8+ T cell %CD8+ T cell | Inverse variance weighted | 2 | 0.000 | 0.001 | 0.892 | 1.000 | 1.000(0.999-1.001) |
| CD4 on Effector Memory CD4+ T cell | Inverse variance weighted | 3 | 0.013 | 0.146 | 0.928 | 1.000 | 1.013(0.761-1.350) |
| CD20 on B cell | Inverse variance weighted | 4 | 0.040 | 0.105 | 0.704 | 1.000 | 1.041(0.847-1.278) |
| CCR2 on CD14+ CD16- monocyte | Inverse variance weighted | 2 | 0.019 | 0.102 | 0.850 | 1.000 | 1.020(0.834-1.246) |
| CD33 on CD33+ HLA DR+ CD14dim | Inverse variance weighted | 3 | -0.004 | 0.026 | 0.881 | 1.000 | 0.996(0.946-1.049) |
| CD33 on basophil | Inverse variance weighted | 7 | 0.000 | 0.000 | 0.320 | 1.000 | 1.000(1.000-1.001) |
| CD20 on IgD+ CD38- B cell | Inverse variance weighted | 4 | 0.061 | 0.122 | 0.618 | 1.000 | 1.063(0.837-1.349) |
| CD45RA- CD28- CD8+ T cell %CD8+ T cell | Inverse variance weighted | 8 | 0.000 | 0.000 | 0.136 | 1.000 | 1.000(1.000-1.000) |
| CD3 on Central Memory CD8+ T cell | Inverse variance weighted | 3 | 0.000 | 0.000 | 1.000 | 1.000 | 1.000(1.000-1.000) |
| HLA DR on HLA DR+ Natural Killer | Inverse variance weighted | 4 | 0.000 | 0.000 | 0.999 | 1.000 | 1.000(0.999-1.001) |
| CD4 on resting CD4 regulatory T cell | Wald ratio | 1 | 0.047 | 0.178 | 0.791 | 1.000 | 1.048(0.740-1.485) |
| CD62L- plasmacytoid Dendritic Cell %Dendritic Cell | Inverse variance weighted | 3 | 0.000 | 0.000 | 1.000 | 1.000 | 1.000(0.999-1.001) |
| CD28+ CD45RA- CD8dim T cell %T cell | Inverse variance weighted | 2 | 0.000 | 0.000 | 0.618 | 1.000 | 1.000(1.000-1.000) |
| CD8+ Natural Killer T Absolute Count | Wald ratio | 1 | 0.216 | 0.243 | 0.375 | 1.000 | 1.241(0.770-2.000) |
| CD38 on IgD- CD38dim B cell | Inverse variance weighted | 3 | -0.143 | 0.083 | 0.084 | 1.000 | 0.867(0.737-1.019) |
| CD4 on activated CD4 regulatory T cell | Wald ratio | 1 | 0.035 | 0.132 | 0.791 | 1.000 | 1.036(0.799-1.342) |
| HVEM on naive CD8+ T cell | Inverse variance weighted | 2 | -0.093 | 0.219 | 0.670 | 1.000 | 0.911(0.593-1.399) |
| HVEM on Central Memory CD4+ T cell | Wald ratio | 1 | 0.000 | 0.000 | 0.317 | 1.000 | 1.000(1.000-1.001) |
| CD28 on CD28+ CD45RA+ CD8+ T cell | Wald ratio | 1 | -0.015 | 0.076 | 0.848 | 1.000 | 0.985(0.849-1.144) |
| CD39+ CD8+ T cell %CD8+ T cell | Inverse variance weighted | 7 | -0.008 | 0.064 | 0.898 | 1.000 | 0.992(0.876-1.123) |
| BAFF-R on transitional B cell | Inverse variance weighted | 9 | -0.081 | 0.058 | 0.164 | 1.000 | 0.922(0.823-1.034) |
| CD27 on switched memory B cell | Inverse variance weighted | 10 | 0.000 | 0.000 | 0.319 | 1.000 | 1.000(0.999-1.000) |
| BAFF-R on IgD+ CD38dim B cell | Inverse variance weighted | 8 | -0.001 | 0.001 | 0.461 | 1.000 | 0.999(0.998-1.001) |
| HLA DR on CD33dim HLA DR+ CD11b- | Inverse variance weighted | 3 | -0.009 | 0.167 | 0.957 | 1.000 | 0.991(0.714-1.376) |
| CD11c on myeloid Dendritic Cell | Inverse variance weighted | 5 | 0.000 | 0.001 | 0.999 | 1.000 | 1.000(0.999-1.001) |
| CD39+ activated CD4 regulatory T cell Absolute Count | Inverse variance weighted | 5 | 0.032 | 0.064 | 0.615 | 1.000 | 1.033(0.911-1.171) |
| SSC-A on monocyte | Inverse variance weighted | 7 | 0.007 | 0.053 | 0.891 | 1.000 | 1.007(0.908-1.117) |
| CD45RA- CD4+ T cell Absolute Count | Wald ratio | 1 | -0.529 | 0.310 | 0.088 | 1.000 | 0.589(0.321-1.082) |
| CD4-CD8- Natural Killer T %lymphocyte | Inverse variance weighted | 6 | -0.015 | 0.100 | 0.877 | 1.000 | 0.985(0.810-1.198) |
| CD4 on monocyte | Wald ratio | 1 | 0.227 | 0.221 | 0.303 | 1.000 | 1.255(0.814-1.935) |
| CD4RA on Terminally Differentiated CD4+ T cell | Inverse variance weighted | 2 | 0.000 | 0.000 | 0.638 | 1.000 | 1.000(1.000-1.000) |
| CD19 on CD20- B cell | Wald ratio | 1 | -0.092 | 0.102 | 0.367 | 1.000 | 0.912(0.746-1.114) |
| Monocytic Myeloid-Derived Suppressor Cells Absolute Count | Inverse variance weighted | 4 | 0.076 | 0.064 | 0.230 | 1.000 | 1.079(0.953-1.222) |
| CD25 on CD39+ activated CD4 regulatory T cell | Wald ratio | 1 | -0.204 | 0.179 | 0.254 | 1.000 | 0.815(0.574-1.158) |
| CD19 on transitional B cell | Inverse variance weighted | 3 | -0.001 | 0.157 | 0.997 | 1.000 | 0.999(0.734-1.360) |
| CD28 on CD39+ resting CD4 regulatory T cell | Inverse variance weighted | 2 | 0.013 | 0.123 | 0.915 | 1.000 | 1.013(0.796-1.290) |
| CD62L- monocyte %monocyte | Inverse variance weighted | 3 | 0.000 | 0.000 | 0.449 | 1.000 | 1.000(1.000-1.001) |
| CD62L- myeloid Dendritic Cell %Dendritic Cell | Inverse variance weighted | 4 | 0.000 | 0.000 | 0.645 | 1.000 | 1.000(1.000-1.000) |
| CD25 on IgD- CD38dim B cell | Inverse variance weighted | 3 | 0.069 | 0.121 | 0.567 | 1.000 | 1.072(0.845-1.359) |
| CD25 on IgD- CD38- B cell | Inverse variance weighted | 3 | 0.012 | 0.110 | 0.912 | 1.000 | 1.012(0.815-1.257) |
| CD62L- monocyte Absolute Count | Inverse variance weighted | 3 | 0.000 | 0.000 | 0.318 | 1.000 | 1.000(1.000-1.001) |
| CD28+ CD45RA+ CD8dim T cell %T cell | Inverse variance weighted | 5 | 0.000 | 0.000 | 0.493 | 1.000 | 1.000(1.000-1.000) |
| HVEM on CD45RA- CD4+ T cell | Wald ratio | 1 | 0.000 | 0.000 | 0.317 | 1.000 | 1.000(1.000-1.001) |
| Naive-mature B cell %B cell | Wald ratio | 1 | -0.495 | 0.368 | 0.179 | 1.000 | 0.609(0.296-1.254) |
| CD25++ CD4+ T cell Absolute Count | Wald ratio | 1 | 0.282 | 0.269 | 0.295 | 1.000 | 1.325(0.782-2.245) |
| CD24 on switched memory B cell | Inverse variance weighted | 2 | 0.048 | 0.205 | 0.813 | 1.000 | 1.050(0.702-1.570) |
| CD8 on CD28+ CD45RA+ CD8+ T cell | Inverse variance weighted | 6 | 0.000 | 0.000 | 0.637 | 1.000 | 1.000(1.000-1.000) |
| IgD- CD27- B cell Absolute Count | Inverse variance weighted | 2 | 0.042 | 0.151 | 0.779 | 1.000 | 1.043(0.776-1.402) |
| CD62L- CD86+ myeloid Dendritic Cell %Dendritic Cell | Inverse variance weighted | 5 | 0.000 | 0.000 | 0.916 | 1.000 | 1.000(1.000-1.000) |
| CX3CR1 on CD14+ CD16+ monocyte | Inverse variance weighted | 2 | 0.000 | 0.000 | 0.317 | 1.000 | 1.000(0.999-1.000) |
| CD25++ CD4+ T cell %CD4+ T cell | Wald ratio | 1 | 0.220 | 0.210 | 0.295 | 1.000 | 1.246(0.825-1.881) |
| Terminally Differentiated CD8+ T cell %T cell | Inverse variance weighted | 2 | 0.001 | 0.001 | 0.046 | 1.000 | 1.001(1.000-1.002) |
| CD28+ CD45RA- CD8+ T cell %T cell | Inverse variance weighted | 3 | 0.039 | 0.054 | 0.477 | 1.000 | 1.039(0.935-1.156) |
| CD25 on CD39+ resting CD4 regulatory T cell | Wald ratio | 1 | 0.001 | 0.001 | 0.317 | 1.000 | 1.001(0.999-1.002) |
| Activated & secreting CD4 regulatory T cell %CD4 regulatory T cell | Inverse variance weighted | 8 | 0.000 | 0.000 | 0.643 | 1.000 | 1.000(1.000-1.000) |
| Central Memory CD4+ T cell %CD4+ T cell | Wald ratio | 1 | 0.000 | 0.001 | 1.000 | 1.000 | 1.000(0.999-1.001) |
| Central Memory CD8+ T cell Absolute Count | Wald ratio | 1 | 0.069 | 0.296 | 0.816 | 1.000 | 1.071(0.600-1.914) |
| CD25 on CD28+ CD4+ T cell | Wald ratio | 1 | 0.285 | 0.273 | 0.295 | 1.000 | 1.330(0.780-2.270) |
| CD3 on CD39+ secreting CD4 regulatory T cell | Inverse variance weighted | 3 | 0.000 | 0.000 | 0.651 | 1.000 | 1.000(1.000-1.000) |
| Memory B cell %lymphocyte | Inverse variance weighted | 2 | 0.002 | 0.190 | 0.993 | 1.000 | 1.002(0.691-1.453) |
| CD14+ CD16+ monocyte Absolute Count | Inverse variance weighted | 3 | 0.000 | 0.000 | 0.719 | 1.000 | 1.000(1.000-1.000) |
| CD3 on Effector Memory CD4+ T cell | Wald ratio | 1 | -0.046 | 0.117 | 0.697 | 1.000 | 0.955(0.759-1.202) |
| CD8 on Effector Memory CD8+ T cell | Inverse variance weighted | 4 | 0.000 | 0.000 | 0.996 | 1.000 | 1.000(0.999-1.001) |
| CD3 on CD4 regulatory T cell | Inverse variance weighted | 3 | 0.000 | 0.000 | 1.000 | 1.000 | 1.000(1.000-1.000) |
| B cell %lymphocyte | Inverse variance weighted | 2 | 0.081 | 0.173 | 0.642 | 1.000 | 1.084(0.772-1.522) |
| CD45RA on naive CD8+ T cell | Inverse variance weighted | 2 | 0.000 | 0.001 | 0.999 | 1.000 | 1.000(0.999-1.001) |
| BAFF-R on IgD- CD38+ B cell | Inverse variance weighted | 3 | -0.069 | 0.115 | 0.551 | 1.000 | 0.934(0.745-1.170) |
| CD39+ CD4+ T cell %T cell | Inverse variance weighted | 6 | 0.030 | 0.056 | 0.586 | 1.000 | 1.031(0.924-1.150) |
| CD27 on T cell | Wald ratio | 1 | 0.000 | 0.000 | 0.317 | 1.000 | 1.000(0.999-1.000) |
| CCR2 on myeloid Dendritic Cell | Inverse variance weighted | 3 | 0.000 | 0.000 | 1.000 | 1.000 | 1.000(1.000-1.000) |
| CD20 on IgD- CD27- B cell | Inverse variance weighted | 4 | 0.080 | 0.114 | 0.485 | 1.000 | 1.083(0.866-1.354) |
| CD40 on monocytes | Inverse variance weighted | 4 | 0.000 | 0.000 | 0.630 | 1.000 | 1.000(1.000-1.000) |
| CD28- CD8+ T cell Absolute Count | Inverse variance weighted | 2 | 0.315 | 0.259 | 0.224 | 1.000 | 1.370(0.825-2.276) |
| CD25 on CD24+ CD27+ B cell | Inverse variance weighted | 3 | -0.051 | 0.075 | 0.495 | 1.000 | 0.950(0.821-1.100) |
| Unswitched memory B cell Absolute Count | Inverse variance weighted | 2 | 0.211 | 0.304 | 0.488 | 1.000 | 1.235(0.681-2.239) |
| CD4+ T cell %leukocyte | Wald ratio | 1 | -0.054 | 0.326 | 0.869 | 1.000 | 0.947(0.500-1.795) |
| CD4-CD8- Natural Killer T Absolute Count | Inverse variance weighted | 5 | -0.018 | 0.126 | 0.889 | 1.000 | 0.982(0.767-1.258) |
| CD14- CD16- Absolute Count | Inverse variance weighted | 2 | -0.118 | 0.404 | 0.770 | 1.000 | 0.888(0.402-1.962) |
| Lymphocyte Absolute Count | Inverse variance weighted | 2 | 0.027 | 0.703 | 0.969 | 1.000 | 1.028(0.259-4.072) |
| CD33 on CD33dim HLA DR- | Inverse variance weighted | 6 | 0.000 | 0.000 | 0.319 | 1.000 | 1.000(1.000-1.001) |
| BAFF-R on naive-mature B cell | Inverse variance weighted | 7 | -0.001 | 0.001 | 0.375 | 1.000 | 0.999(0.998-1.001) |
| Naive CD8+ T cell %CD8+ T cell | Wald ratio | 1 | 0.238 | 0.305 | 0.435 | 1.000 | 1.269(0.698-2.308) |
| Effector Memory CD8+ T cell %T cell | Inverse variance weighted | 2 | 0.018 | 0.117 | 0.881 | 1.000 | 1.018(0.809-1.279) |
| CD3- lymphocyte %lymphocyte | Inverse variance weighted | 2 | 0.220 | 0.137 | 0.109 | 1.000 | 1.246(0.952-1.631) |
| Activated CD4 regulatory T cell %CD4 regulatory T cell | Inverse variance weighted | 3 | 0.054 | 0.058 | 0.354 | 1.000 | 1.055(0.942-1.183) |
| CD39 on granulocyte | Inverse variance weighted | 3 | -0.093 | 0.257 | 0.718 | 1.000 | 0.911(0.550-1.509) |
| CD4 on activated & secreting CD4 regulatory T cell | Wald ratio | 1 | 0.037 | 0.139 | 0.791 | 1.000 | 1.037(0.791-1.361) |
| FSC-A on CD8+ T cell | Wald ratio | 1 | -0.617 | 0.396 | 0.119 | 1.000 | 0.540(0.248-1.172) |
| CD28 on CD28+ CD4+ T cell | Inverse variance weighted | 2 | 0.007 | 0.082 | 0.934 | 1.000 | 1.007(0.858-1.182) |
| CD28 on CD39+ activated CD4 regulatory T cell | Wald ratio | 1 | -0.030 | 0.117 | 0.795 | 1.000 | 0.970(0.772-1.220) |
| CD27 on memory B cell | Inverse variance weighted | 8 | 0.028 | 0.051 | 0.589 | 1.000 | 1.028(0.930-1.137) |
| CD3 on HLA DR+ CD8+ T cell | Wald ratio | 1 | 0.000 | 0.000 | 1.000 | 1.000 | 1.000(0.999-1.001) |
| CD25++ CD8+ T cell Absolute Count | Inverse variance weighted | 3 | 0.000 | 0.000 | 0.317 | 1.000 | 1.000(0.999-1.000) |
| SSC-A on Natural Killer T | Inverse variance weighted | 2 | -0.259 | 0.305 | 0.396 | 1.000 | 0.772(0.425-1.404) |
| HLA DR+ CD4+ T cell %T cell | Wald ratio | 1 | 0.078 | 0.122 | 0.525 | 1.000 | 1.081(0.851-1.373) |
| CD3 on CD45RA+ CD4+ T cell | Inverse variance weighted | 5 | 0.000 | 0.000 | 0.726 | 1.000 | 1.000(1.000-1.000) |
| HLA DR on CD14+ CD16+ monocyte | Inverse variance weighted | 2 | -0.211 | 0.113 | 0.063 | 1.000 | 0.810(0.648-1.012) |
| IgD+ CD24+ B cell Absolute Count | Wald ratio | 1 | 0.201 | 0.381 | 0.598 | 1.000 | 1.222(0.580-2.577) |
| BAFF-R on IgD- CD27- B cell | Inverse variance weighted | 7 | -0.001 | 0.001 | 0.358 | 1.000 | 0.999(0.998-1.001) |
| CD19 on IgD+ CD24- B cell | Inverse variance weighted | 3 | 0.000 | 0.001 | 1.000 | 1.000 | 1.000(0.999-1.001) |
| Terminally Differentiated CD4-CD8- T cell %T cell | Wald ratio | 1 | 0.000 | 0.001 | 1.000 | 1.000 | 1.000(0.999-1.001) |
| Secreting CD4 regulatory T cell Absolute Count | Inverse variance weighted | 3 | 0.000 | 0.000 | 0.618 | 1.000 | 1.000(1.000-1.000) |
| CD45RA+ CD8+ T cell %CD8+ T cell | Inverse variance weighted | 4 | 0.000 | 0.001 | 0.994 | 1.000 | 1.000(0.998-1.002) |
| CD28+ CD4-CD8- T cell %CD4-CD8- T cell | Wald ratio | 1 | -0.520 | 0.353 | 0.140 | 1.000 | 0.594(0.298-1.187) |
| HLA DR on myeloid Dendritic Cell | Inverse variance weighted | 6 | 0.000 | 0.000 | 0.998 | 1.000 | 1.000(1.000-1.000) |
| CD123 on plasmacytoid Dendritic Cell | Inverse variance weighted | 2 | -0.042 | 0.092 | 0.645 | 1.000 | 0.959(0.801-1.147) |
| Memory B cell %B cell | Inverse variance weighted | 2 | 0.452 | 0.218 | 0.038 | 1.000 | 1.572(1.025-2.410) |
| CD39+ secreting CD4 regulatory T cell Absolute Count | Inverse variance weighted | 5 | 0.049 | 0.072 | 0.492 | 1.000 | 1.051(0.913-1.210) |
| CD28- CD4-CD8- T cell Absolute Count | Inverse variance weighted | 2 | 0.000 | 0.001 | 0.998 | 1.000 | 1.000(0.999-1.001) |
| T/B cell | Inverse variance weighted | 2 | -0.060 | 0.172 | 0.728 | 1.000 | 0.942(0.672-1.320) |
| CD3 on resting CD4 regulatory T cell | Inverse variance weighted | 5 | 0.000 | 0.000 | 0.791 | 1.000 | 1.000(1.000-1.000) |
| CD3- lymphocyte %leukocyte | Wald ratio | 1 | -0.577 | 0.392 | 0.140 | 1.000 | 0.561(0.261-1.209) |
| CD24 on IgD- CD38- B cell | Inverse variance weighted | 2 | -0.019 | 0.241 | 0.938 | 1.000 | 0.981(0.611-1.575) |
| CD25 on CD39+ CD4+ T cell | Inverse variance weighted | 6 | -0.025 | 0.039 | 0.529 | 1.000 | 0.976(0.903-1.054) |
| CD25 on CD39+ secreting CD4 regulatory T cell | Wald ratio | 1 | 0.122 | 0.203 | 0.547 | 1.000 | 1.130(0.759-1.683) |
| CD27 on IgD- CD38- B cell | Inverse variance weighted | 7 | 0.000 | 0.000 | 0.319 | 1.000 | 1.000(0.999-1.000) |
| BAFF-R on IgD+ CD38+ B cell | Inverse variance weighted | 5 | -0.001 | 0.001 | 0.326 | 1.000 | 0.999(0.998-1.001) |
| CD33dim HLA DR+ CD11b+ %CD33dim HLA DR+ | Inverse variance weighted | 3 | 0.000 | 0.000 | 0.431 | 1.000 | 1.000(1.000-1.000) |
| IgD+ CD24- B cell %B cell | Wald ratio | 1 | 0.120 | 0.168 | 0.473 | 1.000 | 1.128(0.812-1.568) |
| CD39+ resting CD4 regulatory T cell %CD4 regulatory T cell | Inverse variance weighted | 11 | 0.000 | 0.000 | 0.453 | 1.000 | 1.000(1.000-1.000) |
| Natural Killer T %lymphocyte | Inverse variance weighted | 7 | -0.054 | 0.090 | 0.551 | 1.000 | 0.948(0.794-1.131) |
| Terminally Differentiated CD4+ T cell %CD4+ T cell | Inverse variance weighted | 3 | 0.237 | 0.209 | 0.258 | 1.000 | 1.267(0.841-1.910) |
| CX3CR1 on CD14- CD16+ monocyte | Inverse variance weighted | 2 | 0.000 | 0.000 | 0.317 | 1.000 | 1.000(0.999-1.000) |
| CD4 on CD45RA+ CD4+ T cell | Inverse variance weighted | 3 | 0.036 | 0.166 | 0.828 | 1.000 | 1.037(0.749-1.434) |
| CD28- CD4-CD8- T cell %CD4-CD8- T cell | Wald ratio | 1 | 0.520 | 0.353 | 0.140 | 1.000 | 1.683(0.843-3.359) |
| Naive CD4+ T cell %CD4+ T cell | Wald ratio | 1 | 0.001 | 0.001 | 0.046 | 1.000 | 1.001(1.000-1.003) |
| Terminally Differentiated CD8+ T cell %CD8+ T cell | Inverse variance weighted | 4 | 0.000 | 0.000 | 0.182 | 1.000 | 1.000(1.000-1.001) |
| CD33- HLA DR+ Absolute Count | Wald ratio | 1 | -0.161 | 0.126 | 0.201 | 1.000 | 0.851(0.665-1.090) |
| CCR2 on CD62L+ plasmacytoid Dendritic Cell | Inverse variance weighted | 2 | 0.090 | 0.074 | 0.221 | 1.000 | 1.094(0.947-1.265) |
| CD25 on IgD+ CD38- B cell | Inverse variance weighted | 2 | 0.218 | 0.125 | 0.081 | 1.000 | 1.244(0.974-1.589) |
| CD11b on Monocytic Myeloid-Derived Suppressor Cells | Wald ratio | 1 | 0.000 | 0.000 | 0.317 | 1.000 | 1.000(1.000-1.001) |
| CD14- CD16+ monocyte %monocyte | Inverse variance weighted | 2 | 0.000 | 0.000 | 1.000 | 1.000 | 1.000(1.000-1.000) |
| CD28 on CD45RA+ CD4+ T cell | Inverse variance weighted | 2 | -0.031 | 0.077 | 0.685 | 1.000 | 0.969(0.834-1.126) |
| CD24 on CD24+ CD27+ B cell | Inverse variance weighted | 3 | 0.018 | 0.122 | 0.883 | 1.000 | 1.018(0.802-1.293) |
| CD24 on IgD- CD38dim B cell | Inverse variance weighted | 2 | 0.045 | 0.196 | 0.819 | 1.000 | 1.046(0.712-1.536) |
| CD3 on CD39+ CD4+ T cell | Inverse variance weighted | 3 | 0.000 | 0.000 | 0.701 | 1.000 | 1.000(1.000-1.000) |
| CD11b on CD33+ HLA DR+ CD14dim | Wald ratio | 1 | 0.000 | 0.000 | 0.317 | 1.000 | 1.000(1.000-1.001) |
| Activated & resting CD4 regulatory T cell %CD4+ T cell | Inverse variance weighted | 3 | 0.000 | 0.000 | 0.441 | 1.000 | 1.000(1.000-1.001) |
| CD45RA on resting CD4 regulatory T cell | Inverse variance weighted | 4 | 0.000 | 0.000 | 0.863 | 1.000 | 1.000(1.000-1.000) |
| FSC-A on HLA DR+ Natural Killer | Inverse variance weighted | 3 | 0.000 | 0.000 | 0.790 | 1.000 | 1.000(1.000-1.000) |
| CD24 on memory B cell | Inverse variance weighted | 2 | 0.047 | 0.197 | 0.810 | 1.000 | 1.048(0.712-1.543) |
| CD39 on CD39+ secreting CD4 regulatory T cell | Inverse variance weighted | 4 | 0.052 | 0.061 | 0.386 | 1.000 | 1.054(0.936-1.187) |
| Naive CD8+ T cell Absolute Count | Wald ratio | 1 | 0.000 | 0.001 | 1.000 | 1.000 | 1.000(0.998-1.002) |
| CD33+ HLA DR+ CD14- %CD33+ HLA DR+ | Wald ratio | 1 | 0.000 | 0.101 | 0.997 | 1.000 | 1.000(0.820-1.219) |
| CD8dim Natural Killer T Absolute Count | Inverse variance weighted | 5 | -0.015 | 0.108 | 0.887 | 1.000 | 0.985(0.797-1.217) |
| Basophil %CD33dim HLA DR- CD66b- | Inverse variance weighted | 3 | 0.013 | 0.097 | 0.892 | 1.000 | 1.013(0.837-1.226) |
| CD16 on CD14- CD16+ monocyte | Inverse variance weighted | 7 | 0.000 | 0.000 | 0.980 | 1.000 | 1.000(1.000-1.000) |
| CD8dim T cell %T cell | Inverse variance weighted | 2 | -0.257 | 0.158 | 0.103 | 1.000 | 0.773(0.567-1.054) |
| CD25 on naive-mature B cell | Inverse variance weighted | 4 | 0.106 | 0.080 | 0.183 | 1.000 | 1.112(0.951-1.300) |
| CD3 on CD39+ CD8+ T cell | Wald ratio | 1 | 0.000 | 0.000 | 1.000 | 1.000 | 1.000(0.999-1.001) |
| CD28+ CD45RA+ CD8dim T cell %CD8dim T cell | Inverse variance weighted | 4 | 0.000 | 0.000 | 0.393 | 1.000 | 1.000(1.000-1.000) |
| CD3 on CD39+ activated CD4 regulatory T cell | Inverse variance weighted | 5 | 0.000 | 0.000 | 0.740 | 1.000 | 1.000(1.000-1.000) |
| CD39+ secreting CD4 regulatory T cell %CD4 regulatory T cell | Inverse variance weighted | 7 | 0.036 | 0.068 | 0.592 | 1.000 | 1.037(0.908-1.184) |
| CD19 on IgD- CD38- B cell | Wald ratio | 1 | 0.138 | 0.223 | 0.536 | 1.000 | 1.148(0.741-1.778) |
| Lymphocyte %leukocyte | Wald ratio | 1 | -0.553 | 0.283 | 0.051 | 1.000 | 0.575(0.330-1.001) |
| CD38 on IgD+ CD38dim B cell | Inverse variance weighted | 4 | -0.134 | 0.076 | 0.076 | 1.000 | 0.874(0.754-1.014) |
| CCR2 on CD14+ CD16+ monocyte | Inverse variance weighted | 2 | 0.000 | 0.001 | 0.999 | 1.000 | 1.000(0.999-1.001) |
| HLA DR+ T cell%T cell | Inverse variance weighted | 4 | 0.122 | 0.131 | 0.351 | 1.000 | 1.130(0.874-1.462) |
| CD33 on Granulocytic Myeloid-Derived Suppressor Cells | Inverse variance weighted | 3 | -0.002 | 0.045 | 0.964 | 1.000 | 0.998(0.914-1.090) |
| CD8+ T cell %T cell | Wald ratio | 1 | 0.108 | 0.338 | 0.751 | 1.000 | 1.114(0.574-2.161) |
| HLA DR+ Natural Killer Absolute Count | Inverse variance weighted | 3 | 0.000 | 0.000 | 0.662 | 1.000 | 1.000(1.000-1.000) |
| SSC-A on myeloid Dendritic Cell | Inverse variance weighted | 5 | 0.000 | 0.000 | 0.995 | 1.000 | 1.000(0.999-1.001) |
| CD127 on CD28+ CD4+ T cell | Inverse variance weighted | 2 | 0.001 | 0.001 | 0.316 | 1.000 | 1.001(0.999-1.002) |
| CD39+ activated CD4 regulatory T cell %activated CD4 regulatory T cell | Inverse variance weighted | 10 | -0.002 | 0.029 | 0.949 | 1.000 | 0.998(0.942-1.057) |
| HLA DR+ CD8+ T cell %lymphocyte | Inverse variance weighted | 2 | 0.052 | 0.138 | 0.707 | 1.000 | 1.053(0.803-1.381) |
| CD33dim HLA DR+ CD11b- Absolute Count | Inverse variance weighted | 2 | 0.003 | 0.095 | 0.972 | 1.000 | 1.003(0.834-1.208) |
| Effector Memory CD8+ T cell %CD8+ T cell | Inverse variance weighted | 3 | 0.001 | 0.001 | 0.540 | 1.000 | 1.001(0.999-1.003) |
| CD33dim HLA DR+ CD11b+ Absolute Count | Wald ratio | 1 | 0.000 | 0.000 | 0.317 | 1.000 | 1.000(1.000-1.001) |
| BAFF-R on IgD- CD24- B cell | Inverse variance weighted | 7 | -0.001 | 0.001 | 0.358 | 1.000 | 0.999(0.998-1.001) |
| CD28 on activated & secreting CD4 regulatory T cell | Wald ratio | 1 | -0.037 | 0.100 | 0.713 | 1.000 | 0.964(0.792-1.173) |
| CD39+ secreting CD4 regulatory T cell %secreting CD4 regulatory T cell | Inverse variance weighted | 7 | 0.036 | 0.066 | 0.588 | 1.000 | 1.037(0.910-1.181) |
| HLA DR on B cell | Inverse variance weighted | 6 | 0.103 | 0.053 | 0.054 | 1.000 | 1.108(0.998-1.230) |
| Natural Killer %CD3- lymphocyte | Inverse variance weighted | 5 | -0.022 | 0.063 | 0.731 | 1.000 | 0.978(0.864-1.108) |
| CD14 on CD14+ CD16- monocyte | Wald ratio | 1 | 0.027 | 0.135 | 0.841 | 1.000 | 1.027(0.788-1.339) |
| CD45RA on naive CD4+ T cell | Inverse variance weighted | 9 | 0.000 | 0.000 | 0.028 | 1.000 | 1.000(1.000-1.000) |
| CD25 on resting CD4 regulatory T cell | Wald ratio | 1 | 0.369 | 0.336 | 0.272 | 1.000 | 1.446(0.749-2.794) |
| CD20 on IgD+ CD24- B cell | Inverse variance weighted | 4 | -0.167 | 0.110 | 0.126 | 1.000 | 0.846(0.682-1.048) |
| CD45RA+ CD28- CD8+ T cell %T cell | Inverse variance weighted | 62 | 0.000 | 0.001 | 0.909 | 1.000 | 1.000(0.999-1.001) |
| Activated CD4 regulatory T cell Absolute Count | Inverse variance weighted | 2 | 0.064 | 0.094 | 0.493 | 1.000 | 1.066(0.887-1.282) |
| CD45 on Natural Killer | Inverse variance weighted | 2 | -0.091 | 0.334 | 0.784 | 1.000 | 0.913(0.475-1.755) |
| CD27 on IgD+ CD24+ B cell | Inverse variance weighted | 6 | 0.039 | 0.054 | 0.473 | 1.000 | 1.040(0.935-1.157) |
| HLA DR++ monocyte %leukocyte | Inverse variance weighted | 2 | -0.353 | 0.147 | 0.016 | 1.000 | 0.702(0.527-0.937) |
| CD28+ CD45RA- CD8+ T cell Absolute Count | Wald ratio | 1 | 0.000 | 0.000 | 0.617 | 1.000 | 1.000(1.000-1.000) |
| CD20 on IgD- CD38- B cell | Inverse variance weighted | 3 | 0.158 | 0.116 | 0.174 | 1.000 | 1.171(0.933-1.470) |
| CD28- CD4-CD8- T cell %T cell | Inverse variance weighted | 3 | 0.000 | 0.000 | 0.998 | 1.000 | 1.000(0.999-1.001) |
| CD25 on IgD+ CD24+ B cell | Inverse variance weighted | 2 | -0.076 | 0.086 | 0.375 | 1.000 | 0.927(0.784-1.096) |
| CD4+ T cell Absolute Count | Wald ratio | 1 | -0.476 | 0.274 | 0.082 | 1.000 | 0.622(0.364-1.063) |
| CD4 on CD39+ CD4+ T cell | Inverse variance weighted | 6 | 0.042 | 0.052 | 0.418 | 1.000 | 1.043(0.942-1.154) |
| CD19 on IgD+ CD38- unswitched memory B cell | Inverse variance weighted | 3 | -0.003 | 0.124 | 0.983 | 1.000 | 0.997(0.783-1.271) |
| CD39+ CD4+ T cell %CD4+ T cell | Inverse variance weighted | 5 | 0.030 | 0.064 | 0.642 | 1.000 | 1.030(0.909-1.166) |
| CD45RA- CD4+ T cell %CD4+ T cell | Inverse variance weighted | 3 | 0.000 | 0.000 | 0.369 | 1.000 | 1.000(0.999-1.000) |
| CD20 on CD24+ CD27+ B cell | Inverse variance weighted | 6 | 0.072 | 0.080 | 0.368 | 1.000 | 1.075(0.919-1.258) |
| FSC-A on monocyte | Inverse variance weighted | 2 | 0.050 | 0.203 | 0.806 | 1.000 | 1.051(0.706-1.565) |
| CD8+ and CD8dim T cell %leukocyte | Inverse variance weighted | 2 | -0.058 | 0.181 | 0.748 | 1.000 | 0.943(0.661-1.346) |
| CD33 on Immature Myeloid-Derived Suppressor Cells | Inverse variance weighted | 6 | -0.016 | 0.026 | 0.543 | 1.000 | 0.984(0.935-1.036) |
| CX3CR1 on CD14+ CD16- monocyte | Inverse variance weighted | 2 | 0.000 | 0.000 | 0.317 | 1.000 | 1.000(0.999-1.000) |
| Activated & secreting CD4 regulatory T cell Absolute Count | Inverse variance weighted | 3 | 0.000 | 0.000 | 0.618 | 1.000 | 1.000(1.000-1.000) |
| CD11b on CD14+ monocyte | Wald ratio | 1 | 0.000 | 0.000 | 0.317 | 1.000 | 1.000(1.000-1.000) |
| CD28- CD8dim T cell %T cell | Inverse variance weighted | 3 | -0.001 | 0.001 | 0.475 | 1.000 | 0.999(0.998-1.001) |
| CD33 on CD66b++ myeloid cell | Inverse variance weighted | 4 | -0.013 | 0.039 | 0.746 | 1.000 | 0.987(0.915-1.066) |
| CD8dim T cell %leukocyte | Inverse variance weighted | 2 | -0.268 | 0.165 | 0.105 | 1.000 | 0.765(0.553-1.057) |
| FSC-A on myeloid Dendritic Cell | Inverse variance weighted | 3 | 0.000 | 0.000 | 0.996 | 1.000 | 1.000(0.999-1.001) |
| CD28 on CD39+ CD8+ T cell | Inverse variance weighted | 2 | -0.006 | 0.041 | 0.881 | 1.000 | 0.994(0.917-1.077) |
| CD3 on CD39+ resting CD4 regulatory T cell | Wald ratio | 1 | 0.000 | 0.000 | 1.000 | 1.000 | 1.000(0.999-1.001) |
| HLA DR+ Natural Killer %CD3- lymphocyte | Inverse variance weighted | 3 | 0.000 | 0.000 | 0.665 | 1.000 | 1.000(1.000-1.000) |
| CX3CR1 on CD14- CD16- | Wald ratio | 1 | 0.247 | 0.193 | 0.201 | 1.000 | 1.280(0.877-1.867) |
| HLA DR on CD14+ monocyte | Inverse variance weighted | 4 | -0.116 | 0.063 | 0.065 | 1.000 | 0.891(0.788-1.007) |
| FSC-A on CD4+ T cell | Inverse variance weighted | 2 | 0.295 | 0.232 | 0.203 | 1.000 | 1.344(0.853-2.117) |
| CD14+ CD16- monocyte Absolute Count | Inverse variance weighted | 3 | -0.049 | 0.200 | 0.806 | 1.000 | 0.952(0.643-1.410) |
| FSC-A on Natural Killer | Inverse variance weighted | 3 | 0.000 | 0.001 | 0.995 | 1.000 | 1.000(0.999-1.001) |
| CD24 on unswitched memory B cell | Inverse variance weighted | 2 | -0.034 | 0.193 | 0.862 | 1.000 | 0.967(0.662-1.413) |
| CD25 on switched memory B cell | Inverse variance weighted | 2 | 0.051 | 0.185 | 0.782 | 1.000 | 1.052(0.733-1.511) |
| CD25 on CD4+ T cell | Wald ratio | 1 | 0.337 | 0.322 | 0.295 | 1.000 | 1.401(0.745-2.635) |
| CD14 on CD14+ CD16+ monocyte | Inverse variance weighted | 2 | 0.000 | 0.001 | 0.999 | 1.000 | 1.000(0.999-1.001) |
| CD8 on CD8+ T cell | Inverse variance weighted | 3 | 0.000 | 0.001 | 0.999 | 1.000 | 1.000(0.999-1.001) |
| HVEM on Effector Memory CD8+ T cell | Wald ratio | 1 | 0.000 | 0.000 | 0.317 | 1.000 | 1.000(1.000-1.001) |
| CD19 on unswitched memory B cell | Inverse variance weighted | 3 | -0.003 | 0.166 | 0.985 | 1.000 | 0.997(0.721-1.379) |
| CD20 on IgD+ CD38- naive B cell | Wald ratio | 1 | -0.138 | 0.224 | 0.538 | 1.000 | 0.871(0.562-1.352) |
| CD3 on Naive CD4+ T cell | Inverse variance weighted | 4 | 0.000 | 0.000 | 0.721 | 1.000 | 1.000(1.000-1.000) |
| CD28+ CD45RA+ CD8dim T cell Absolute Count | Inverse variance weighted | 7 | 0.000 | 0.000 | 0.425 | 1.000 | 1.000(1.000-1.001) |
| CD28 on CD4 regulatory T cell | Wald ratio | 1 | -0.048 | 0.115 | 0.679 | 1.000 | 0.953(0.761-1.195) |
| CD38 on IgD+ B cell | Wald ratio | 1 | -0.175 | 0.214 | 0.414 | 1.000 | 0.839(0.552-1.277) |
| BAFF-R on CD20- B cell | Wald ratio | 1 | -0.019 | 0.127 | 0.884 | 1.000 | 0.982(0.766-1.258) |
| CD27 on Plasma Blast-Plasma Cell | Wald ratio | 1 | -0.001 | 0.001 | 0.317 | 1.000 | 0.999(0.998-1.001) |
| CD28 on CD28+ CD45RA- CD8+ T cell | Wald ratio | 1 | 0.045 | 0.103 | 0.664 | 1.000 | 1.046(0.855-1.278) |
| HVEM on Central Memory CD8+ T cell | Wald ratio | 1 | 0.000 | 0.000 | 0.317 | 1.000 | 1.000(1.000-1.001) |
| Unswitched memory B cell %B cell | Wald ratio | 1 | 0.162 | 0.346 | 0.641 | 1.000 | 1.176(0.596-2.318) |
| FSC-A on CD14+ monocyte | Wald ratio | 1 | -0.291 | 0.312 | 0.352 | 1.000 | 0.748(0.405-1.380) |
| CD19 on switched memory B cell | Inverse variance weighted | 2 | 0.069 | 0.167 | 0.678 | 1.000 | 1.072(0.773-1.486) |
| CCR2 on CD62L+ myeloid Dendritic Cell | Inverse variance weighted | 3 | 0.000 | 0.000 | 1.000 | 1.000 | 1.000(0.999-1.001) |
| CCR2 on monocyte | Inverse variance weighted | 3 | 0.000 | 0.000 | 0.317 | 1.000 | 1.000(1.000-1.001) |
| Resting CD4 regulatory T cell %CD4+ T cell | Inverse variance weighted | 4 | 0.000 | 0.000 | 0.390 | 1.000 | 1.000(1.000-1.001) |
| CCR7 on naive CD8+ T cell | Wald ratio | 1 | 0.082 | 0.145 | 0.574 | 1.000 | 1.085(0.816-1.443) |
| Natural Killer %lymphocyte | Inverse variance weighted | 3 | 0.111 | 0.183 | 0.544 | 1.000 | 1.117(0.781-1.597) |
| CD4-CD8- Natural Killer T %T cell | Inverse variance weighted | 5 | 0.035 | 0.090 | 0.696 | 1.000 | 1.036(0.869-1.235) |
| IgD on IgD+ CD38- unswitched memory B cell | Wald ratio | 1 | 0.030 | 0.079 | 0.699 | 1.000 | 1.031(0.884-1.202) |
| CD3 on CD8+ T cell | Inverse variance weighted | 2 | 0.000 | 0.000 | 1.000 | 1.000 | 1.000(1.000-1.000) |
| CD127 on CD4+ T cell | Wald ratio | 1 | 0.106 | 0.188 | 0.574 | 1.000 | 1.112(0.769-1.608) |
| CD127 on CD28+ CD45RA+ CD8+ T cell | Wald ratio | 1 | 0.035 | 0.301 | 0.907 | 1.000 | 1.036(0.574-1.869) |
| CD40 on CD14+ CD16+ monocyte | Inverse variance weighted | 4 | 0.000 | 0.000 | 0.356 | 1.000 | 1.000(1.000-1.001) |
| BAFF-R on CD24+ CD27+ B cell | Inverse variance weighted | 9 | 0.017 | 0.089 | 0.849 | 1.000 | 1.017(0.855-1.210) |
| CD45RA on CD39+ resting CD4 regulatory T cell | Inverse variance weighted | 2 | 0.054 | 0.094 | 0.567 | 1.000 | 1.055(0.878-1.269) |
| CD19 on IgD- CD38dim B cell | Inverse variance weighted | 2 | 0.016 | 0.221 | 0.941 | 1.000 | 1.017(0.659-1.569) |
| CD24+ CD27+ B cell Absolute Count | Wald ratio | 1 | 0.233 | 0.442 | 0.598 | 1.000 | 1.262(0.531-3.001) |
| CD24 on IgD+ CD38- unswitched memory B cell | Inverse variance weighted | 2 | -0.025 | 0.158 | 0.874 | 1.000 | 0.975(0.716-1.329) |
| CD20 on transitional B cell | Inverse variance weighted | 2 | 0.093 | 0.103 | 0.367 | 1.000 | 1.098(0.897-1.344) |
| CD4+CD8+ T cell %T cell | Wald ratio | 1 | 0.081 | 0.104 | 0.438 | 1.000 | 1.084(0.884-1.330) |
| CD28+ CD45RA+ CD8+ T cell Absolute Count | Inverse variance weighted | 2 | 0.493 | 0.756 | 0.515 | 1.000 | 1.636(0.372-7.203) |
| CD25 on CD45RA- CD4 not regulatory T cell | Wald ratio | 1 | 0.213 | 0.204 | 0.295 | 1.000 | 1.237(0.830-1.844) |
| CD3 on secreting CD4 regulatory T cell | Inverse variance weighted | 3 | 0.000 | 0.000 | 1.000 | 1.000 | 1.000(1.000-1.000) |
| CD16-CD56 on Natural Killer T | Inverse variance weighted | 4 | 0.083 | 0.077 | 0.281 | 1.000 | 1.086(0.934-1.263) |
| IgD on IgD+ CD38dim B cell | Inverse variance weighted | 5 | -0.013 | 0.061 | 0.834 | 1.000 | 0.987(0.876-1.113) |
| CCR2 on granulocyte | Wald ratio | 1 | -0.680 | 0.378 | 0.072 | 1.000 | 0.506(0.241-1.062) |
| CD28 on CD39+ secreting CD4 regulatory T cell | Inverse variance weighted | 2 | 0.053 | 0.072 | 0.464 | 1.000 | 1.054(0.915-1.214) |
| CD45RA+ CD28- CD8+ T cell %CD8+ T cell | Wald ratio | 1 | 0.000 | 0.000 | 1.000 | 1.000 | 1.000(1.000-1.000) |
| CD25 on IgD- CD24- B cell | Inverse variance weighted | 2 | 0.249 | 0.156 | 0.110 | 1.000 | 1.283(0.945-1.743) |
| CD45 on B cell | Wald ratio | 1 | -0.103 | 0.215 | 0.631 | 1.000 | 0.902(0.591-1.375) |
| CD38 on transitional B cell | Inverse variance weighted | 5 | -0.001 | 0.001 | 0.310 | 1.000 | 0.999(0.998-1.001) |
| Terminally Differentiated CD4+ T cell Absolute Count | Inverse variance weighted | 2 | 0.001 | 0.001 | 0.355 | 1.000 | 1.001(0.999-1.003) |
| CD25 on CD39+ CD4 regulatory T cell | Wald ratio | 1 | -0.192 | 0.169 | 0.254 | 1.000 | 0.825(0.593-1.148) |
| IgD on transitional B cell | Inverse variance weighted | 4 | -0.030 | 0.077 | 0.691 | 1.000 | 0.970(0.835-1.127) |
| CD20 on IgD+ CD38dim B cell | Inverse variance weighted | 8 | -0.146 | 0.075 | 0.050 | 1.000 | 0.864(0.747-1.000) |
| HLA DR++ monocyte %monocyte | Inverse variance weighted | 2 | -0.313 | 0.130 | 0.016 | 1.000 | 0.731(0.566-0.944) |
| CD45RA+ CD8+ T cell %T cell | Wald ratio | 1 | 0.001 | 0.001 | 0.046 | 1.000 | 1.001(1.000-1.002) |
| CD33 on CD14+ monocyte | Inverse variance weighted | 5 | -0.005 | 0.028 | 0.853 | 1.000 | 0.995(0.941-1.051) |
| CD24+ CD27+ B cell %lymphocyte | Inverse variance weighted | 3 | 0.108 | 0.170 | 0.523 | 1.000 | 1.114(0.799-1.554) |
| CD33+ HLA DR+ Absolute Count | Wald ratio | 1 | -0.001 | 0.085 | 0.987 | 1.000 | 0.999(0.845-1.181) |
| Activated & secreting CD4 regulatory T cell %CD4+ T cell | Inverse variance weighted | 7 | 0.000 | 0.000 | 0.379 | 1.000 | 1.000(1.000-1.000) |
| SSC-A on HLA DR+ Natural Killer | Inverse variance weighted | 3 | 0.000 | 0.000 | 0.775 | 1.000 | 1.000(1.000-1.000) |
| CD4-CD8- T cell Absolute Count | Inverse variance weighted | 2 | 0.000 | 0.000 | 0.999 | 1.000 | 1.000(0.999-1.001) |
| Secreting CD4 regulatory T cell %CD4 regulatory T cell | Inverse variance weighted | 5 | 0.000 | 0.000 | 0.644 | 1.000 | 1.000(1.000-1.000) |
| CD28- CD8dim T cell Absolute Count | Inverse variance weighted | 2 | -0.001 | 0.001 | 0.506 | 1.000 | 0.999(0.997-1.001) |
| Plasmacytoid Dendritic Cell %Dendritic Cell | Inverse variance weighted | 3 | 0.000 | 0.000 | 0.726 | 1.000 | 1.000(1.000-1.000) |
| CD20 on IgD+ CD24+ B cell | Inverse variance weighted | 2 | 0.063 | 0.134 | 0.637 | 1.000 | 1.065(0.820-1.384) |
| HVEM on CD4+ T cell | Wald ratio | 1 | 0.000 | 0.000 | 0.317 | 1.000 | 1.000(1.000-1.001) |
| CD3 on CD28+ CD4+ T cell | Inverse variance weighted | 2 | 0 | NA | NA | NA | 1.000(NA-NA) |
| CD62L on CD62L+ Dendritic Cell | Inverse variance weighted | 2 | 0 | NA | NA | NA | 1.000(NA-NA) |
| CD3 on T cell | Inverse variance weighted | 2 | 0 | NA | NA | NA | 1.000(NA-NA) |

MR,Mendelian randomization;SNP,Single-nucleotide polymorphism;Beta,beta regression coefficient;Se,standard error;(95%CI),95%Confidence interval;OR, Odds ratio; Pval,P value;FDR,False Discovery Rate.

Appendix Table 11. Results of Heterogeneity and Pleiotropy Analysis of Variable Risk Factors and oral cancer

| Exposure | Outcome | Method | Q | Q_df | Q_pval | pleiotropy（pval） |
| --- | --- | --- | --- | --- | --- | --- |
| Daytime nap | Oral Cancer | MR Egger | 74.272 | 83 | 0.742 | 0.236 |
| Daytime nap | Oral Cancer | Inverse variance weighted | 75.699 | 84 | 0.729 |  |
| Histidine | Oral Cancer | MR Egger | 6.698 | 9 | 0.668 | 0.466 |
| Histidine | Oral Cancer | Inverse variance weighted | 7.279 | 10 | 0.699 |  |
| Cigarettes per Day | Oral Cancer | MR Egger | 18.734 | 18 | 0.408 | 0.601 |
| Cigarettes per Day | Oral Cancer | Inverse variance weighted | 19.029 | 19 | 0.455 |  |
| Testosterone levels | Oral Cancer | MR Egger | 73.193 | 81 | 0.720 | 0.723 |
| Testosterone levels | Oral Cancer | Inverse variance weighted | 73.319 | 82 | 0.742 |  |
| Current tobacco smoking | Oral Cancer | MR Egger | 9.339 | 13 | 0.747 | 0.213 |
| Current tobacco smoking | Oral Cancer | Inverse variance weighted | 11.056 | 14 | 0.682 |  |
| Alcohol intake versus 10 years previously | Oral Cancer | MR Egger | 8.461 | 5 | 0.133 | 0.322 |
| Alcohol intake versus 10 years previously | Oral Cancer | Inverse variance weighted | 10.501 | 6 | 0.105 |  |
| Effector Memory CD4+ T cell %T cell | Oral Cancer | NA | NA | NA | NA | NA |
| CD45RA+ CD8+ T cell Absolute Count | Oral Cancer | NA | NA | NA | NA | NA |
| CD4 on HLA DR+ CD4+ T cell | Oral Cancer | NA | NA | NA | NA | NA |
| CD8 on CD28+ CD45RA- CD8+ T cell | Oral Cancer | NA | NA | NA | NA | NA |
| CD28 on CD45RA- CD4 not regulatory T cell | Oral Cancer | NA | NA | NA | NA | NA |
| CD8+ T cell %leukocyte | Oral Cancer | NA | NA | NA | NA | NA |

Pleiotropy, Pleiotropy Test; Q_pval, horizontal pleiotropy P-value for Q-test;

Appendix Table 12. Results of Mediation Analysis from Protein Levels to OC via immune cells

| **Exposure** | **Mediator** | **Total Effect β (95% CI)** | **Direct Effect β1 (95% CI)** | **Direct Effect β2 (95% CI)** | **Mediated effect（β1*β2）** | **Mediated effect ratios（β1*β2/β）*100%** | **Total Pval** | **Direct Pval** | **Direct Pval 2** |
| --- | --- | --- | --- | --- | --- | --- | --- | --- | --- |
| CD4 on HLA DR+ CD4+ T cell | HSD17B14 | 0.77(1.48-3.18) | 0.08(1.01-1.16) | 0.69(1.02-3.91) | 0.06 | 7.18% | 7.73E-05 | 0.03 | 0.04 |
| Cigarettes per Day | TNFSF8 | 0.39(1.16-1.87) | 0.09(1.00-1.21) | 0.84(1.72-3.11) | 0.08 | 20.63% | 1.61E-03 | 0.05 | 2.49E-08 |
| CD8+ T cell %leukocyte | TNFSF8 | 0.65(1.37-2.69) | 0.09(1.00-1.20) | 0.84(1.72-3.11) | 0.08 | 12.06% | 1.36E-04 | 0.05 | 2.49E-08 |

MR, Mendelian randomization;(95%CI), 95%Confidence interval;OC, Oral caner;β, Variable - outcome effect value;β1, Modifiable factor-protein effect value;β2, protein-outcome effect value;

Appendix Table 13. PEHWAS-MR results for 3 plasma proteins(positive results)

| Outcome | Exposure | Method | nsnp | Beta | Se | Pval | Pval-FDR |
| --- | --- | --- | --- | --- | --- | --- | --- |
| Trunk fat percentage | XXYLT1 | Wald ratio | 1 | 0.04 | 0.01 | 0.00 | 0.01 |
| Body fat percentage | XXYLT1 | Wald ratio | 1 | 0.04 | 0.01 | 0.00 | 0.01 |
| Trunk fat mass | XXYLT1 | Wald ratio | 1 | 0.05 | 0.01 | 0.00 | 0.01 |
| Body mass index (BMI) | XXYLT1 | Wald ratio | 1 | 0.04 | 0.01 | 0.00 | 0.01 |
| Whole body fat mass | XXYLT1 | Wald ratio | 1 | 0.04 | 0.01 | 0.00 | 0.01 |
| Reason for glasses/contact lenses: For just reading/near work as you are getting older (called 'presbyopia') | XXYLT1 | Wald ratio | 1 | 0.01 | 0.00 | 0.00 | 0.02 |
| Leg fat percentage (left) | XXYLT1 | Wald ratio | 1 | 0.03 | 0.01 | 0.00 | 0.02 |
| Arm fat mass (left) | XXYLT1 | Wald ratio | 1 | 0.04 | 0.01 | 0.00 | 0.02 |
| Hip circumference | XXYLT1 | Wald ratio | 1 | 0.04 | 0.01 | 0.00 | 0.02 |
| Weight | XXYLT1 | Wald ratio | 1 | 0.03 | 0.01 | 0.00 | 0.02 |
| Leg fat mass (left) | XXYLT1 | Wald ratio | 1 | 0.03 | 0.01 | 0.00 | 0.02 |
| Arm fat percentage (left) | XXYLT1 | Wald ratio | 1 | 0.03 | 0.01 | 0.00 | 0.02 |
| Leg fat percentage (right) | XXYLT1 | Wald ratio | 1 | 0.03 | 0.01 | 0.00 | 0.02 |
| Leg fat mass (right) | XXYLT1 | Wald ratio | 1 | 0.03 | 0.01 | 0.00 | 0.02 |
| Arm fat percentage (right) | XXYLT1 | Wald ratio | 1 | 0.03 | 0.01 | 0.00 | 0.02 |
| Arm fat mass (right) | XXYLT1 | Wald ratio | 1 | 0.04 | 0.01 | 0.00 | 0.02 |
| Ever smoked | XXYLT1 | Wald ratio | 1 | -0.02 | 0.01 | 0.00 | 0.02 |
| Ankle spacing width | XXYLT1 | Wald ratio | 1 | 0.04 | 0.01 | 0.00 | 0.02 |
| Comparative body size at age 10 | XXYLT1 | Wald ratio | 1 | 0.03 | 0.01 | 0.00 | 0.03 |
| Impedance of whole body | HSD17B14 | Wald ratio | 1 | -0.09 | 0.02 | 0.00 | 0.04 |
| Cheese spread intake | HSD17B14 | Wald ratio | 1 | -0.08 | 0.02 | 0.00 | 0.04 |
| Mouth/teeth dental problems: Mouth ulcers | TNFSF8 | Wald ratio | 1 | 0.02 | 0.00 | 0.00 | 0.00 |
| Blood clot, DVT, bronchitis, emphysema, asthma, rhinitis, eczema, allergy diagnosed by doctor: None of the above | TNFSF8 | Wald ratio | 1 | -0.03 | 0.01 | 0.00 | 0.01 |
| Non-cancer illness code, self-reported: hayfever/allergic rhinitis | TNFSF8 | Wald ratio | 1 | 0.01 | 0.00 | 0.00 | 0.02 |
| PCT where patients GP was registered: SOUTH TYNESIDE PCT | TNFSF8 | Wald ratio | 1 | 0.00 | 0.00 | 0.00 | 0.03 |
| Doctor diagnosed hayfever or allergic rhinitis | TNFSF8 | Wald ratio | 1 | 0.05 | 0.01 | 0.00 | 0.04 |
| Type of cancer: ICD10: C44.3 Skin of other and unspecified parts of face | TNFSF8 | Wald ratio | 1 | -0.01 | 0.00 | 0.00 | 0.04 |

SNP,Single nucleotide polymorphism;Beta,beta regression coefficient;Se,standard error;(95%CI),95%Confidence interval;OR,Odds ratio;Pval,P-value; FDR,False Discovery Rate.
